# Photosensitizer Repositioning Affords an Enantiocomplementary Enzyme for [2 + 2]‐Cycloadditions

**DOI:** 10.1002/anie.202503576

**Published:** 2025-07-04

**Authors:** Chuanjie Sun, Anna R. Kohn, Ross Smithson, Florence J. Hardy, Jonathan S. Trimble, Yuanxin Cao, Linus O. Johannissen, Sam Hay, Rebecca Crawshaw, Anthony P. Green

**Affiliations:** ^1^ Department of Chemistry & Manchester Institute of Biotechnology The University of Manchester 131 Princess Street Manchester M1 7DN UK

**Keywords:** Biocatalysis, Directed evolution, Genetic code expansion, Photocatalysis, Protein engineering

## Abstract

The combination of genetic code expansion and directed evolution has recently given rise to enantioselective photoenzymes for [2 + 2]‐cycloadditions of quinolone and indole derivatives. However, the enzymes reported to date only allow access to one enantiomeric series of the strained cyclobutane products. Here, guided by a crystal structure of our previously engineered enzyme EnT1.3, we show how judicious repositioning of the genetically programmed benzophenone photosensitizer affords an enantiocomplementary [2 + 2]‐cyclase, CEnT1.0. Following directed evolution, a proficient and oxygen‐tolerant photoenzyme (CEnT1.4) emerged that promotes [2 + 2]‐cycloadditions of a quinolone derivative with exquisite enantiocontrol (99% *e.e*.) and substantially enhanced regioselectivity compared with EnT1.3 (r.r. 62:1 vs. 9:1). Structural analysis of CEnT1.4, coupled with molecular dynamic simulations, reveals a well‐sculpted active site pocket that pre‐organises the substrate for regio‐ and enantioselective catalysis. This study highlights the versatility offered by genetically programmed (photo)catalytic elements when developing enzymes for altered stereochemical outcomes.

## Graphical abstract

In this study, we capitalize on the versatility offered by genetic code expansion to develop a proficient photoenzyme for [2 + 2]‐cycloadditions that is enantiocomplementary to our recently developed photoenzyme EnT1.3. By repositioning the genetically programmed benzophenone photosensitizer and subsequent directed evolution, we have developed an efficient, highly selective and oxygen tolerant enzyme for a range of [2 + 2]‐cycloadditions.

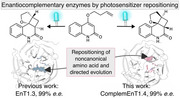

## Introduction

Genetic code expansion (GCE) allows new functional motifs to be covalently embedded into proteins as non‐canonical amino acid (ncAA) side chains.^[^ ^1^, ^2^ ^]^ In recent years, this technology has enabled the development of new enzymes with mechanisms and catalytic functions that are beyond those found in nature.^[^ ^3^, ^4^, ^5^ ^]^ For example, metal coordinating ncAAs have been used to augment the properties of heme enzymes and to develop artificial metalloenzymes for a variety of transformations.^[^ ^6^, ^7^, ^8^, ^9^, ^10^, ^11^, ^12^ ^]^ Enzymes have also been developed that rely on ncAAs with organocatalytic side chains as a key functional motifs.^[^ ^13^, ^14^, ^15^, ^16^ ^]^

More recently, a combination of GCE and laboratory evolution has given rise to enantioselective and oxygen‐tolerant photoenzymes for thermally forbidden [2 + 2]‐cycloadditions.^[^ ^17^, ^18^ ^]^ These enzymes make use of 4‐benzoylphenylalanine (BpA) as a genetically encoded triplet photosensitizer.^[^ ^19^ ^]^ Irradiation with UV light leads to the formation of a long‐lived BpA (or benzophenone) triplet excited state owing to efficient intersystem crossing (ISC) from the transiently formed singlet excited state. ^[^ ^20^, ^21^ ^]^ The photochemical energy stored can then be transferred to a nearby substrate in an overall spin‐allowed process (Dexter Energy Transfer) to generate a triplet excited state that is sufficiently long‐lived for downstream chemistry to occur. Performing such energy transfer photocatalysis within tuneable chiral protein environments allows stereoselective transformations to be achieved, including those which have proven challenging with small chiral photocatalysts. Our own lab used this approach to develop photoenzymes (EnT1.3^[^ ^17^ ^]^) for enantioselective [2 + 2]‐cycloadditions of quinolone derivatives by positioning BpA into the hydrophobic pocket of the computationally designed Diels‐Alderase DA_20_00.^[^ ^22^ ^]^ In a simultaneous report from Sun et al., a similar approach was used to develop enantioselective enzymes for [2 + 2]‐cycloadditions of indole derivatives by incorporating BpA photosensitizer into the central pore of the dimeric Lactococcal multidrug resistance Regulator (LmrR) protein. ^[^ ^18^ ^]^

Although powerful enantioselective photocatalysts, the EnT photoenzymes reported to date only allow access to one enantiomeric series of the strained cyclobutane products.^[^ ^17^, ^18^ ^]^ To maximize synthetic utility, it is important to develop stereo‐complementary enzymes that allow access to either enantiomer of chiral substituted cyclobutanes. In this study, we capitalize on the flexibility offered by GCE to reposition the BpA photosensizer within the active site of DA_20_00. This modification enabled the development of a photoenzyme, CEnT1.4, that is enantiocomplementary to our recently reported enzyme EnT1.3 and offers a greater degree of control over the regiochemistry of cycloaddition.

## Results and Discussion

**Figure 1:**
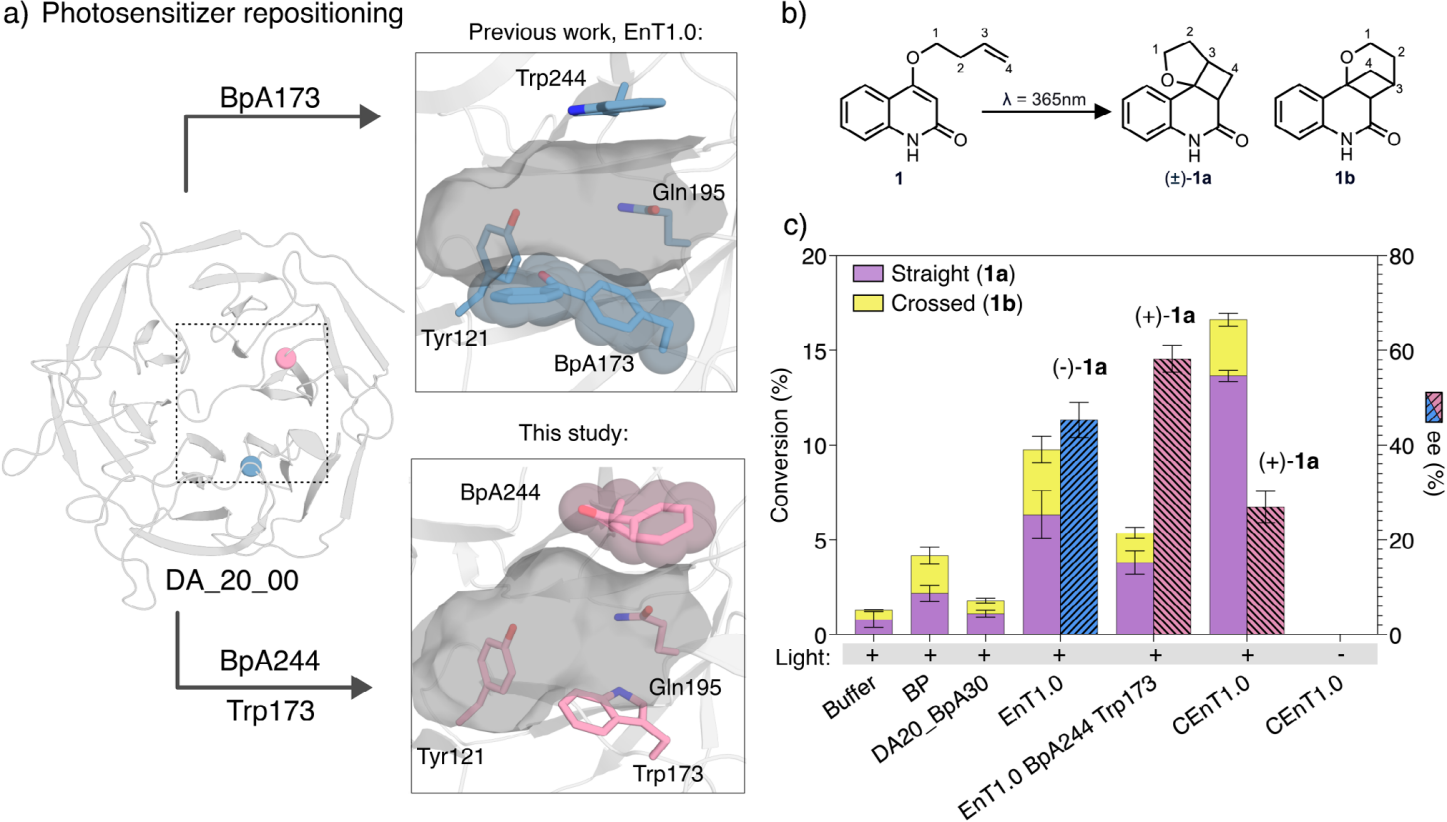
Development of enantiocomplementary photoenzymes for [2 + 2]‐cycloadditions. a) Positioning the non‐canonical amino acid photosensitizer 4‐benzoylphenylalanine (BpA) in the hydrophobic pocket of the beta‐propeller protein DA_20_00^[^ ^17^ ^]^ (PDB: 3I1C) gives the enantiocomplementary enzymes EnT1.0 (PDB: 7ZP5) and EnT1.0 BpA244 Trp173 (adapted from PDB: 7ZP5). The protein backbone is shown as a grey cartoon with blue (EnT1.0) or pink (CEnT1.0) sticks and semi‐transparent CPK spheres. b) A chemical scheme showing the intramolecular [2 + 2] photocycloaddition of 4‐(but‐3‐en‐1‐yloxy)quinolin‐2(1H)‐one (**1**), which gives rise to two regioisomeric products, 3,3a,4,4a‐tetrahydro‐2H‐furo[2′,3′:2,3]cyclobuta[1,2‐c]quinolin‐5(6H)‐one (**1a**) and 2,3,4,4a‐tetrahydro‐4,10b‐methanopyrano[3,2‐c]quinolin‐5(6H)‐one (**1b**). c) Bar chart showing the conversion of **1** to **1a** and **1b** by benzophenone (BP), DA_20_00 30BpA (an inactive variant with the BpA on the surface of the protein), EnT1.0 (DA_20_00 173BpA), EnT1.0 BpA244 173Trp, CEnT1.0 (DA_20_00 BpA244 Ala173). Reaction conditions: 15 µM catalyst (BP or protein variant), 400 µM **1**, 30 min irradiation at 365 nm, 4 °C. CEnT1.0 activity is strictly dependent on light. All conversion and selectivity data, including standard deviations, are given in Table S1. Error bars represent the standard deviation of measurements made in triplicate.

Our previously reported [2 + 2]‐cyclase, EnT1.3, was engineered to mediate regio‐ and enantio‐selective [2 + 2]‐cycloaddition of quinolone **1**, and affords (‐)‐**1a** with impressive stereocontrol (r.r. 9:1, >99% *e.e*., Figure 1). ^[^ ^17^ ^]^ The product bound crystal structure of EnT1.3 reveals that the ligand sits within a well‐sculpted/snug active site pocket in close proximity to the benzophenone side chain, facilitating efficient energy transfer from the excited photosensitizer to the parent substrate (PDB: 7ZP7). Ligand binding is further supported by complementary hydrogen bonding interactions with the side chains of Gln195 and Tyr121. An active site Trp244 plays a crucial role in controlling reaction selectivity by blocking one face of a quinolone substrate to guide facial selectivity of cycloaddition.

Given the importance of BpA173 and Trp244 in shaping the substrate binding pocket and controlling reaction selectivity, we reasoned that switching of positioning of these two bulky aromatic residues could give rise to an enantiocomplementary photoenzyme. Previous studies have shown how repositioning of catalytic side chains or metal chelating residues within enzyme active sites can give rise of altered stereochemical outcomes.^[^ ^23^, ^24^, ^25^, ^26^ ^]^ Pleasingly, irradiation of the EnT1.0 BpA244 Trp173 variant in the presence of substrate **1** led to the production of (+)‐**1a** (58% *e.e*.), the opposite stereocontrol achieved with EnT1.3, with modest levels of regiocontrol (r.r. 2.4:1 **1a**:**1b**, Figure 1c). Although the regioselectivity and enantioselectivity achieved by EnT1.0 BpA244 Trp173 is higher than the parent EnT1.0, its activity and expression levels are somewhat compromised.^[^ ^17^ ^]^ Interestingly, these issues can be resolved with a Trp173Ala mutation, which improves expression, activity and regiocontrol, while maintaining preference for formation of the target enantiomer, albeit with reduced enantioselectivity compared with EnT1.0 BpA244 Trp173. In light of its improved properties, this variant (termed CEnT1.0) was selected as a template for further engineering.

**Figure 2:**
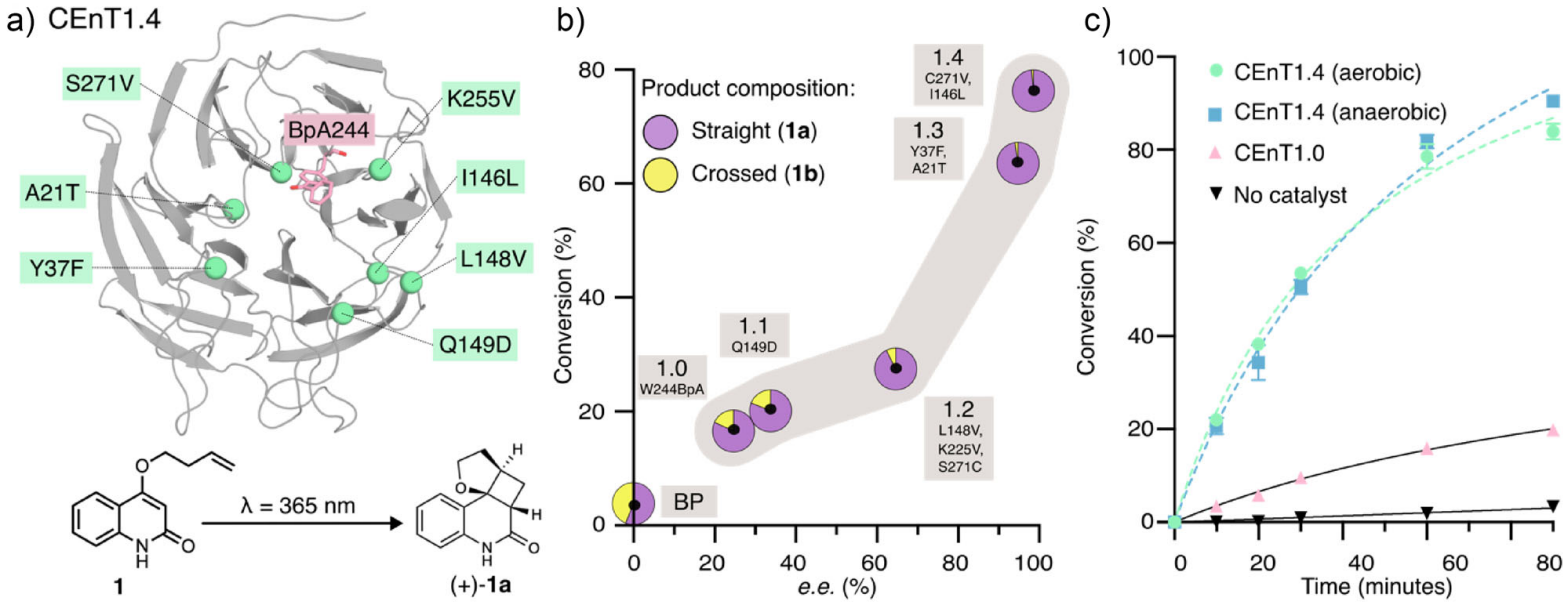
Directed evolution of an efficient and enantioselective photoenzyme. a) Mutational map of CEnT1.4, highlighting the encoded photosensitizer BpA244 (pink sticks) and residues installed during rational engineering and rounds 1, 2 and 3 of evolution (mutations are represented as spheres in green). b) Reaction conversion, regioselectivity and enantioselectivity were improved along the evolutionary trajectory. Reaction conditions: 10 µM (2.5 mol%) catalyst, 400 µM **1**, 60 min irradiation at 365 nm, 4 °C. The ratio of **1a** to **1b** is represented as a pie chart and *e.e*. is given for (‐)‐**1a**. All conversion and selectivity data, including standard deviations, are given in Table S2. c) Reaction time courses (**1** to **1a** and **1b**, 10 µM (2.5 mol%) catalyst, 400 µM **1**, 365 nm, 4 °C) catalysed by CEnT1.4 under aerobic conditions (green). A comparison of CEnT1.4 activity under anaerobic conditions is shown in blue. Error bars represent the standard deviation of measurements made in triplicate.

Following analysis of a handful of point mutants, selected based on their proximity to the BpA sensitizer and/or their beneficial impact on catalysis during EnT1.3 engineering,^[^ ^17^ ^]^ a Q149D mutation was identified that led to improved activity and selectivity. This CEnT1.1 variant served as a starting template for optimization over three rounds of directed evolution. In each round, ca. 20 residues located in the active site or second coordination sphere were individually randomized using NNK degenerate codons. Individual variants were arrayed in 96‐well microtiter plates and analysed by ultra‐performance liquid chromatography (UPLC) for the conversion of **1** to **1a**. Beneficial mutations identified in each round were combined through DNA shuffling. Following evaluation of ∼5700 clones, the variant CEnT1.4 emerged, containing seven mutations (CEnT1.0 A21T Y37F I146L L148V Q149D K225V S271V, Figures 2a, S1). CEnT1.4 achieves a 4.6‐fold higher conversion of **1** to **1a** compared with CEnT1.0 following 60 min of irradiation and shows excellent regio‐ and stereocontrol (62:1 **1a**:**1b**, 99% *e.e*., Figure 2b). This improvement arises from a 4‐fold increase in initial rate and a 14‐fold improvement in regioselectivity (Figure S2).

To gain insight into the kinetic parameters of CEnT1.4, we determined initial reaction velocities across a range of substrate concentrations (Figure S3A). CEnT1.4 has a V_max_ of 1.8 ± 0.14 min^−1^ under the assay conditions used for enzyme engineering, which compares favourably to EnT1.3 (Figures S3B, S4). This value increases linearly with light intensity (Figure S3C). The *K*_M_ for the substrate **1** is <55 µM. Similar to EnT1.3, CEnT1.4 is tolerant to aerobic conditions and operates efficiently at both 4 °C and room temperature, in contrast to small molecule triplet photosensitizers that typically require cryogenic and anaerobic conditions (Figures 2c, S5).^[^ ^27^ ^]^ Under aerobic conditions at 4 °C, CEnT1.4 enzyme achieves ∼ 150 turnovers (Figure S6). A semi‐preparative scale biotransformation (∼11 mg scale) gave optically pure **1a** in 98% conversion and 70% isolated yield (Figure S7).

**Figure 3:**
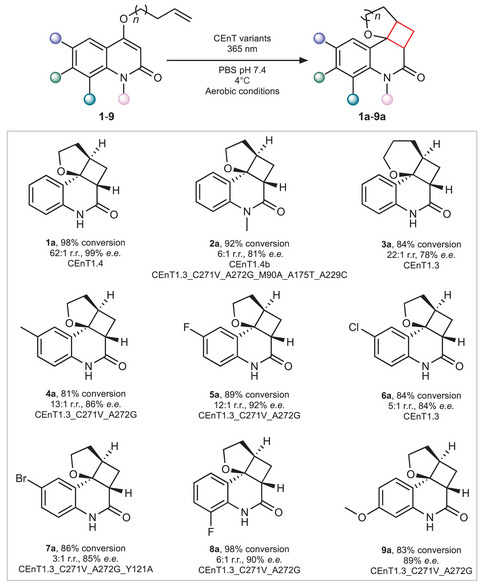
Substrate scope of CEnT variants. CEnT variants promote intramolecular [2 + 2]‐cycloadditions on a range of quinolones **1**–**9** with high selectivities and conversions. For substrate **2** featuring an N‐methyl substituent, we performed an additional round of engineering to generate the improved variant CEnT1.4b. Reaction conversions are the mean of biotransformations performed in triplicate. The absolute stereochemistry of **7a** was confirmed following reduction of enzymatically synthesized **7a** to optically enriched **1a** (Figures S8 and S9). The absolute stereochemistry of products **2a**–**6a, 8a and 9a** were tentatively assigned by analogy to the product (+)‐**1a**, formed by CEnT1.4. Reaction conditions for the synthesis of **1a**–**9a** are presented in Table S12.

We next explored the activity of CEnT1.4, and selected variants along the evolution trajectory, towards a series of quinolone analogs **2**–**9** (Figure 3). Substrates featuring C6, C7 and C8 quinolone substituents are generally well tolerated, furnishing optically enriched cycloaddition adducts equipped with functional handles that could be used for further synthetic manipulations. In the case of the C6‐Br derivative **7**, introduction of a Y121A mutation into CEnT1.3 C271V A272G led to improved activity and selectivity. A semi‐preparative scale biotransformation using this variant gave **7a** with > 99% conversion (70% isolated yield) and 84% e.e. (Figures S8, S9). Notably, conversion of substrate **3** also proceeds with appreciable levels of stereocontrol. This transformation has proven particularly challenging with small chiral photosensitizers as cyclizations to form six‐membered‐ring analogues are relatively slow and competing dissociation of the excited substrate from the photosensitizer erodes e.e.^[^ ^20^ ^][^ ^27^ ^]^ Unfortunately, following evaluation of variants from our initial directed evolution campaign, we were unable to identify variants for selective conversion of N‐alkyl derivative **2**, with only modest 14% e.e. achieved with CEnT1.3 C271V A272G. To address this limitation, we performed an additional round of directed evolution to afford a CEnT1.4b variant (CEnT1.3 C271V, A272G, M90A, A175T, A229C) that furnishes **2a** with 81% e.e. and 92% conversion.

**Figure 4:**
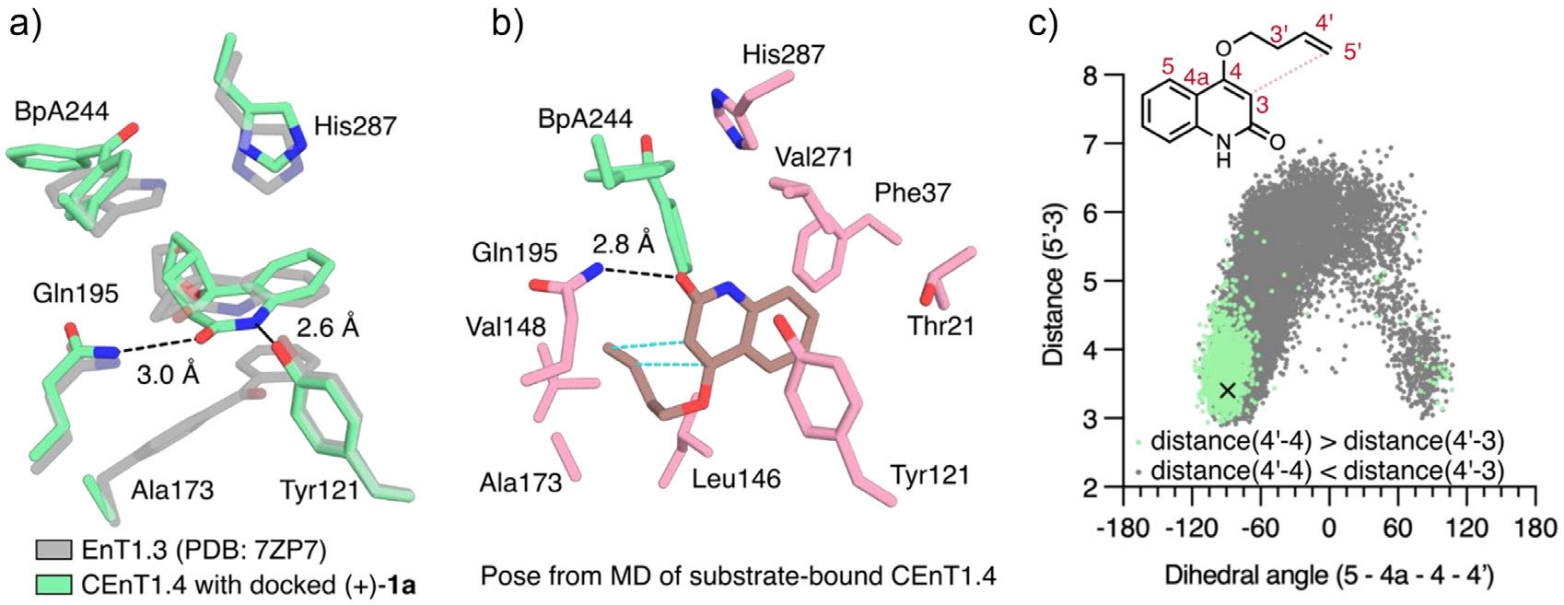
| Structural and computational analysis of CEnT1.4. a) An overlay of the active sites of CEnT1.4 (PDB: 9ENO) docked with product (+)‐**1a** and product (‐)‐**1a** bound EnT1.3 (PDB: 7ZP7), shown as atom‐coloured sticks with green and transparent grey carbons, respectively. Residue labels are given for CEnT1.4, and the bond distances are given between the active site residues and the docked product. b) A representative pose from the MD simulation of CEnT1.4 in complex with the substrate **1**. Key active site residues, BpA244, and the substrate **1** are shown as atom‐coloured sticks with pink, green and brown carbon atoms, respectively. A hydrogen bonding interaction is shown between Gln195 and the substrate as a black dashed line. Blue dashed lines show the bonds to form for the (+)‐**1a** product. c) A scatter plot showing the distance between carbons 5′ and 3 and dihedral angle between carbons 5–4a‐4–4′, from individual frames in the 600 ns MD simulation of CEnT1.4 with substrate **1**. The pose from Figure 4b is indicated as a black cross.

To gain insights into the CEnT1.4 catalytic mechanism, a 1.7 Å crystal structure of CEnT1.4 was solved (PDB: 9ENO, Table S3). This structure superimposes well with both DA_20_00 and EnT1.3 (C_α_ root mean square deviation 0.4 and 0.4, Figure S10). The BpA244 photosensitizer sits with the carbonyl group directed away from the bulk solvent, as in structures of EnT1.3. Molecular docking of the product (+)‐**1a** and the substrate **1** into the crystal structure of CEnT1.4 reveals binding geometries similar to those observed with (‐)‐**1a**‐bound EnT1.3, with **1** and (+)‐**1a** in close proximity to the BpA photosensitizer and forming hydrogen bonding interactions with Q195 and Y121 (Figure 4a). Molecular dynamics (MD) simulations performed on both complexes exhibit considerable conformational sampling of both the BpA and the substrate/product during the 600 ns simulation (Figures S11, S12). Conformers were identified where the quinoline moieties of **1** and (+)‐**1a** were largely superimposable, suggesting that the substrate is sampling conformations that can undergo cyclization to form (+)‐**1a**. The CEnT1.4–**1** simulations show a major population (∼90%) of the substrate pre‐organized for selective catalysis in a well‐sculpted active site pocket formed by hydrophobic residue F37, L146, V148, V225, V271 and BpA244 (Figure 4b). The substrate also forms a hydrogen bond with Q195, has a C H – π interaction (edge‐to‐face π stacking) with BpA244 but does not form hydrogen bonding contacts with Y121. A minor population (∼7%) involves the substrate in a geometry similar to that observed in the docking pose, which rapidly interconverts (<40 ps) with the major population.

A second population analysis was performed that grouped conformers of the substrate based on their potential C4 chirality (dihedral θ(5–4a‐4–4′)) and likelihood to form a straight versus cross product [bond *R*(4′‐4) < *R*(4′‐3)]. Consistent with experimental data, this analysis shows that the substrate samples conformations that should lead to the correct stereoisomer >90% of the time and that lead to formation of (+)‐**1a** 85%–89% of the time (Figure 4c, Table S4 and Figure S13). Similar behaviour and populations are observed in MD simulations performed with fluorinated substrate **5**, suggesting that the preferred stereochemical outcome is the same as that observed with **1** (Figures S12, S13). While the major population of **1** does not involve stacking of its quinoline moiety with BpA244, rapid interconversion between this and conformer(s) where π‐π stacking occurs suggests that energy transfer could plausibly precede substrate reorganization to form a reactive conformation. A separate analysis of the MD trajectory shows that the BpA and substrate adopt conformations expected to lead to efficient energy transfer in ∼7% of the frames (Figure S14). In agreement with the simulations, a Q195A mutation leads to a substantial reduction in activity and reaction selectivity, consistent with hydrogen bonding between this residue and the substrate being important for selective photocatalysis (Figure S15). In contrast, a Y121F mutation has negligible impact on activity or reaction selectivity, again supporting the simulations where there are hydrogen bonding interactions formed between Y121 and the substrate in the major population observed.

## Conclusion

In this study, we set out to develop an enantiocomplementary enzyme for photochemical [2 + 2]‐cycloadditions, in order to expand the synthetic utility of an emerging class of triplet energy transfer photoenzymes. Guided by a crystal structure of our previously engineered enzyme EnT1.3, we show how judicious repositioning of the genetically programmed BpA photosensitizer leads to a switch in stereochemical outcome for the conversion of **1** to **1a**.  Further engineering through directed evolution afforded a proficient photoenzyme, CEnT1.4, that displays exceptional levels of enantiocontrol (99% *e.e*.) and significantly enhanced regio‐control compared with EnT1.3. Structural analysis and MD simulations provide detailed insights into the enzyme mechanism and origins of selective photocatalysis.

This study illustrates the flexibility offered by GCE when creating new catalytic sites within proteins, whereby key functional elements can be accurately positioned at multiple sites within diverse protein cavities at will. This contrasts with the situation encountered with non‐covalent cofactors that cannot be readily repositioned and can only be placed within a limited range of protein scaffolds. Using our methods, it should now be possible to embed photocatalytic sites within structurally diverse protein scaffolds. In this way, we can anticipate the development of selective photoenzymes for a broad range of thermally forbidden chemistries, including those that are challenging to achieve with existing small molecule photocatalysts.

## Supporting Information

Materials and methods, Figures S1–S16 and Tables S1–S12. Coordinates and structure factors for the X‐ray crystal structure of CEnT1.4 have been deposited in the Protein Data Bank under accession numbers 9ENO (https://www.rcsb.org/structure/unreleased/9ENO [rcsb.org]).

## Conflict of Interests

The authors declare no conflict of interest.

## Supporting information



Supporting Information

## Data Availability

The data that support the findings of this study are available in the Supporting Information of this article.
