# Supplementary material for: Photosensitizer Repositioning Affords an Enantiocomplementary Enzyme for [2 + 2]‐Cycloadditions
Source: Angew Chem Int Ed Engl. 2025 Jul 4;64(35):e202503576. doi: 10.1002/anie.202503576 (PMC12377449; doi:10.1002/anie.202503576)
Supplement: Supplementary file 1 — Supporting Information [file ANIE-64-e202503576-s001.pdf]

Supporting Information

Photosensitizer Repositioning Affords an Enantiocomplementary Enzyme for [2+2]-Cycloadditions

Chuanjie Sun,[1†] Anna R. Kohn,[1†] Ross Smithson,[1†] Florence J. Hardy,[1] Jonathan S. Trimble,[1] Yuanxin Cao,[1] Linus O. Johannissen,[1] Ross Smithson,[1] Sam Hay,[1*] Rebecca Crawshaw,[1*] and Anthony P. Green,[1*]

[*] Corresponding author

[†] These authors contributed equally

[1] Department of Chemistry & Manchester Institute of Biotechnology, The University of Manchester, 131 Princess Street, Manchester, M1 7DN, U.K.

Table of Contents

Materials and Methods *3*

Chemical Procedures *9*

General procedure 1: synthesis of 4-(allyloxy)-quinolones (4-9) *9*

General procedure 2: synthesis of intramolecular [2+2] cycloaddition product standards (4a-9a) *9*

Supporting Figures *16*

Supporting Tables *31*

Protein and DNA sequences *47*

NMR Spectra *51*

Substrate Scope Chromatograms *74*

Materials and Methods

Substrate **1** and products **1a** and **1b** were chemically synthesised according to procedures previously reported.[1] All other chemicals and biological materials were obtained from commercial suppliers. Lysozyme, DNase I, chloramphenicol and kanamycin were purchased from Sigma-Aldrich. Polymyxin B sulfate was obtained from Alfa Aesar. LB agar, LB media, 2x YT media, isopropyl-β-d-1-thiogalactopyranoside (IPTG) and arabinose were purchased from Formedium. (*S*)-2-amino-3-(4-benzoylphenyl)propanoic acid was obtained from Fluorochem. The pEVOL_pBpA/tRNA$_{CUA}$ plasmid[2] was purchased from Addgene. *Escherichia coli* strains BL21(DE3) and 5α, Q5 DNA polymerase, T4 DNA ligase and restriction enzymes were purchased from New England Biolabs. Oligonucleotides and genes were synthesized by Integrated DNA Technologies (IDT). Irradiation times given below refer to the total length of light exposure.

Construction of pET-29b_CEnT1.0 and variants

The original DA_20_00 computational design[3] was subcloned using *NdeI* and *XhoI* restriction sites into a pET-29b(+) vector containing a N-terminal His$_6$-tag to yield pET-29b(+)_DA_20_00. The Trp244BpA mutation was introduced by replacing the Trp244 codon with a TAG stop codon using QuikChange site-directed mutagenesis (Agilent) to yield pET-29b(+)_CEnT1.0. Point mutants of CEnT1.0 and variants were constructed using the same procedure.

Protein production and purification

For expression of CEnT1.0 and its variants, chemically competent *E. coli* BL21(DE3) cells containing pEVOL_pBpA/tRNA$_{CUA}$[3] were transformed with the appropriate pET-29b(+) construct. A single colony of freshly transformed cells was used to inoculate 5 mL LB medium containing 50 µg mL^{-1} kanamycin and 25 µg mL^{-1} chloramphenicol and cultured for 18 h at 37 °C and 200 r.p.m. Starter cultures (500 µL) were used to inoculate 50 mL 2x YT medium supplemented with 50 µg mL^{-1} kanamycin, 25 µg mL^{-1} chloramphenicol and 1 mM (*S*)-2-amino-3-(4-benzoylphenyl)propanoic acid (BpA), which was added as a 1 M stock dissolved in 1 M NaOH. Cultures were grown at 37 °C, 200 r.p.m. to an optical density at 600 nm (OD_{600}) of about 0.6 A.U. Protein expression was induced with the addition of L-arabinose to a final concentration of 0.05% and IPTG to a final concentration of 0.1 mM. Induced cultures were incubated for 20 h at 25 °C and the cells were subsequently collected by centrifugation (3,220 × g for 10 min). Pelleted cells were resuspended in lysis buffer (50 mM HEPES, 300 mM NaCl, 20 mM imidazole, pH 7.5) and lysed by sonication (1 s on/off, 5 min total sonication) at 4 °C with the addition of 1 µg mL^{-1} DNase I. Cell lysates were clarified by centrifugation (27,216 × g for 30 min) and supernatants were subjected to affinity chromatography using Ni-NTA Agarose (QIAGEN). His$_6$-tagged proteins were eluted using 50 mM HEPES, 300 mM NaCl, 50 mM imidazole, pH 7.5. Buffer exchange of purified proteins into phosphate-buffered saline (PBS; 137 mM NaCl; 2.7 mM KCl; 10 mM Na_2HPO_4; 1.8 mM KH_2PO_4, pH 7.4) was performed using 10DG desalting columns (Bio-Rad). Purified proteins were analysed by sodium dodecyl sulfate–polyacrylamide gel electrophoresis (SDS-PAGE). Proteins were aliquoted, flash-frozen in liquid nitrogen and stored at −80 °C. Protein

concentrations were determined by NanoDrop using an extinction coefficient of 62,152 M^{-1} cm^{-1} for EnT1.3 and variants, and 51,280 M^{-1} cm^{-1} for CEnT1.0 and variants. Extinction coefficients for variants containing BpA were deduced through a comparison of DA_20_00 and CEnT1.0 using a BCA Protein Assay kit (Thermo Fisher).

Mass spectrometry
Purified protein samples were desalted using 10,000 MWCO Vivaspin centrifugal concentrators (Sartorius) using 0.1% acetic acid and diluted to a final concentration of 0.4 mg mL^{-1}. Mass spectrometry was performed on a 1200 Series Agilent LC in conjunction with a Agilent 6510 QTOF. A 5 µl sample injection was performed, followed by a 1 min isocratic wash of 5% acetonitrile (with 0.1% formic acid). Proteins were eluted over 1 min using 95% acetonitrile with 5% water. The resulting multiply charged spectrum was deconvoluted using Agilent MassHunter Software. Protein mass spectrometry results are shown in Table S5.

Library construction
Rounds 1-4- site saturation mutagenesis: Amino acid positions were individually randomized using degenerate NNK codons. DNA libraries were constructed by overlap extension polymerase chain reaction (PCR). Primers for library generation are given in Table S6. Assembled genes and pET-29b(+) vector were digested using *NdeI* and *XhoI* endonucleases, gel-purified and subsequently ligated using T4 DNA ligase in a 5:1 ratio, respectively. Ligations were transformed into chemically competent *E. coli* 5α cells and the resulting colonies were pooled for each library. Plasmid DNA was extracted from each library pool using a Miniprep Kit (QIAGEN). Sequencing of library pools was performed by Source Bioscience (Nottingham).

Shuffling by overlap extension PCR
After evolution rounds 1-4, beneficial mutations were combined by DNA shuffling of fragments generated by overlap extension PCR. Primers were designed that encoded either the parent amino acid or the identified mutation. These primers were used to generate short fragments that were gel-purified and mixed for assembly of the full-length gene by overlap extension PCR. The final full-length genes contain all possible combinations of mutations at specified positions. Genes were cloned as described above.

Library screening
For protein expression and screening, all transfer and aliquoting steps were performed using Hamilton liquid-handling robots. Chemically competent *E. coli* BL21(DE3) cells harbouring pEVOL_pBpA/$tRNA_{CUA}$[1] were transformed with the appropriate library plasmids. Resulting colonies were used to inoculate 150 µL of 2x YT medium supplemented with 50 µg mL^{-1} kanamycin and 25 µg mL^{-1} chloramphenicol in Corning Costar 96-well microtitre round-bottom plates. Each plate contained six freshly transformed clones of the parent template and two clones containing pET-29b(+)_CEnt1.0 as internal references. Plates were incubated overnight at 30 °C, 80% humidity in a shaking incubator at 900 r.p.m. 20 µL of overnight culture was used to inoculate 480 µL of 2x YT medium supplemented with

50 µg mL^{-1} kanamycin, 25 µg mL^{-1} chloramphenicol and 1 mM BpA, which was added as a 1 M stock dissolved in 1 M NaOH. The cultures were incubated at 30 °C, 80% humidity and 900 r.p.m. until an OD_{600} of about 0.6 A.U. Protein expression was induced by the addition of L-arabinose to a final concentration of 0.05% and IPTG to a final concentration of 0.1 mM. Induced plates were incubated for 20 h at 30 °C, 80% humidity and 900 r.p.m. Cells were harvested by centrifugation at 2,900 × g for 5 min. The supernatant was discarded and the pelleted cells were resuspended in 400 µL of PBS lysis buffer (137 mM NaCl; 2.7 mM KCl; 10 mM Na_2HPO_4; 1.8 mM KH_2PO_4, pH 7.4, 1.0 mg mL^{-1} lysozyme, 0.5 mg mL^{-1} polymyxin B and 1 µg mL^{-1} DNase I) and incubated for 2 h at 30 °C, 80% humidity, with shaking at 900 r.p.m.. Cell debris was removed by centrifugation at 2,900 × g for 5 min.

Rounds 1 and 2

75 µL of clarified lysate was transferred to 96-well polypropylene microtitre plates containing 25 µL of 1.2 mM substrate **1** in PBS buffer (pH 7.4) with 5% dimethyl sulfoxide (DMSO) as a cosolvent. Microtitre plates were sealed with HD Clear high-performance tape (Duck). Samples were irradiated at 365 nm in an ultraviolet (UV) curing LED oven (equipped with 365-nm and 395-nm LEDs, UV intensity 750 mW cm^{-2}, LED module size 100 × 100, NovaChem), with pulsing irradiation (10 s on, 10 s off) at 4 °C for a total irradiation time of 15 min at 100% intensity. Reactions were quenched with the addition of 100 µL of acetonitrile. The plates were heat-sealed and incubated for a further 1 h at 30 °C and 900 r.p.m. Precipitated proteins were removed by centrifugation at 2,900 × g for 10 min. A 100 µL volume of the clarified reaction mixture was transferred to 96-well polypropylene microtitre plates and heat-sealed with pierceable foil. Reactions were evaluated by UPLC analysis.

Round 3

75 µL of clarified lysate was transferred to 96-well polypropylene microtitre plates containing 25 µL of 1.2 mM substrate **1** in PBS buffer (pH 7.4) with 5% dimethyl sulfoxide (DMSO) as a cosolvent. Microtitre plates were sealed with HD Clear high-performance tape (Duck). Samples were irradiated at 365 nm in an ultraviolet (UV) curing LED oven (equipped with 365-nm and 395-nm LEDs, UV intensity 750 mW cm^{-2}, LED module size 100 × 100, NovaChem), with pulsing irradiation (10 s on, 10 s off) at 4 °C for a total irradiation time of 5 min at 100% intensity. Reactions were quenched with the addition of 100 µL of acetonitrile. The plates were heat-sealed and incubated for a further 1 h at 30 °C and 900 r.p.m. Precipitated proteins were removed by centrifugation at 2,900 × g for 10 min. A 100 µL volume of the clarified reaction mixture was transferred to 96-well polypropylene microtitre plates and heat-sealed with pierceable foil. Reactions were evaluated by UPLC analysis.

Round 4

75 µL of clarified lysate was transferred to 96-well polypropylene microtitre plates containing 25 µL of 1.2 mM substrate **2** in PBS buffer (pH 7.4) with 5% dimethyl sulfoxide (DMSO) as a cosolvent. Microtitre plates were sealed with HD Clear high-performance tape (Duck). Samples were irradiated at 365 nm in an ultraviolet (UV) curing LED oven (equipped with 365-nm and 395-nm LEDs, UV intensity 750 mW cm^{-2}, LED module size 100 × 100,

NovaChem), with pulsing irradiation (10 s on, 10 s off) at 4 °C for a total irradiation time of 20 min at 50% intensity. Reactions were quenched with the addition of 100 µL of acetonitrile. The plates were heat-sealed and incubated for a further 1 h at 30 °C and 900 r.p.m. Precipitated proteins were removed by centrifugation at 2,900 × g for 10 min. A 100 µL volume of the clarified reaction mixture was transferred to 96-well polypropylene microtitre plates and heat-sealed with pierceable foil. Reactions were evaluated by UPLC analysis.

General procedure for analytical-scale biotransformations

96-well microtitre round-bottom polypropylene plates were used for biotransformations, using HD Clear high-performance tape (Duck), 3-inch × 54.6-yard roll, to seal the samples. Biotransformations were performed at 4 °C using 0.4 mM substrate **1** and the relevant biocatalyst (20 µM) in PBS buffer pH 7.4 with 5% DMSO as a cosolvent. Samples were irradiated at 365 nm in a UV curing LED oven (NovaChem), 23 cm below the LED array (unless stated otherwise). Instrument settings: 100% intensity, 1500 mW cm^{-2}, 10 s on/off pulse. Reactions were evaluated by UPLC analysis. Conditions for substrate scope characterisation are detailed in Supporting Information, Table S12. Reactions were evaluated by UPLC analysis.

Anaerobic biotransformations

For anaerobic biotransformations, samples of CEnT1.4 and substrate were incubated in a glovebox overnight on ice to ensure complete removal of oxygen. Reactions were made up in the glovebox in screw cap glass vials (final volume of 200 µL) using 2.5 mol% (10 µM) CEnT1.4 in PBS (pH 7.4) with 5% DMSO as a cosolvent. Vials in triplicate were taken out of the light source at 10, 20, 30, 60 and 90 min and quenched with one volume of MeCN. Reactions were evaluated by UPLC analysis.

Total turnover numbers

Total turnover numbers achieved by CEnT1.4 were determined as follows. CEnT1.4 (0.125 mol%)-catalysed biotransformations were performed in glass vials using **1** (800 µM) in PBS (pH 7.4) with 10% DMSO cosolvent in a 1 mL volume. Reactions were performed under standard conditions and 50 µL samples were taken at 10, 20, 30, 40, 50, 60, 90, and 120 min. Reactions were quenched with one reaction volume of MeCN and evaluated by UPLC analysis.

CEnT1.4 temperature profile

Biotransformations were performed at 4 °C and room temperature in glass vials using 20 µM of CEnT1.4 and 400 µM of substrate **1** in PBS buffer with 5% DMSO as a cosolvent (final volume of 500 µL). For reactions at 4 °C, all reaction components were incubated in a cold room maintained at 4 °C for 30 min before running reactions. For both temperatures, reactions were run using 100% intensity irradiation at 365 nm with 10 s on/10 s off intervals and 25 µL samples were taken at 10, 20, 30, 60 and 90 min. Reactions were evaluated by UPLC analysis.

CEnT1.4 substrate concentration rate profile

To investigate the effect of substrate concentration on the reaction rate of CEnT1.4, biotransformations were performed using 1 µM CEnT1.4 and a range of substrate

concentrations (0 µM, 20 µM, 35 µM, 50 µM, 80 µM, 100 µM, 125 µM, 150 µM, 200 µM, 250 µM and 300 µM) in PBS buffer (pH 7.4) with 5% DMSO as a cosolvent. Reactions were run in a 96-well plate with a volume of 250 µL and irradiated at 4 °C (10 s on/off pulse at 365 nm). Time points were taken at 4 min, 8 min, 12 min and 16 min of irradiation by sampling 50 µL of the reaction and quenching with one volume of MeCN. Samples were analysed by UPLC analysis. The initial rate v_0 at each substrate concentration was calculated in triplicate using the slope of the time course at less than 10% conversion.

CEnT1.4 light intensity rate profile

To investigate the effect of light intensity on the reaction rate of CEnT1.4, biotransformations were performed in 2 mL MS glass vials using 1 µM CEnT1.4 and 400 µM **1** in PBS buffer (pH 7.4) with 5% DMSO as a cosolvent (500 µL total reaction volume). Reactions were positioned at a 3 cm distance from the LED array and irradiated as described at varying LED intensities. Time points were taken at 1 min, 2 min, 3 min, 4 min and 5 min for 100% and 50%, and 2 min, 4 min, 6 min, 8 min and 10 min for 30% and 40%, and 4 min, 8 min, 12 min, 16 min and 20 min for 10% and 20% and analysed by UPLC analysis. The reaction rate at each light intensity was calculated using the slope of the time course (as an average of triplicate measurements), which remained linear in each case.

Preparative-scale biotransformation (substrate 1)

Substrate **1** (11.4 mg) was dissolved to a concentration of 400 µM in PBS buffer (pH 7.4) with 5% DMSO as a cosolvent (133 mL reaction volume) with 20 µM CEnT1.4 (5 mol%). The solution was irradiated as described at 365 nm for a total reaction time of 1.5 h in a Pyrex dish (22 cm diameter, solution path length 0.37 cm). Upon reaction completion (as monitored by UPLC, 98% conversion), the solution was transferred to a separatory funnel and extracted with 3 × 20 mL ethyl acetate and the combined organic layers were washed with 3 × 20 mL brine, dried over $MgSO_4$, filtered and concentrated *in vacuo* to afford optically pure **1a** (11 mg, 96% yield) with a minor carryover of DMSO, which was further purified through flash-chromatography to afford optically pure **1a** (8.0 mg, 70% isolated yield).

Preparative-scale biotransformation (substrate 7)

Substrate **7** (10 mg) was dissolved to a concentration of 400 µM in PBS buffer (pH 7.4) with 20% DMSO as a cosolvent (85 mL reaction volume) with 50 µM CEnT1.3 C271V A272G Y121A (12.5 mol%). The solution was irradiated as described at 365 nm for a total reaction time of 25 minutes in a Pyrex dish (22 cm diameter, solution path length 0.37 cm). Upon reaction completion (as monitored by UPLC, >99% conversion), the solution was transferred to a separatory funnel and extracted with 3 × 20 mL ethyl acetate and the combined organic layers were washed with 3 × 20 mL brine, dried over $MgSO_4$, filtered and concentrated *in vacuo* to afford **7a** with a minor impurity of **7b** (9 mg, 90% yield). This was further purified through flash-chromatography to afford **7a** (7.0 mg, 70% isolated yield). The isolated product was analysed by chiral UPLC (IB N-3, 12:88 IPA:Hexane) affording 84% e.e. (major= 3.6 min; minor= 4.2 min).

Reductive dehalogenation of a preparative-scale biotransformation (substrate 7)

To confirm the absolute stereochemistry of product **7a**, the isolated product from a biotransformation with CEnT1.3 C271V A272G Y121A was subjected to reductive dehalogenation to produce (+)-**1a** (83% e.e., see Chemical Procedures). Chiral UPLC analysis confirms that the major enantiomer of **1a** formed following the reductive dehalogenation of **7a** is the same as that formed from enzymatic conversion of substrate **1** to **1a** (Supplementary Figures S8 and S9). Chiral UPLC (IB N-3, 12:88 IPA:Hexane) affording 83% e.e. (minor: (-)**1a**=3.7 min; major: (+)**1a**= 7.5 min).

Chromatographic analysis

For UPLC analysis, reactions were quenched at the stated time points with the addition of one volume of MeCN. Samples were shaken at 900 r.p.m for 1 h and precipitated proteins were removed by centrifugation (2,900 × g for 10 min). For SFC analysis, the substrates and products were transferred into 1.5 mL microcentrifuge tubes and extracted with three volumes of ethyl acetate. Precipitated proteins were removed by centrifugation (14,000 × g for 15 min), the organic phase was separated and directly injected onto the SFC.

UPLC analysis was performed on a 1290 Infinity II LC system (Agilent) with a Kinetex 5 µm XB-C18 100 Å LC Column, 50 × 2.1 mm (Phenomenex). Peaks were assigned by comparison with chemically synthesized standards and the peak areas were integrated using Agilent's OpenLab software. The achiral separation methods for reactions performed with substrate **1** and the substrate scope are shown in Table S10.

Chiral analysis was performed using a SFC 1290 Infinity II system (Agilent). Enantiomers of all reaction products **1a** were separated using a Daicel 87S82 CHIRALPAK IG-3 SFC column, 3 mm × 50 mm × 3 µm. Peaks were assigned by comparison with chemically synthesized standards and peak areas were integrated using Agilent's OpenLab software. The chiral separation methods for reactions performed with substrate **1** and the substrate scope are shown in Table S11 and example traces are given in at the end of the Supporting Information.

Chemical Procedures

Substrates 1, 2 and 3 along with their product standards were synthesised as previously reported[1].

General procedure 1: synthesis of 4-(allyloxy)-quinolones (4-9)

Quinoline-2,4-diol (250 mg, 1.55 mmol, 1 eq.) was dissolved in 5 mL of dry dimethylformamide with NaH (55.8 mg, 2.33 mmol, 1.5 eq.) and stirred at 90 °C for 1 h under nitrogen. Subsequently, the 1-bromobut-1-ene (2.33 mmol, 1.5 eq.) was added dropwise and the solution was stirred overnight at 90 °C. The reaction was quenched by addition of aqueous 1M HCl (15 mL) and then extracted with ethyl acetate (3 × 100 mL). The combined organic layers were washed with brine (2 × 50 mL), dried over $MgSO_4$, filtered and the solvent removed *in vacuo* to give the crude product.

General procedure 2: synthesis of intramolecular [2+2] cycloaddition product standards (4a-9a)

The synthesised quinolones (**4-9**) were dissolved in acetonitrile to a concentration of 10 mM and degassed by bubbling nitrogen through the solution for 30 min. The solution was irradiated at $\lambda = 300$ nm at room temperature until full conversion was achieved (as monitored by thin-layer chromatography). The solvent was removed *in vacuo* to give the crude product.

4-(but-3-en-1-yloxy)-6-methylquinolin-2(1H)-one (4)

The crude product was purified by flash chromatography (1:2 ethyl acetate:cyclohexane) to give the product as a white solid (97 mg, 27.3 %). HR-MS (ESI) *m/z* = found $[M+H]^+$ 230.1214; calc.: 229.1103. ^{1}H NMR (400 MHz, $CDCl_3$, 298K), δ [ppm] = 11.00 (s, 1H, *NH*), 7.68 (s, 1H, *Ph*), 7.32 (d, *J* = 8.2 Hz, 1H, *Ph*), 7.20 (d, *J* = 8.3 Hz, 1H), 5.99 – 5.90 (m, 2H, *CH*, *CH*), 5.24 (dt, *J* = 17.1, 1.7 Hz, 1H, *CH*), 5.17 (d, *J* = 10.3 Hz, 1H, *CH*), 4.16 (t, *J* = 6.5 Hz, 2H, CH_2), 2.68 (q, *J* = 6.5 Hz, 2H, CH_2), 2.41 (s, 3H, CH_3). ^{13}C NMR (101 MHz, DMSO-d6, 298K), δ [ppm] = 163.15, 162.10, 136.67, 134.70, 132.14, 130.27, 121.66, 117.30, 115.16, 114.47, 97.07, 67.33, 32.61, 20.60.

4-(but-3-en-1-yloxy)-6-fluoroquinolin-2(1H)-one (5)

The crude product was purified by flash chromatography (1:1 ethyl acetate:cyclohexane) to give the product as a white solid (82 mg, 22.7 %). HR-MS (ESI) *m/z* = found $[M+H]^+$ 234.0849; calc.: 233.0852. ^{1}H NMR (400 MHz, $CDCl_3$, 298K), *δ* [ppm] = ^{1}H NMR (400 MHz, $CDCl_3$) δ 12.00 (s, 1H, *NH*), 7.56 (dd, *J* = 9.1, 2.8 Hz, 1H, *Ph*), 7.35 (dd, *J* = 9.0, 4.6 Hz, 1H, *Ph*), 7.26 – 7.22 (m, 1H, *Ph*), 6.02 (s, 1H, *CH*), 5.93 (ddt, *J* = 17.0, 10.3, 6.7 Hz, 1H, *CH*), 5.24 (dq, *J* = 17.2, 1.6 Hz, 1H, *CH*), 5.18 (dt, *J* = 10.2, 1.4 Hz, 1H, *CH*), 4.17 (t, *J* = 6.5 Hz, 2H, CH_2), 2.71 – 2.63 (m, 2H, CH_2). ^{13}C NMR (101 MHz, $CDCl_3$, 298K), δ [ppm] = 164.40, 163.27, 149.49 (d, *J* = 245.1 Hz), 133.57, 127.42 (d, *J* = 14.1 Hz), 121.52 (d, *J* = 7.0 Hz), 118.50 (d, *J* = 3.7 Hz), 117.84, 117.61 (d, *J* = 3.1 Hz), 116.01 (d, *J* = 17.2 Hz), 97.66, 67.98, 33.01. ^{19}F NMR (376 MHz, $CDCl_3$, 298K), δ [ppm] = -133.65

4-(but-3-en-1-yloxy)-6-chloroquinolin-2(1H)-one (6)

The crude product was purified by flash chromatography (1:2 ethyl acetate:cyclohexane) to give the product as a white solid (89 mg, 23.0 %). HR-MS (ESI) *m/z* = found $[M+H]^+$ 250.0581; calc.: 249.0057. ^{1}H NMR (500 MHz, $CDCl_3$, 298K), *δ* [ppm] = 11.95 (s, 1H, *NH*), 7.87 (d, *J* = 2.3 Hz, 1H, *Ph*), 7.47 (dd, *J* = 8.7, 2.4 Hz, 1H, *Ph*), 7.33 (d, *J* = 8.7 Hz, 1H, *Ph*),

6.02 (s, 1H, *CH*), 5.93 (ddt, *J* = 17.0, 10.3, 6.7 Hz, 1H, *CH*), 5.25 (dq, *J* = 17.1, 1.6 Hz, 1H, *CH*), 5.19 (dq, *J* = 10.3, 1.4 Hz, 1H, *CH*), 4.18 (t, *J* = 6.6 Hz, 2H, CH_2), 2.68 (q, *J* = 6.6 Hz, 2H, CH_2). ^{13}C NMR (101 MHz, DMSO-d6) δ [ppm] = 163.03, 161.10, 137.40, 134.70, 130.94, 125.42, 121.32, 117.30, 117.23, 115.79, 98.12, 67.60, 32.53.

6-bromo-4-(but-3-en-1-yloxy)quinolin-2(1H)-one (7)

The crude product was purified by flash chromatography (1:1 ethyl acetate:cyclohexane) to give the product as a white solid (104 mg, 22.9 %). HR-MS (ESI) *m/z* = found $[M+H]^+$ 294.0328; calc.: 293.0051. ^{1}H NMR (400 MHz, $CDCl_3$, 298K), *δ* [ppm] = 11.71 (s, 1H, *NH*), 8.02 (d, *J* = 2.2 Hz, 1H, *Ph*), 7.58 (dd, *J* = 8.7, 2.2 Hz, 1H, *Ph*), 7.23 (d, *J* = 8.7 Hz, 1H, *Ph*), 5.98 (s, 1H, *CH*), 5.93 (ddt, *J* = 17.0, 10.3, 6.7 Hz, 1H, *CH*), 5.24 (dd, *J* = 17.2, 1.7 Hz, 1H, *CH*), 5.19 (d, *J* = 10.3 Hz, 1H, *CH*), 4.16 (t, *J* = 6.6 Hz, 2H, CH_2), 2.68 (q, *J* = 6.6 Hz, 2H, CH_2). ^{13}C NMR (126 MHz, $CDCl_3$, 298K) δ [ppm] = 165.71, 163.47, 137.06, 134.47, 133.47, 125.69, 118.16, 117.99, 117.35, 115.59, 96.95, 68.42, 33.08.

4-(but-3-en-1-yloxy)-8-fluoroquinolin-2(1H)-one (8)

The crude product was purified by flash chromatography (1:1 ethyl acetate:cyclohexane) to give the product as a white solid (123 mg, 34.0 %). HR-MS (ESI) *m/z* = found $[M+H]^+$ 234.0940; calc.: 233.0852. ^{1}H NMR (500 MHz, $CDCl_3$, 298K), *δ* [ppm] = 9.95 (s, 1H, *NH*), 7.68 (d, *J* = 8.1 Hz, 1H, *Ph*), 7.28 (m, 1H, *Ph*), 7.11 (td, *J* = 8.1, 5.0 Hz, 1H, *Ph*), 6.00 (s, 1H, *CH*), 5.92 (ddt, *J* = 17.0, 10.3, 6.7 Hz, 1H, *CH*), 5.23 (dq, *J* = 17.1, 1.5 Hz, 1H, *CH*), 5.17 (dd, *J* = 10.3, 1.5 Hz, 1H, *CH*), 4.16 (t, *J* = 6.5 Hz, 2H, CH_2), 2.66 (q, *J* = 6.6 Hz, 2H, CH_2). ^{13}C NMR (126 MHz, $CDCl_3$, 298K), δ [ppm] = 164.26, 163.47 (d, *J* = 3.5 Hz), 149.55 (d, *J* = 245.2 Hz), 133.67, 127.45 (d, *J* = 14.1 Hz), 121.72 (d, *J* = 7.1 Hz), 118.67 (d, *J* = 3.9 Hz), 117.99, 117.74 (d, *J* = 2.9 Hz), 116.16 (d, *J* = 17.1 Hz), 97.71, 68.14, 33.12. ^{19}F NMR (471 MHz, $CDCl_3$, 298K) δ [ppm] = -133.99.

4-(but-3-en-1-yloxy)-7-methoxyquinolin-2(1H)-one (9)

The crude product was purified by flash chromatography (100% ethyl acetate) to give the product as a white solid (153 mg, 40.2 %). HR-MS (ESI) *m/z* = found $[M+H]^+$ 246.1098; calc.: 245.1052. ^{1}H NMR (400 MHz, $CDCl_3$, 298K), δ [ppm] = 12.04 (s, 1H, *NH*), 7.79 (d, *J* = 8.4 Hz, 1H, *Ph*), 6.77 (m, 2H, *Ph*), 5.93 (ddt, *J* = 17.0, 10.3, 6.7 Hz, 1H, *CH*), 5.87 (s, 1H, *CH*), 5.22 (d, *J* = 17.2 Hz, 1H, CH_2), 5.16 (d, *J* = 10.3 Hz, 1H, CH_2), 4.15 (t, *J* = 6.5 Hz, 2H, CH_2), 3.89 (s, 3H, CH_3), 2.65 (q, *J* = 6.5 Hz, 2H, CH_2). ^{13}C NMR (101 MHz, $CDCl_3$, 298K), δ [ppm] = 166.47, 164.06, 162.07, 140.14, 133.62, 124.03, 117.46, 111.55, 109.28, 97.92, 93.93, 67.44, 55.47, 32.94.

Products

9-methyl-3,3a,4,4a-tetrahydro-2H-furo[2',3':2,3]cyclobuta[1,2-c]quinolin-5(6H)-one (4a, straight product)
The crude product was purified by flash chromatography (2:1 ethyl acetate:cyclohexane) to give the product as a white solid (12 mg, 24 %). HR-MS (ESI) *m/z* = found $[M+H]^+$ 230.1207; calc.: 229.1103. ^1H NMR (400 MHz, $CDCl_3$, 298K) δ [ppm] = 8.80 (s, 1H, *NH*), 7.07 (s, 1H, *Ph*), 7.00 (d, *J* = 8.1 Hz, 1H, *Ph*), 6.71 (d, *J* = 7.9 Hz, 1H, *Ph*), 4.45 (t, *J* = 8.5 Hz, 1H, *CH*), 4.22 (td, *J* = 10.1, 5.6 Hz, 1H, *CH*), 3.33 (dd, *J* = 11.4, 7.0 Hz, 1H, *CH*), 2.98 – 2.90 (m, 1H, *CH*), 2.43 – 2.36 (m, 1H, *CH*), 2.27 (s, 3H, CH_3), 2.22 – 2.12 (m, 2H, CH_2), 1.83 (dd, *J* = 12.7, 5.5 Hz, 1H, *CH*). ^{13}C NMR (101 MHz, $CDCl_3$, 298K), δ [ppm] = 172.13, 133.81, 133.55, 130.00, 126.70, 123.30, 115.71, 83.53, 68.63, 46.75, 42.40, 32.47, 26.77, 20.93.

9-methyl-2,3,4,4a-tetrahydro-4,10b-methanopyrano[3,2-c]quinolin-5(6H)-one (4, crossed product)
The crude product was purified by flash chromatography (1:1 ethyl acetate:cyclohexane) to give the product as a white solid (8 mg, 16 %). HR-MS (ESI) *m/z* = found $[M+H]^+$ 230.1456; calc.: 229.1103. ^1H NMR (400 MHz, $CDCl_3$, 298K) δ [ppm] = 7.98 (s, 1H, *NH*), 7.21 (s, 1H, *Ph*), 7.00 (d, *J* = 7.7 Hz, 1H, *Ph*), 6.64 (d, *J* = 7.9 Hz, 1H, *Ph*), 4.44 – 4.37 (m, 1H, *CH*), 4.29-4.22 (m, 1H, *CH*), 3.12 – 3.07 (m, 1H, *CH*), 2.87 (d, *J* = 8.1 Hz, 1H, *CH*), 2.54 (dd, *J* = 10.9, 5.7 Hz, 1H, *CH*), 2.43-2.33 (m, 1H, *CH*), 2.30 (s, 3H, CH_3), 2.25 (m, 1H, *CH*), 2.17 (dd, *J* = 10.8, 7.8 Hz, 1H, *CH*). ^{13}C NMR (126 MHz, $CDCl_3$, 298K), δ [ppm] = 172.21, 133.57, 132.88, 129.29, 125.10, 124.56, 115.23, 80.27, 60.97, 51.20, 36.86, 36.64, 31.69, 21.00.

9-fluoro-3,3a,4,4a-tetrahydro-2H-furo[2',3':2,3]cyclobuta[1,2-c]quinolin-5(6H)-one (5a, straight product)
The crude product was purified by flash chromatography (2:1 ethyl acetate:cyclohexane) to give the product as a white solid (15 mg, 30 %). HR-MS (ESI) *m/z* = found $[M+H]^+$ 234.0983; calc.: 233.0852. ^1H NMR (400 MHz, $CDCl_3$, 298K) δ [ppm] = 9.87 (s, 1H, *NH*), 6.97 (d, *J* = 8.9 Hz, 1H, *Ph*), 6.93 – 6.86 (m, 1H, *Ph*), 6.81 (dd, *J* = 8.7, 4.7 Hz, 1H, *Ph*), 4.45 (t, *J* = 8.6 Hz, 1H, *CH*), 4.27-4.17 (m, 1H, *CH*), 3.32 (dd, *J* = 11.4, 6.6 Hz, 1H, *CH*), 2.98 (q, *J* = 8.3 Hz, 1H, *CH*), 2.20 (td, *J* = 12.3, 5.3 Hz, 1H, *CH*), 2.15 – 2.06 (m, 1H, *CH*), 1.83 (dd, *J* = 12.7, 5.6 Hz, 1H, *CH*). ^{13}C NMR (101 MHz, $CDCl_3$, 298K) δ [ppm] = 172.42, 159.40 (d, *J* = 242.6 Hz), 132.35 (d, *J* = 2.5 Hz), 125.36 (d, *J* = 6.8 Hz), 117.12 (d, *J* = 7.8 Hz), 116.20 (d, *J* = 23.1 Hz), 112.94 (d, *J* = 23.6 Hz), 83.28, 68.84, 46.91, 41.97, 32.29, 26.91. ^{19}F NMR (376 MHz, $CDCl_3$, 298K) δ -118.95.

9-fluoro-2,3,4,4a-tetrahydro-4,10b-methanopyrano[3,2-c]quinolin-5(6H)-one (5, crossed product)
The crude product was purified by flash chromatography (2:1 ethyl acetate:cyclohexane) to give the product as a white solid (6 mg, 12 %). HR-MS (ESI) *m/z* = found $[M+H]^+$ 234.1195; calc.: 233.0852. ^1H NMR (400 MHz, $CDCl_3$, 298K) δ [ppm] = 8.81 (bs, 1H, *NH*), 7.11 (dd, *J* = 8.7, 3.1 Hz, 1H, *Ph*), 6.90 (td, *J* = 8.3, 3.2 Hz, 1H, *Ph*), 6.78 (dd, *J* = 8.7, 4.6 Hz, 1H, *Ph*), 4.44-4.36 (m, 1H, *CH*), 4.29-4.21 (m, 1H, *CH*), 3.12 (s, 1H, *CH*), 2.86 (d, *J* = 8.1 Hz, 1H, *CH*), 2.57 (dd, *J* = 11.1, 5.8 Hz, 1H, *CH*), 2.44-2.34 (m, 1H, *CH*), 2.30-2.22 (m, 1H, *CH*), 2.18 (dd, *J* = 11.0, 7.8 Hz, 1H). ^{13}C NMR (126 MHz, $CDCl_3$, 298K) δ [ppm] = 172.82, 159.73 (d, *J* = 242.8 Hz), 131.75 (d, *J* = 2.6 Hz), 127.29 (d, *J* = 7.3 Hz), 116.85 (d, *J* = 8.1 Hz), 115.62 (d, *J* = 23.3 Hz), 111.65 (d, *J* = 24.3 Hz), 80.16, 61.25, 51.10, 37.22, 36.61, 31.83. ^{19}F NMR (471 MHz, $CDCl_3$, 298K) δ [ppm] -118.83.

9-chloro-3,3a,4,4a-tetrahydro-2H-furo[2',3':2,3]cyclobuta[1,2-c]quinolin-5(6H)-one (6a, straight product)

The crude product was purified by flash chromatography (2:1 ethyl acetate:cyclohexane) to give the product as a white solid (11 mg, 22 %). HR-MS (ESI) *m/z* = found $[M+H]^+$ 250.0621; calc.: 249.0057. ^{1}H NMR (500 MHz, $CDCl_3$, 298K) δ [ppm] = 9.52 (bs, 1H, *NH*), 7.29 – 7.26 (m, 1H, *Ph*), 7.18 (dd, *J* = 8.6, 2.4 Hz, 1H, *Ph*), 6.79 (d, *J* = 8.6 Hz, 1H, *Ph*), 4.48 (t, *J* = 8.5 Hz, 1H, *CH*), 4.24 (td, *J* = 9.3, 5.5 Hz, 1H, *CH*), 3.35 (dd, *J* = 4.1 Hz, 1H, *CH*), 3.00 (q, *J* = 8.6 Hz, 1H, *CH*), 2.48-2.39 (m, 1H, *CH*), 2.22 (td, *J* = 12.2, 5.2 Hz, 1H, *CH*), 2.18 – 2.11 (m, 1H, *CH*), 1.86 (dd, *J* = 12.9, 5.5 Hz, 1H, *CH*). ^{13}C NMR (101 MHz, $CDCl_3$, 298K) δ [ppm] = 172.28, 134.78, 129.35, 128.95, 126.46, 125.33, 117.10, 83.16, 68.89, 46.94, 42.20, 32.31, 26.96.

9-chloro-2,3,4,4a-tetrahydro-4,10b-methanopyrano[3,2-c]quinolin-5(6H)-one (6, crossed product)

The crude product was purified by flash chromatography (2:1 ethyl acetate:cyclohexane) to give the product as a white solid (9 mg, 18 %). HR-MS (ESI) *m/z* = found $[M+H]^+$ 250.0652; calc.: 249.0057. ^{1}H NMR (500 MHz, $CDCl_3$, 298K) δ [ppm] = 8.50 (s, 1H, *NH*), 7.38 (d, *J* = 2.6 Hz, 1H, *Ph*), 7.16 (dd, *J* = 8.4, 2.4 Hz, 1H, *Ph*), 6.76 (d, *J* = 8.4 Hz, 1H, *Ph*), 4.40 (dt, *J* = 11.3, 7.6 Hz, 1H, *CH*), 4.26 (td, *J* = 7.9, 4.0 Hz, 1H, *CH*), 3.15-3.09 (m, 1H, *CH*), 2.86 (d, *J* = 8.1 Hz, 1H, *CH*), 2.55 (dd, *J* = 11.0, 5.8 Hz, 1H, *CH*), 2.44 – 2.35 (m, 1H, *CH*), 2.30 – 2.23 (m, 1H, *CH*), 2.19 (dd, *J* = 11.1, 8.0 Hz, 1H, *CH*). ^{13}C NMR (101 MHz, $CDCl_3$, 298K) δ [ppm] = 172.71, 133.98, 129.10, 128.73, 126.76, 124.47, 116.71, 79.82, 61.00, 51.04, 37.04, 36.38, 31.55.

9-bromo-3,3a,4,4a-tetrahydro-2H-furo[2',3':2,3]cyclobuta[1,2-c]quinolin-5(6H)-one (7a, straight product)

The crude product was purified by flash chromatography (2:1 ethyl acetate:cyclohexane) to give the product as a white solid (19 mg, 38 %). HR-MS (ESI) *m/z*: found $[M+H]^+$ 294.0091; calc.: 293.0051. ^{1}H NMR (400 MHz, $CDCl_3$, 298K) δ [ppm] = 8.61 (s, 1H, *NH*), 7.38 (s, 1H, *Ph*), 7.31 (dd, *J* = 8.4, 2.6 Hz, 1H, *Ph*), 6.69 (d, *J* = 8.4 Hz, 1H, *Ph*), 4.46 (t, *J* = 8.5 Hz, 1H, *CH*), 4.26-4.17 (m, 1H, *CH*), 3.32 (dd, *J* = 11.1, 6.6 Hz, 1H, *CH*), 3.03 – 2.91 (m, 1H, *CH*), 2.46-2.35 (m, 1H, *CH*), 2.24 – 2.09 (m, 2H, CH_2), 1.84 (dd, *J* = 12.8, 5.4 Hz, 1H, *CH*). ^{13}C NMR (126 MHz, $CDCl_3$, 298k) δ [ppm] = 172.09, 135.25, 132.23, 129.41, 125.70, 117.38, 116.32, 83.12, 68.90, 46.96, 42.28, 32.30, 26.96.

9-bromo-2,3,4,4a-tetrahydro-4,10b-methanopyrano[3,2-c]quinolin-5(6H)-one (7, crossed product)

The crude product was purified by flash chromatography (2:1 ethyl acetate:cyclohexane) to give the product as a white solid (9 mg, 18 %). HR-MS (ESI) *m/z*: found $[M+H]^+$ 294.0328; calc.: 293.0051. ^{1}H NMR (500 MHz, $CDCl_3$, 298K), δ [ppm] = 8.60 (s, 1H, *NH*), 7.53 (d, *J* = 2.3 Hz, 1H, *Ph*), 7.31 (dd, *J* = 8.4, 2.3 Hz, 1H, *Ph*), 6.68 (d, *J* = 8.4 Hz, 1H, *Ph*), 4.40 (dt, *J* = 11.5, 7.7 Hz, 1H, *CH*), 4.26 (ddd, *J* = 11.6, 8.0, 3.9 Hz, 1H, *CH*), 3.14 – 3.09 (m, 1H, *CH*), 2.86 (d, *J* = 8.1 Hz, 1H, *CH*), 2.54 (ddd, *J* = 11.0, 5.8, 1.6 Hz, 1H, *CH*), 2.42 – 2.35 (m, 1H, *CH*), 2.30 – 2.23 (m, 1H, *CH*), 2.19 (dd, *J* = 11.1, 8.0 Hz, 1H). ^{13}C NMR (126 MHz, $CDCl_3$, 298K), δ [ppm] = 172.26, 134.31, 131.54, 127.27, 126.95, 116.84, 116.38, 79.63, 60.88, 50.98, 36.91, 36.29, 31.41.

7-fluoro-3,3a,4,4a-tetrahydro-2H-furo[2',3':2,3]cyclobuta[1,2-c]quinolin-5(6H)-one (8a, straight product)

The crude product was purified by flash chromatography (2:1 ethyl acetate:cyclohexane) to give the product as a white solid (19 mg, 38 %). HR-MS (ESI) *m/z*: found $[M+H]^+$ 234.0928; calc.: 233.0852. ^{1}H NMR (500 MHz, $CDCl_3$, 298K) δ [ppm] = 7.72 (s, 1H, *NH*), 7.10 – 7.04 (m, 1H, *Ph*), 7.04 – 6.93 (m, 2H, *Ph*), 4.46 (t, *J* = 8.6 Hz, 1H, *Ph*), 4.28 – 4.18 (m, 1H, *CH*), 3.41 – 3.31 (m, 1H, *CH*), 3.03 – 2.92 (m, 1H, *CH*), 2.47 – 2.37 (m, 1H, *CH*), 2.23 – 2.16 (m, 1H, *CH*), 2.16 – 2.08 (m, 1H, *CH*), 1.89 – 1.80 (m, 1H, *CH*). ^{13}C NMR (126 MHz, $CDCl_3$, 298K), δ [ppm] = 170.32, 149.30 (d, *J* = 243.2 Hz), 125.69, 124.92 (d, *J* = 12.0 Hz), 123.43 (d, *J* = 7.4 Hz), 121.74 (d, *J* = 3.5 Hz), 115.17 (d, *J* = 18.2 Hz), 83.50 (d, *J* = 2.7 Hz), 68.89, 46.92, 42.44, 32.33, 26.80. ^{19}F NMR (471 MHz, $CDCl_3$), δ [ppm] = -135.39.

7-fluoro-2,3,4,4a-tetrahydro-4,10b-methanopyrano[3,2-c]quinolin-5(6H)-one (8, crossed product)

The crude product was purified by flash chromatography (2:1 ethyl acetate:cyclohexane) to give the product as a white solid (9 mg, 18 %). HR-MS (ESI) *m/z*: found $[M+H]^+$ 234.1145; calc.: 233.0852. ^{1}H NMR (400 MHz, $CDCl_3$, 298K), δ [ppm] = 7.71 (s, 1H, *NH*), 7.20 – 7.15 (m, 1H, *Ph*), 7.04 – 6.96 (m, 2H, *Ph*), 4.40 (dt, *J* = 11.6, 7.8 Hz, 1H, *CH*), 4.26 (td, *J* = 7.7, 4.0 Hz, 1H, *CH*), 3.15 – 3.08 (m, 1H, *CH*), 2.90 (d, *J* = 8.1 Hz, 1H, *CH*), 2.55 (dd, *J* = 11.1, 5.7 Hz, 1H, *CH*), 2.42 – 2.35 (m, 1H, *CH*), 2.31 – 2.24 (m, 1H, *CH*), 2.20 (dd, *J* = 11.1, 7.9 Hz, 1H, *CH*). ^{13}C NMR (126 MHz, $CDCl_3$, 298K) δ 171.11, 149.40 (d, *J* = 243.0 Hz), 127.16, 123.80 (d, *J* = 12.3 Hz), 123.52 (d, *J* = 7.3 Hz), 119.51 (d, *J* = 3.2 Hz), 115.00 (d, *J* = 18.2 Hz), 80.12, 60.99, 51.21, 36.97, 36.58, 31.54. ^{19}F NMR (376 MHz, $CDCl_3$, 298K) δ -135.40.

8-methoxy-3,3a,4,4a-tetrahydro-2H-furo[2',3':2,3]cyclobuta[1,2-c]quinolin-5(6H)-one (9a, straight product)

The crude product was purified by flash chromatography (100% ethyl acetate) to give the product as a white solid (42 mg, 84 %). HR-MS (ESI) *m/z*: found $[M+H]^+$ 246.1176; calc.: 245.1052. ^{1}H NMR (500 MHz, $CDCl_3$, 298K), δ [ppm] = 8.76 (s, 1H, *NH*), 7.18 (d, *J* = 8.6 Hz, 1H, *Ph*), 6.59 (dd, *J* = 8.6, 2.4 Hz, 1H, *Ph*), 6.31 (d, *J* = 2.6 Hz, 1H, *Ph*), 4.41 (t, *J* = 8.6 Hz, 1H, *CH*), 4.22 – 4.15 (m, 1H, *CH*), 3.78 (s, 3H, CH_3), 3.33 (dd, *J* = 12.2, 7.8 Hz, 1H, *CH*), 2.93 – 2.84 (m, 1H, *CH*), 2.41 – 2.34 (m, 1H, *CH*), 2.18 – 2.10 (m, 2H, CH_2), 1.82 (dd, *J* = 12.9, 5.7 Hz, 1H, *CH*). ^{13}C NMR (126 MHz, $CDCl_3$, 298K), δ [ppm] = 172.53, 160.82, 137.61, 128.05, 116.14, 110.04, 101.36, 83.57, 68.63, 55.84, 46.83, 42.73, 32.71, 26.89.

Preparation of chiral standards

The enantiomers of **1a** were separated by preparative chiral UPLC by Reach Separations (Nottingham) to afford (-)-**1a** (98.2% e.e.) and (+)-**1a** (99.6% e.e.) as white solids. The absolute stereochemistry was determined by measuring the optical rotation (-)-**1a** (−72.0°) and (+)-**1a** (+72.0°) at 0.017 g mL^{-1} in methanol (MeOH) at 26 °C.

Reductive dehalogenation of 7a to 1a

Product **7a** (5 mg, 0.017 mmol, 1 eq., 84% e.e.) obtained from the preparative-scale biotransformation of **7** with CEnT1.3 S271V A272G Y121A, paraformaldehyde (0.5 mg, 0.017 mmol, 1 eq.), Cs_2CO_3 (7.8 mg, 0.024 mmol, 1.4 eq.), $PdCl_2[P(o\text{-}Tol)_3]_2$ (0.67 mg, 5 mol%) was dissolved in 0.5 mL of dry dimethylformamide and stirred at 80 °C for 18 h under nitrogen.[4] The reaction was cooled to room temperature, poured into water (10 mL) then extracted with ethyl acetate (3 × 10 mL). The combined organic layers were washed with water (3 × 20 mL), dried over $MgSO_4$, filtered and the solvent removed *in vacuo* to give the crude product. The

crude product was purified by flash chromatography (1:2 ethyl acetate:hexane) to give the product as a white solid (1 mg, 27.4% isolated yield). ^{1}H NMR (400 MHz, $CDCl_3$, 298K) δ 7.61 (s, 1H), 7.29 (d, J = 8.6 Hz, 1H), 7.22 (t, J = 7.8 Hz, 1H), 7.05 (t, J = 7.6 Hz, 1H), 6.69 (d, J = 7.9 Hz, 1H), 4.45 (t, J = 8.7 Hz, 1H), 4.23 (t, J = 13.0 Hz, 1H), 3.39 – 3.32 (m, 1H), 2.96 (d, J = 3.5 Hz, 1H), 2.44 – 2.37 (m, 1H), 2.21 – 2.10 (m, 2H), 1.84 (dd, J = 12.9, 5.7 Hz, 1H).

Crystallisation, refinement and model building

Crystallisation conditions were identified using the JCSG Plus screen (Molecular Dimensions). All trials were conducted by sitting-drop vapour diffusion and incubated at 25 °C. CEnT1.4 was crystallised by mixing 200 nL of 15 mg mL^{-1} protein in 25 mM HEPES pH 7.0 with an equal volume of mother liquor (2.0 M ammonium sulphate, 0.2 M sodium chloride, 0.1 M sodium cacodylate, pH 6.5).

Prior to data collection, crystals were cryo-protected by the addition of 20% PEG 200 to the mother liquor and plunge cooled in liquid nitrogen. Diffraction data was collected at Diamond Light Source (Harwell, UK) using beamline i03. Data reduction was performed with Dials and the structure solved by molecular replacement using a search model derived from PDB: 7ZP5. Iterative rounds of model building and refinement were performed in COOT and Phenix.refine, respectively.[5] Validation with MOLPROBITY and PDBREDO[6] were incorporated into the iterative rebuild and refinement process. Data collection and refinement statistics are shown in Table S3. The coordinates and structure factors have been deposited in the Protein Data Bank under accession number 9ENO.

Molecular Docking

The product (+)-**1a** and substrate **1** models were built using Chemdraw 3D, and were subsequently geometry optimized using density functional theory (DFT) at the M062x/6-31+g(d,p) level of theory using Gaussain09[7] The optimized structures were docked into the CEnT1.4 X-ray crystal structure using Autodock Vina.[8] Poses similar to those observed in the X-ray crystal structure of the EnT1.3–product (-)-**1a** complex (PDB: 7ZP7). were chosen for subsequent molecular dynamics (MD) simulations.

MD simulations

MD simulations of CEnT1.4 with non-canonical amino acid residue BpA were performed using Gromacs2021.[9] The non-canonical amino acid BpA was parameterized using Amber atom types. The coordinates of BpA were obtained from the crystal structure of EnT1.3 (PDB: 7ZP7). For parametrization, the C- and N-termini of this residue were capped with methyl groups. The capped BpA underwent geometry optimization using the HF/6-31G* level of theory using Gaussian09.[7] Atomic partial charges were then calculated by RESP fitting using the Antechamber package in Ambertools21.[10] Subsequently, the caps at the C- and N-termini were removed, and their charges were redistributed to the C-termini and N-termini. Substrate **1** and product (+)-**1a** were parameterized using Ambertools21.[10] The atom charges for BpA, the substrate **1**, and product (+)-**1a** are given in Tables S7-9.

The AMBER FF19SB force field[11] was used for the protein and the complexes were solvated with 10604 TIP3P water molecules in a cubic box at least 10 Å larger than the protein complex on each axis and the system was neutralized with the addition of 7 Na+ ions. Each system was first energy minimized or 5000 steps and heated to 298 K over a period of 100 ps, followed by 100ps of constant pressure (NPT, 1 bar) and then100ps of constant volume (NVT) MD simulations. Production MD simulations were then run in triplicate for 200ns. All simulations were run at constant temperature (298 K) and pressure (1 bar) with a 10 Å cut-off for short-range electrostatic and van der Waals interactions, and LINCS constraints were applied to all bonds involving hydrogen.

Supporting Figures

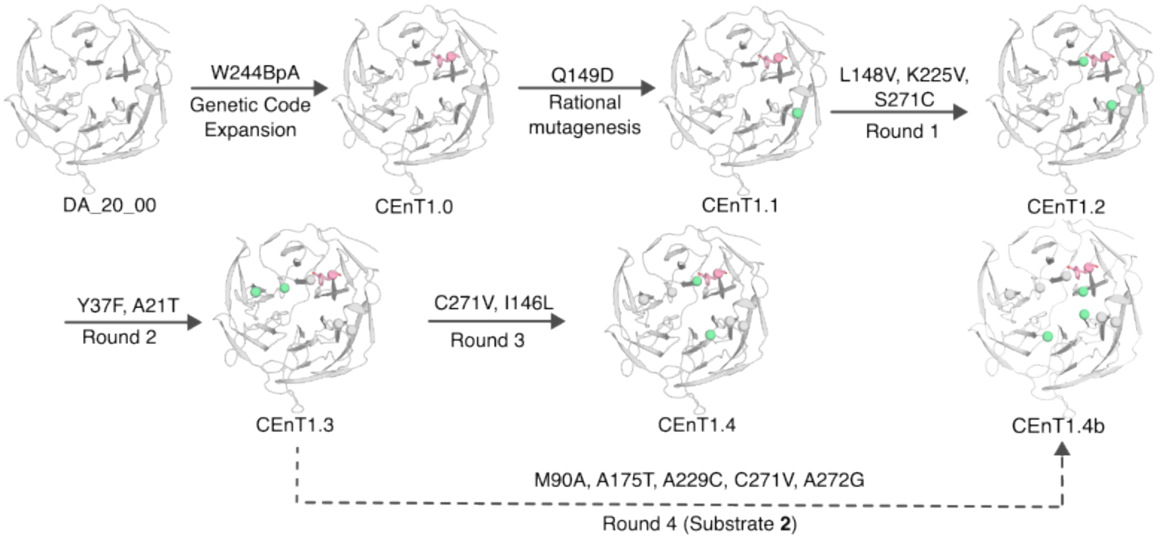

Round	Description	Clones Screened	Substrate	Beneficial Mutations	Best Variant
Rational Mutagenesis	**Rational mutagenesis** of active site positions: Q149D, Y37F, H279A, P196R, I146A, L148W	6	**1**	Q149D	CEnT1.1= CEnT1.0 + Q149D
1	**Saturation mutagenesis** of active site positions: A21, Y37, A74, M90, A120, Y121, P135, I146, L148, Q149, A173, A175, P196, K225, G227, A229, A242, P254, K269, S271, H287, N290,	1,936	**1**	L148V K225V P196A S271T S271V S271C	CEnT1.2= CEnT.1 + L148V, K225V, S271C
2	**Saturation mutagenesis** of active site positions: A21, Y37, I72, A74, M90, A120, Y121,P135, F144, I146, L148, Q172, A173, Q195, P196, A229, A242, K269, A272, H287,	1,760	**1**	A21T Y37F A272G L146V	CEnT1.3= CEnT1.2 + Y37F, A21T
3	**Saturation mutagenesis** of active site positions: V34, P36, I72, A74, M90, A136, F144, T145, I146, S147, L148, D149, Q172, A173, V175, Q195, P196, G227, A229, C271, A272, T285, H287	2,024	**1**	C271V A272G I146L L148V	CEnT1.4= CEnT1.3 + C271V, I146L
4	**Saturation mutagenesis** of active site positions: A20, G22, P36, V38, I72, P73, A74, M90, A120, Y121, P135, L146, S147, P174, A175, A228, A229, P270, E286, E288	1,760	**2**	M90A A120C A175T A229C	CEnT1.4b= CEnT1.3 + C271V +A272G + M90A A175T A229C

Figure S1 | Directed evolution of an efficient and selective enantiocomplementary photoenzyme. Schematic showing the trajectory from CEnT1.0 to CEnT1.4 and CEnT4b. Mutations introduced are represented as CPK spheres at the C-alpha. The original DA_20_00 scaffold has a β-propeller fold with a central cavity (PDB 1E1A; a diisopropylfluorophosphatase from *Loligo vulgaris*). Incorporation of the photosensitizer BpA (pink atom-coloured sticks and semitransparent CPK spheres) at position W244 provided CEnT1.0. Rational mutagenesis of CEnT1.0 highlighted one variant with increased activity, providing CEnT1.1. Three rounds of evolution afforded CEnT1.4, which contains nine

mutations when compared with the original design DA_20_00. Rational mutagenesis and libraries generated for rounds 1–3 were screened for activity towards substrate **1**. One further round of evolution was performed on CEnT1.3_C271C_A272G for activity towards substrate **2** to deliver CEnT1.4b. Library generation method, positions targeted, the number of clones evaluated, beneficial mutations and the most improved variant for each round are given in the associated table.

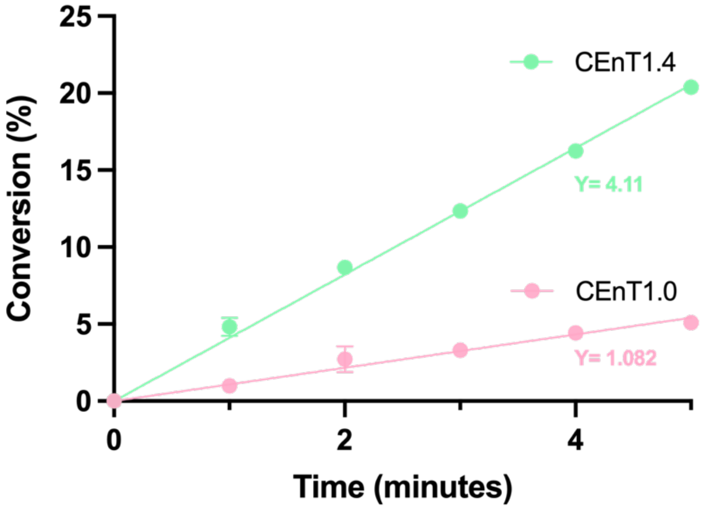

Figure S2 | Reaction time courses of CEnT1.0 and CEnT1.4. Reaction conditions: 15 µM enzyme, 400 µM 1, 10 s on/off pulse at 365 nm, 4 °C, 1 mL PBS (pH 7.4) with 5% DMSO as a cosolvent in 2 mL glass vials. At each time point, 100 µl of reaction mix was quenched with one volume of MeCN and analysed by UPLC. Reaction conversion of **1** to **1a** and **1b** is given over 5 min. The gradients of a linear fit to the data are given for each variant. Error bars represent the standard deviation of measurements made in triplicate (error bars are not shown when they are smaller than the data-point marker).

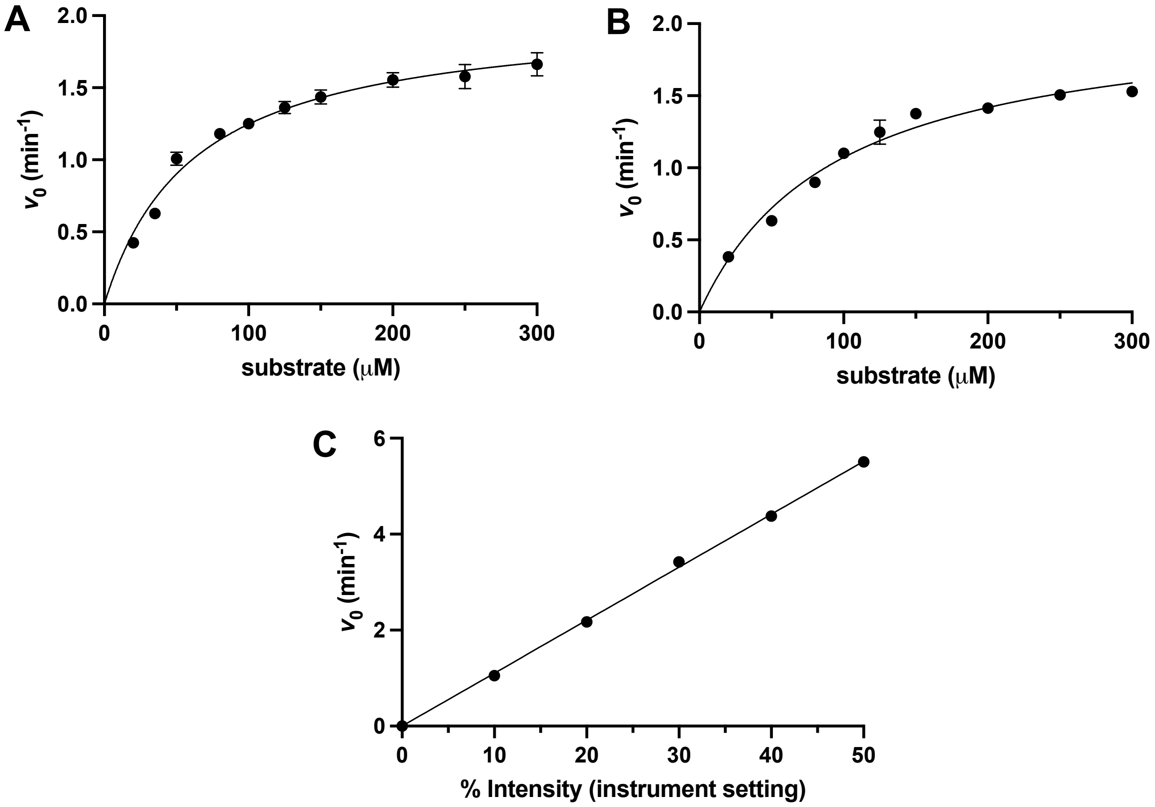

Figure S3 | Kinetic characterisation of CEnT1.4. A) Michaelis-Menten plot for the intramolecular [2+2]-cycloaddition of substrate **1** catalysed by CEnT1.4. Kinetic assays were performed at various concentrations of **1**, and 1 μM of CEnT1.4 in 250 μL PBS (pH 7.4) with 5% DMSO as a co-solvent in 96-well plate. The plots show the averaged initial rates which were fitted to the Michaelis-Menten equation using Origin software. Data are a mean ± S.D. of measurements made in triplicate. **B)** Michaelis-Menten plot for the intramolecular [2+2]-cycloaddition of substrate **1** catalysed by EnT1.3. Kinetic assays were performed at various concentrations of **1**, and 1 μM of EnT1.3 in 250 μL PBS (pH 7.4) with 5% DMSO as a co-solvent in 96-well plate. The plots show the averaged initial rates which were fitted to the Michaelis-Menten equation using Origin software. Data are a mean ± S.D. of measurements made in triplicate. **C)** The dependence of CEnT1.4 v_0 on light intensity. Assays were performed with 400 μM of substrate **1** and 1 μM of CEnT1.4 in 500 μL PBS (pH 7.4) with 5% DMSO as a co-solvent in 2 mL glass vials. Intensity was varied from 0%-50% (based on instrument settings) with the reactions irradiated 3 cm directly below the LED array.

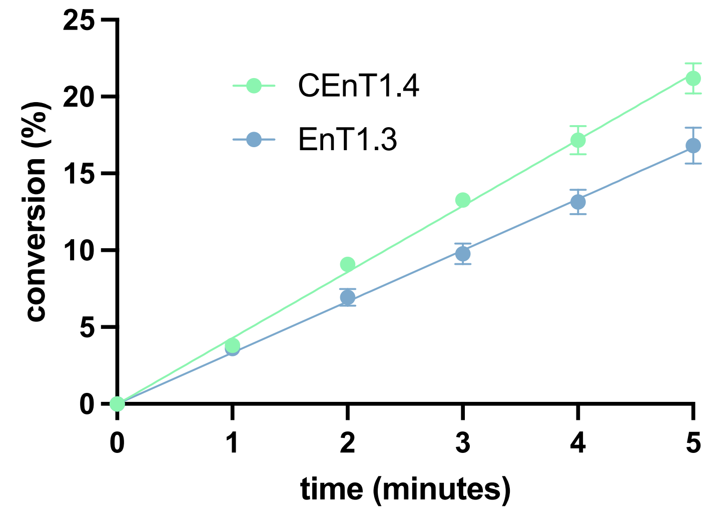

Figure S4 | Reaction time courses of CEnT1.4 and EnT1.3. Reaction conditions: 15 µM enzyme, 400 µM 1, 10 s on/off pulse at 365 nm, 4 °C, 1 mL PBS (pH 7.4) with 5% DMSO as cosolvent in 2 mL glass vials. At each time point, 100 µL of reaction mix was quenched with one volume of MeCN and analysed by UPLC. Reaction conversion of **1** to **1a** is given over 5 min. The gradients of a linear fit to the data are given for each variant. Error bars represent the standard deviation of measurements made in triplicate (error bars are not shown when they are smaller than the data-point marker).

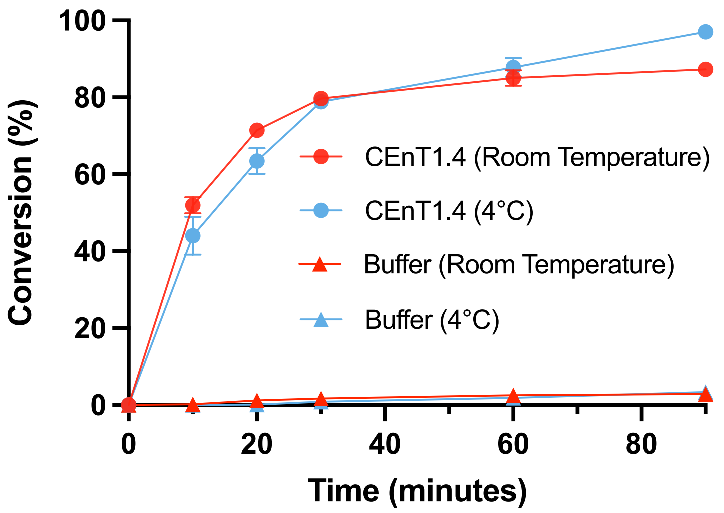

Figure S5 | Temperature dependence of CEnT1.4. Reaction conditions: 20 µM CEnT1.4 (5 mol%), 400 µM **1**, 10 s on/off pulse at 365 nm, in PBS (pH 7.4) with 5% DMSO as a cosolvent at a specified temperature. A time course providing the conversion of **1** to **1a** and **1b** at 4 °C (blue circles) and room temperature (red circles). A negative control containing no catalyst is shown at 4 °C and room temperature (blue triangles and red triangles, respectively). Error bars represent the standard deviation of measurements made in triplicate.

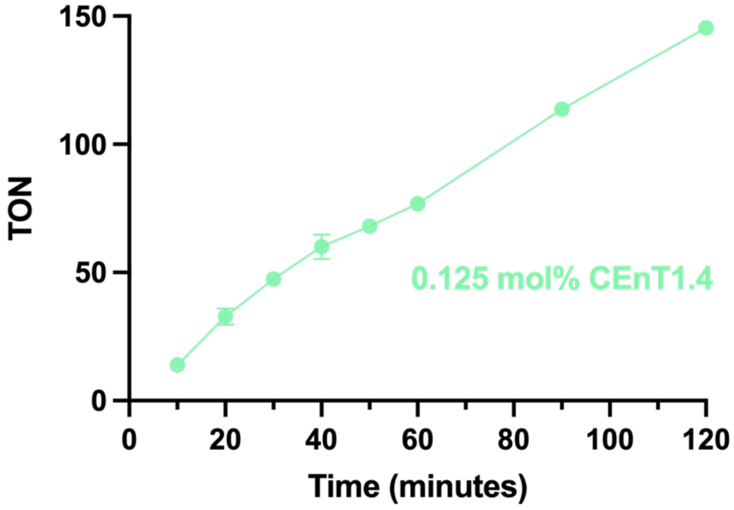

Figure S6 | Total turnover numbers achieved by CEnT1.4. Reaction conditions: 800 µM **1**, 10 s on/off pulse at 365 nm, at 4 °C, in 1 mL PBS (pH 7.4) with 10% DMSO as a cosolvent, at a 0.125 mol% catalyst loading. A time course providing the reaction conversion of **1** to **1a** at 0.125 mol% catalyst loading (1 µM (green)). A background correction has been applied to both curves, corresponding to less than 10% of turnovers. Error bars represent the standard deviation of measurements made in triplicate (error bars are not visible when smaller than the data-point marker).

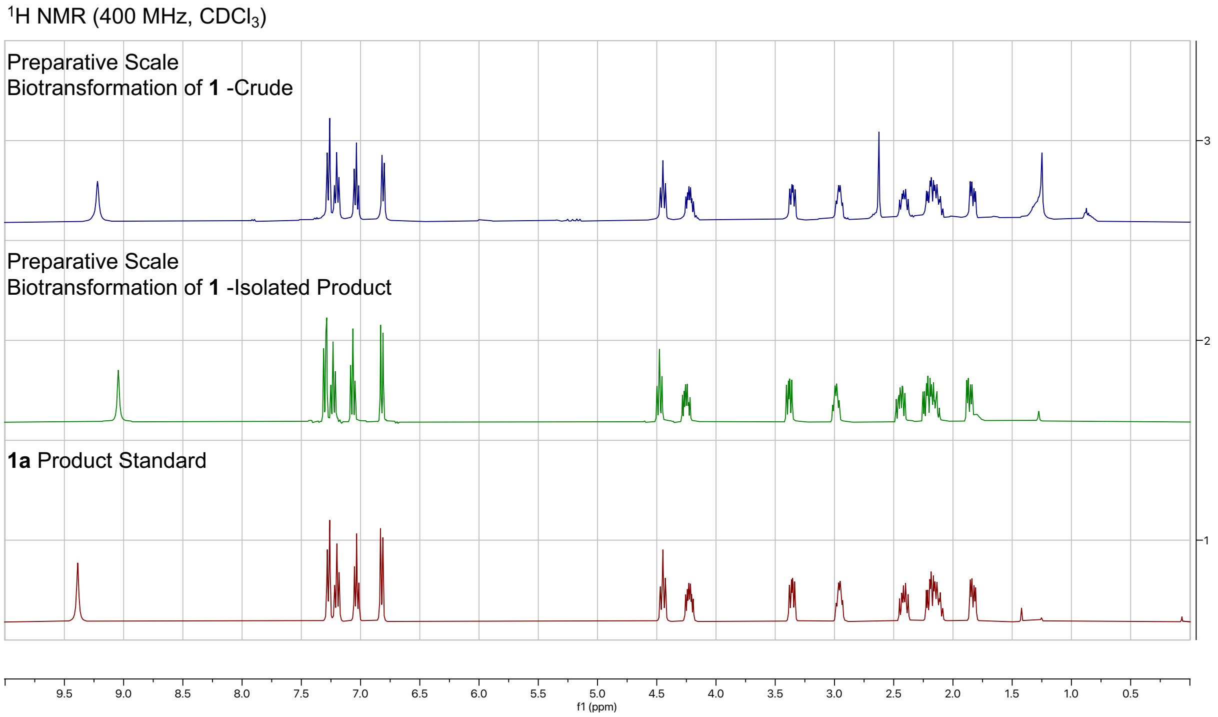

Figure S7 | Preparative 11.4 mg Scale Biotransformation of Substrate 1. ^{1}H NMR spectra (400 MHz, $CDCl_3$) of **1a** chemically synthesised product standard, and crude product extracted from the CEnT1.4 (20 µM) catalysed intramolecular [2+2]- cycloaddition of substrate **1** (400 µM) in PBS (pH 7.4) with 5% DMSO as a co-solvent (133 mL reaction volume).

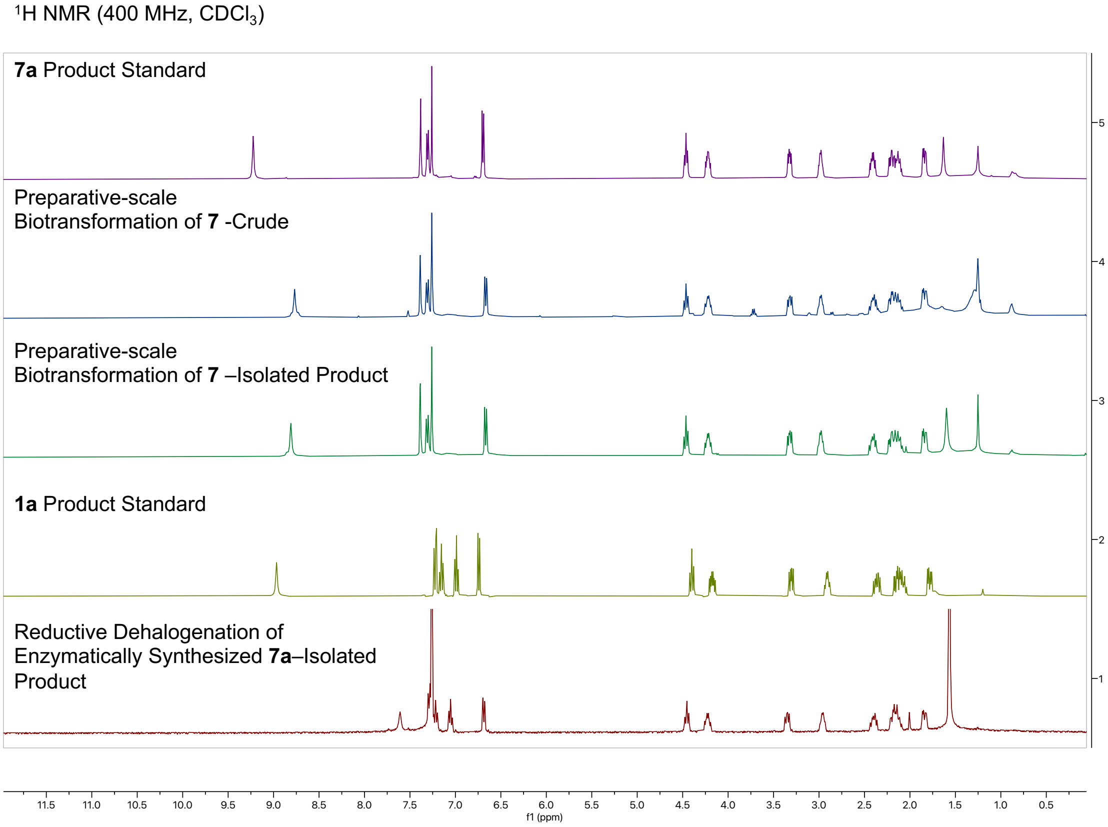

Figure S8 | ^{1}H NMR spectra (400 MHz, $CDCl_3$) for the Preparative 10 mg Scale Biotransformation of substrate 7 and subsequent reductive dehalogenation to 1a. A) A chemically synthesised product standard of **7a**. B) crude product C) and isolated product extracted from the preparative-scale biotransformation of substrate **7** (400 µM) with CEnT1.3_C271V_A272G_Y121A (50 µM) in PBS (pH 7.4) with 10% DMSO as a co-solvent (85 mL reaction volume). D) A chemically synthesised product standard of **1a**. E) isolated product from the reductive dehalogenation of product **7a** obtained from the preparative-scale biotransformation of substrate **7** to ultimately yield **1a**.

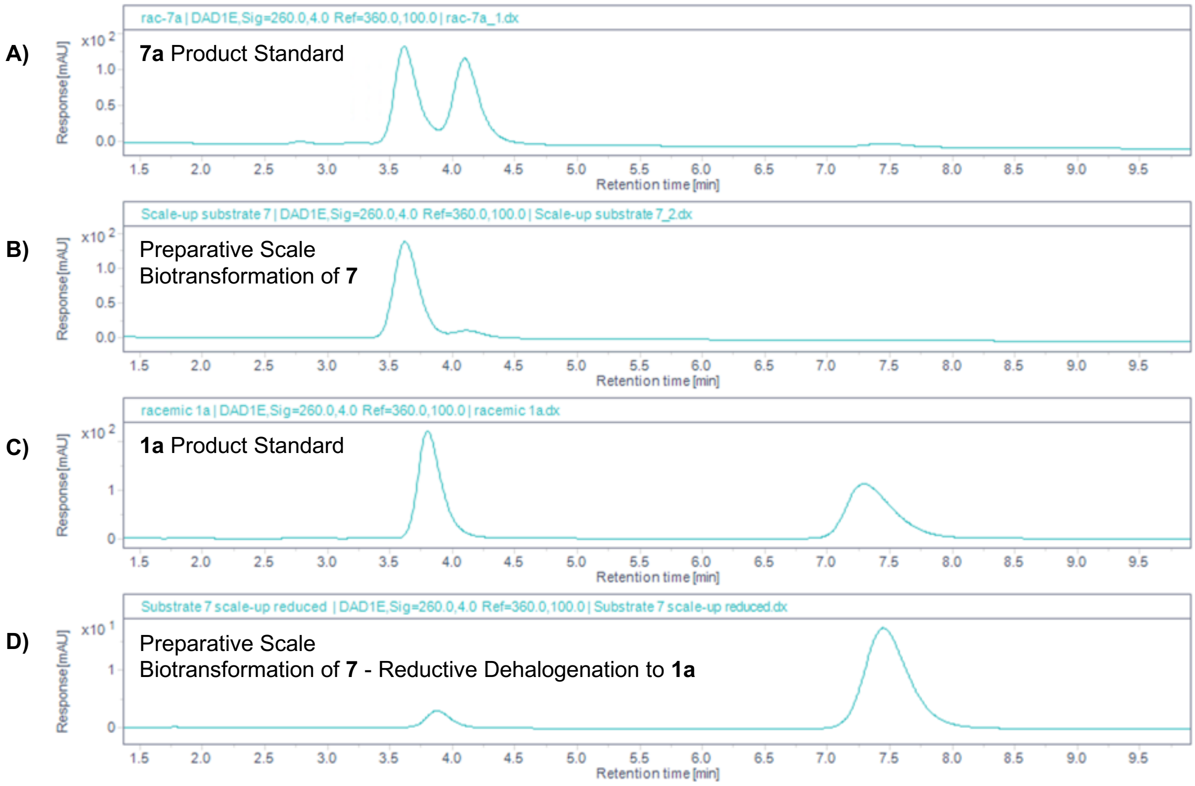

Figure S9 | UPLC chromatograms for the Preparative 10 mg Scale Biotransformation of substrate 7 and subsequent reductive dehalogenation to 1a. A) A chemically synthesised racemic product standard of **7a**. B) Isolated product extracted from the preparative-scale biotransformation of substrate **7** (400 µM) with CEnT1.3_C271V_A272G_Y121A (50 µM) in PBS (pH 7.4) with 20% DMSO as a co-solvent (>99% conversion, 84% e.e.). C) A chemically synthesised racemic product standard of **1a**. D) Isolated product from the reductive dehalogenation of product **7a** obtained from the preparative-scale biotransformation of substrate **7** to ultimately yield **1a** (83% e.e.). For SFC analysis, reactions were extracted with 3 volumes of ethyl acetate. The conditions for the reductive dehalogenation can be found in the 'Chemical Procedures' of the Supplementary Information.

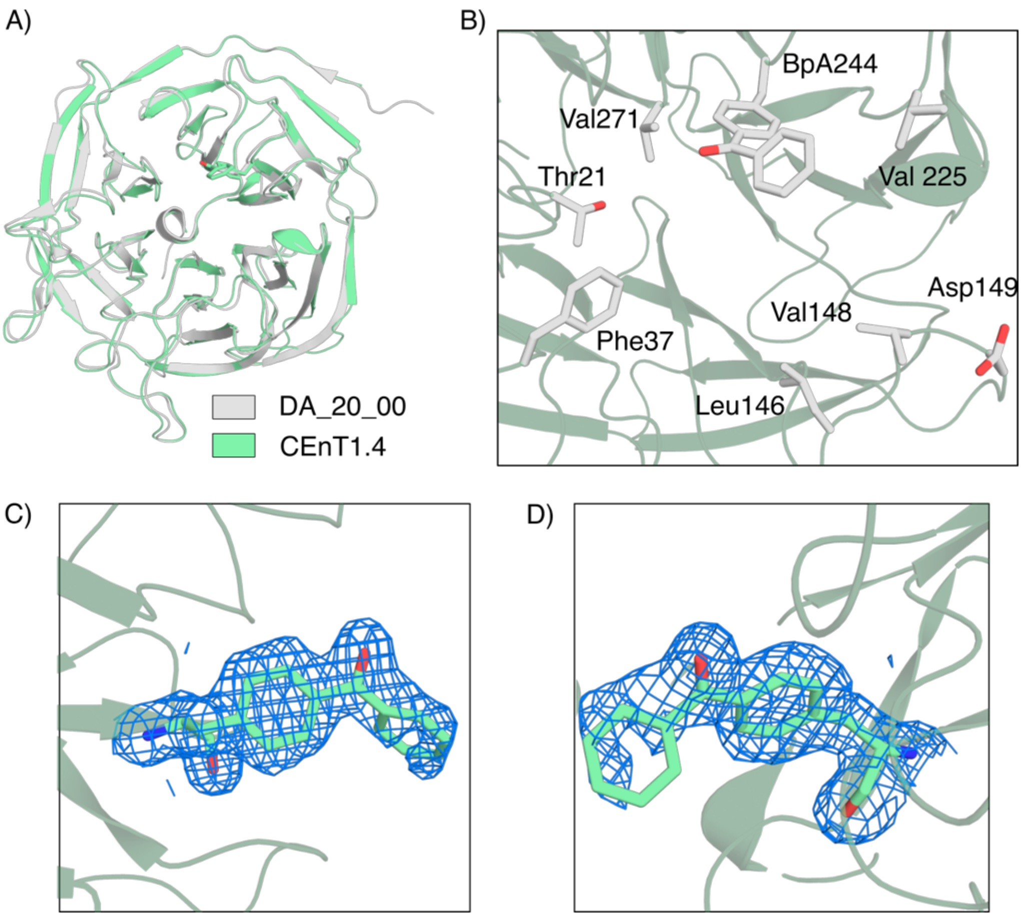

Figure S10 | Crystal structure of CEnT1.4 A) An overlay of DA_20_00 (PDB: 3I1C) and CEnT1.4 (PDB: 9ENO), shown as grey and green ribbons, respectively. The BpA at position 244 of CEnT1.4 is shown in atom-coloured sticks with green carbon atoms. **B)** Mutations introduced during directed evolution of CEnT1.4, and the photosensitiser are shown as atom-coloured residue sidechains. **C)** and **D)** BpA244 is shown in the crystal structure of CEnT1.4, with the electron density shown as a blue mesh (2Fo-Fc map contoured at 0.6 σ).

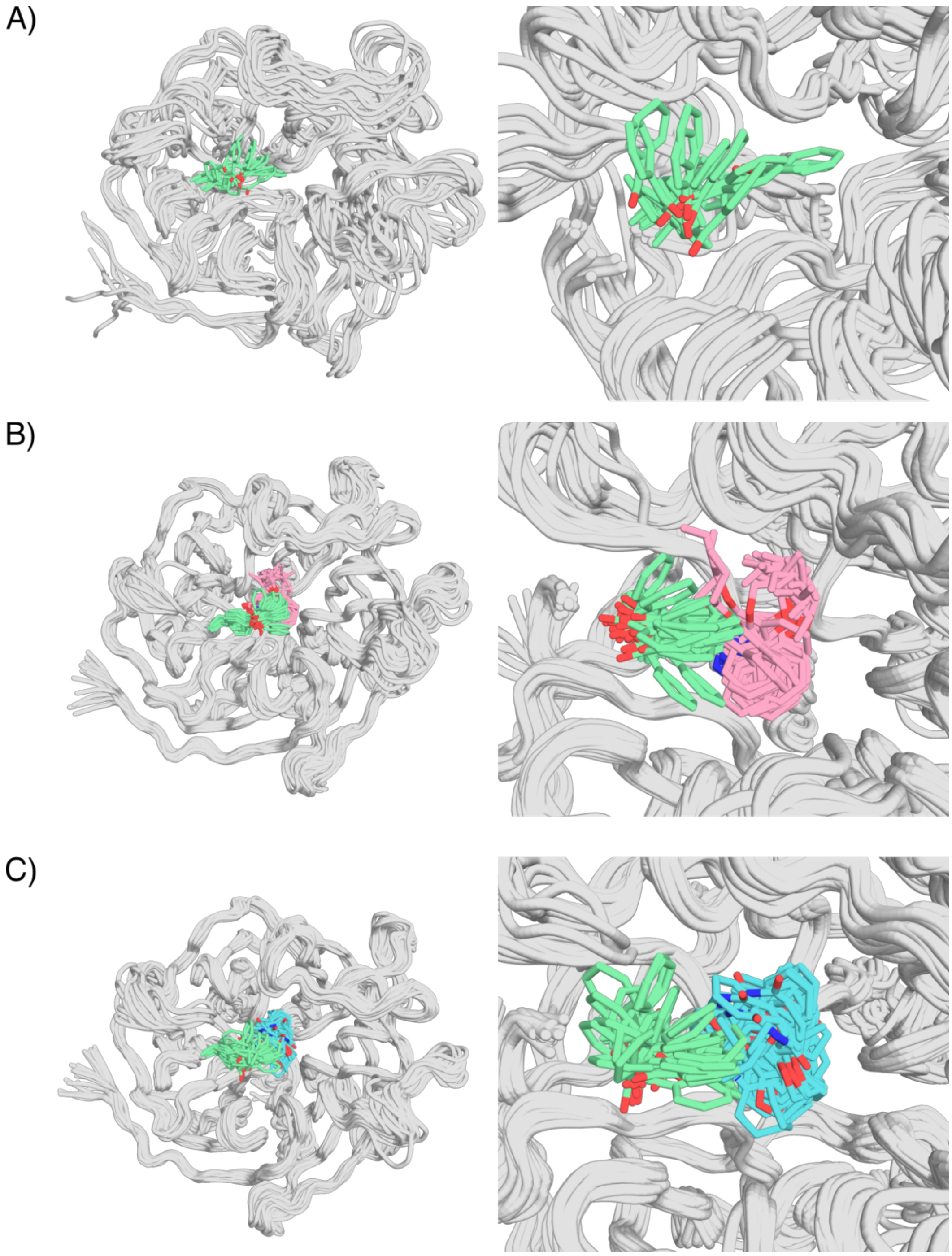

Figure S11 | Overlaid poses from MD trajectories. A) CEnT1.4, **B)** substrate-bound CEnT1.4 and **C)** product-bound CEnT1.4 complexes. Frames were selected by sampling every 20 ns, to illustrate the protein and ligand motion. The protein backbone is shown as a grey ribbon. The non-canonical residue BpA244, substrate **1**, and product (+)-**1a** are shows as atom-coloured sticks with green, pink, and blue carbons, respectively.

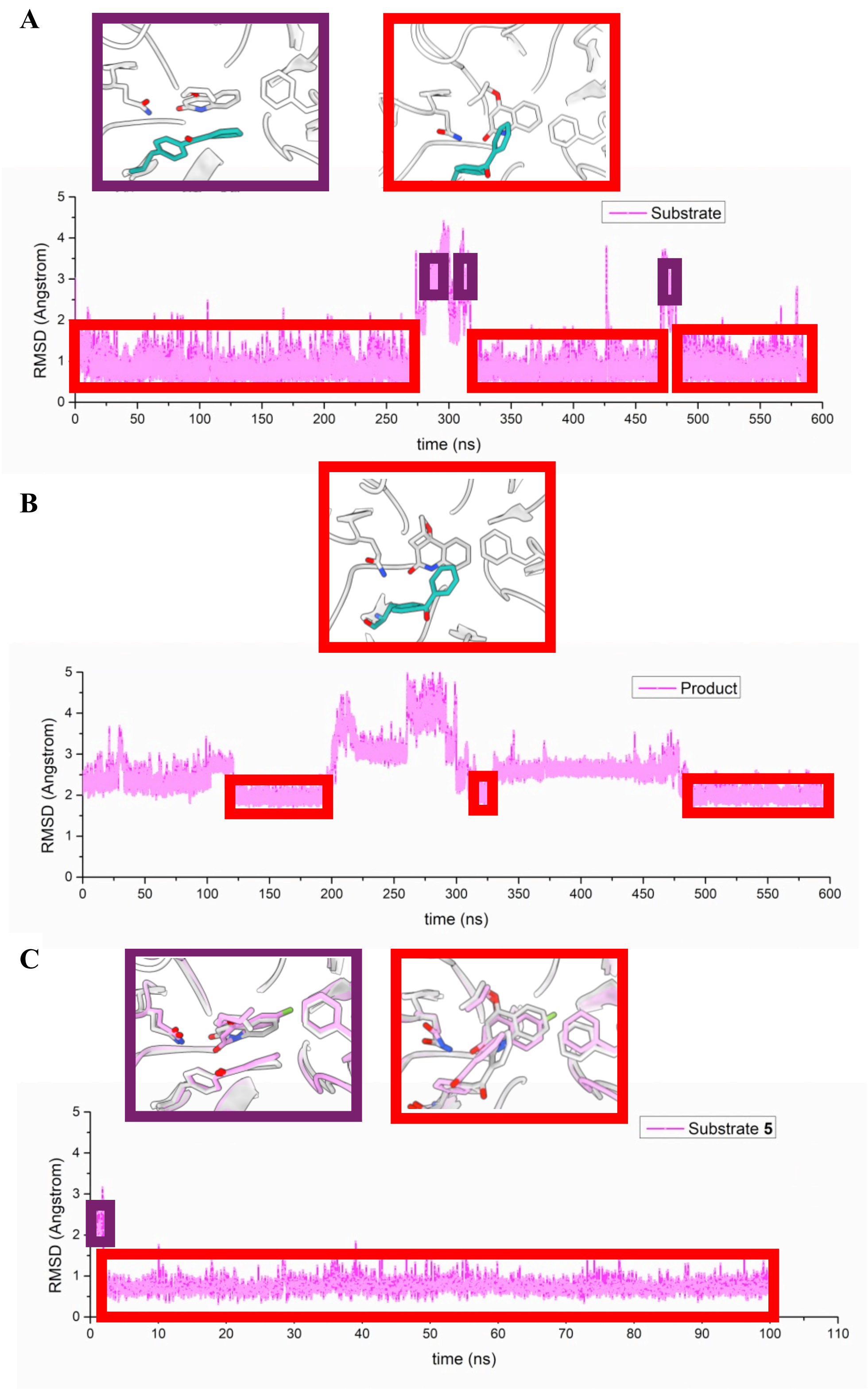

Figure S12 | MD trajectories of substrate and product simulations. A) CEnT1.4-substrate **1** complex. **B)** CEnT1.4-product (+)-**1a** complex. **C)** CEnT1.4-substrate **5** complex. For **A)** and **B)**, 200ns MD simulations were run in triplicate and then combined to create these trajectories, which plot the substrate **1** and product **(+)-1a** RMSD, respectively. A single 100 ns simulation was run with substrate **5** in **C)** The major populations are outlined in a red box, while the minor populations are outlined in the purple box. Substrate **5** has two conformations (shown in white) which are identical to the two confirmations observed with substrate **1** (shown in pink).

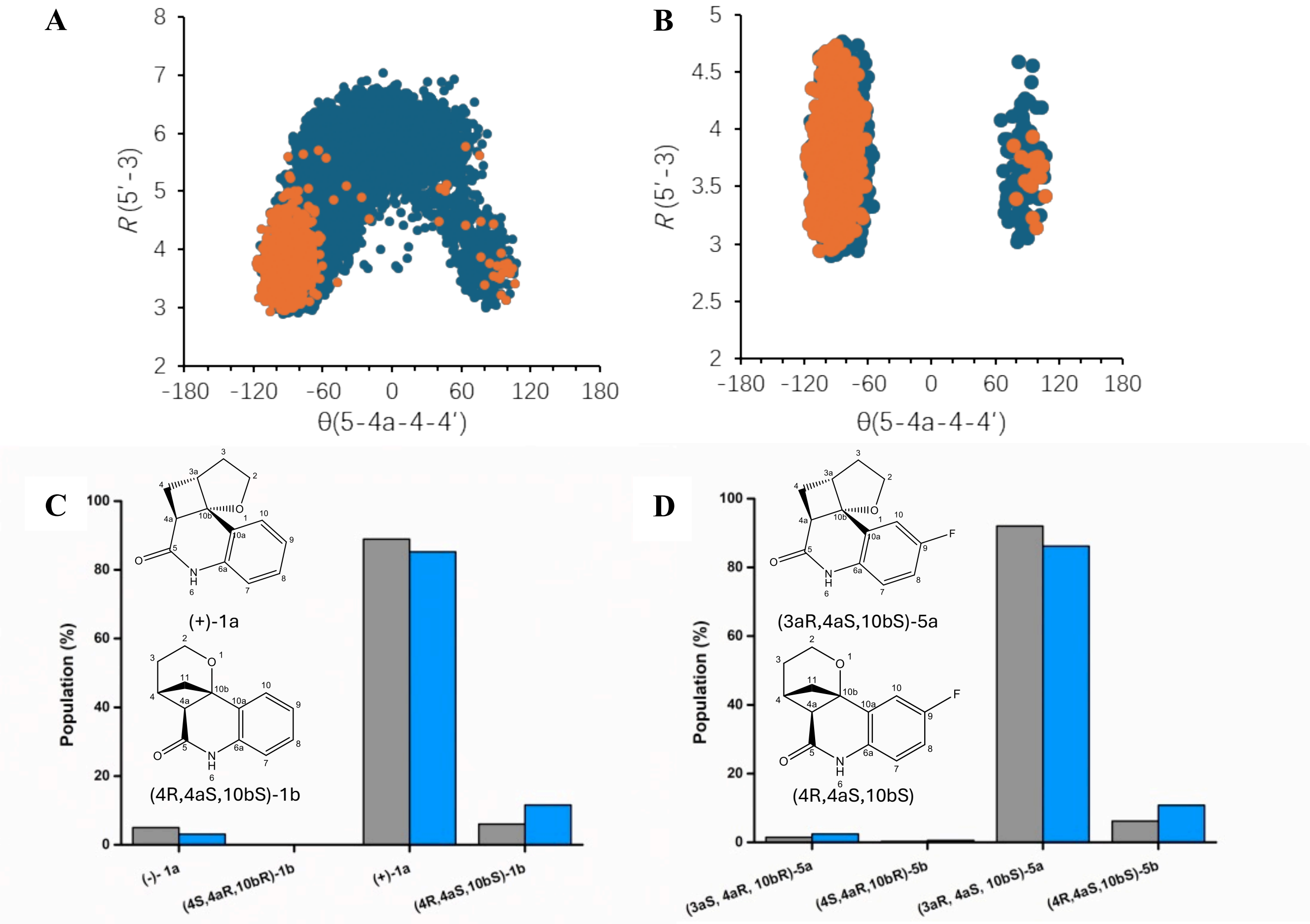

Figure S13 | Additional population analysis of MD simulations for CEnT1.4–substrate complexes. Populations *R*(4′ - 4) < *R*(4′ - 3) (blue) and *R*(4′ - 4) > *R*(4′ - 3) (orange) are mapped onto the *R*(5′ - 3) distances and plotted *vs.* θ(5 – 4a – 4 - 4′). **A)** shows all data (as shown in Figure 3C) and **B)** shows data points only where *R*(4′ - 4) < 3.5 Å for the CEnT1.4-substrate **1** complex. **C)** A bar chart displaying the populations represented throughout the MD trajectory for the CEnT1.4-substrate **1** complex. Conformers were assigned based on dihedral and distance features that could give rise to formation of product (-)-**1a,** (+)-**1a** or the corresponding cross products, (4*S*, 4a*R*, 10b*R*)-**1b** and (4*R*, 4a*S*, 10b*S*)-**1b**. **D)** A bar chart describing the populations represented throughout the MD trajectory for CEnT1.4-substrate **5** complex. The conformers from the MD simulation have the same regio- and enantio- preferences described with the simulation of CEnT1.4-substrate **1** complex. Grey and blue bars represent all the data and the selected data where *R*(4′ - 4) < 3.5 Å, respectively. These data are also given in Table S4.

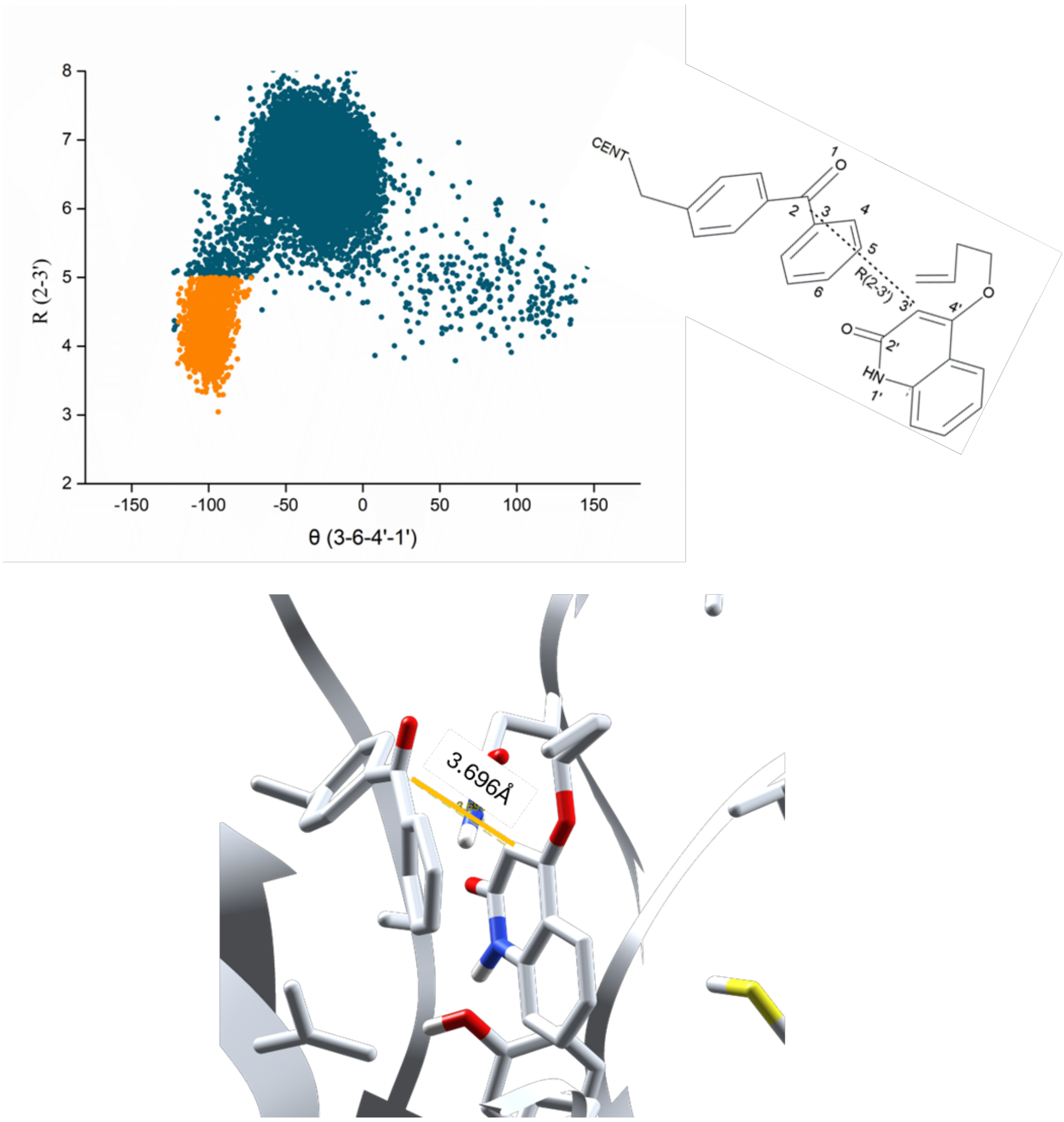

Figure S14 | Additional analysis of BpA-substrate π stacking in MD simulations of the CEnT1.4–substate complex. We expect that efficient energy transfer will occur when BpA and substrate have π-π orbital overlap (*i.e. via* π stacking).[10] (top) We defined a π-stacked configuration as one where -120º < θ(3-6-4'-1')< -60 º and *R*(2-3') < 5 Å (scheme top right). The scatter plot maps the population (blue) onto the *R* (2-3') distance and θ(3-6-4'-1') dihedral. Those data with a π-stacked configuration based on the dihedral and distance criteria is shown in orange. (bottom) An example snapshot from the MD trajectory, which shows a π-stacked configuration with θ(3-6-4'-1') = -92º and *R*(2-3') = 3.70 Å.

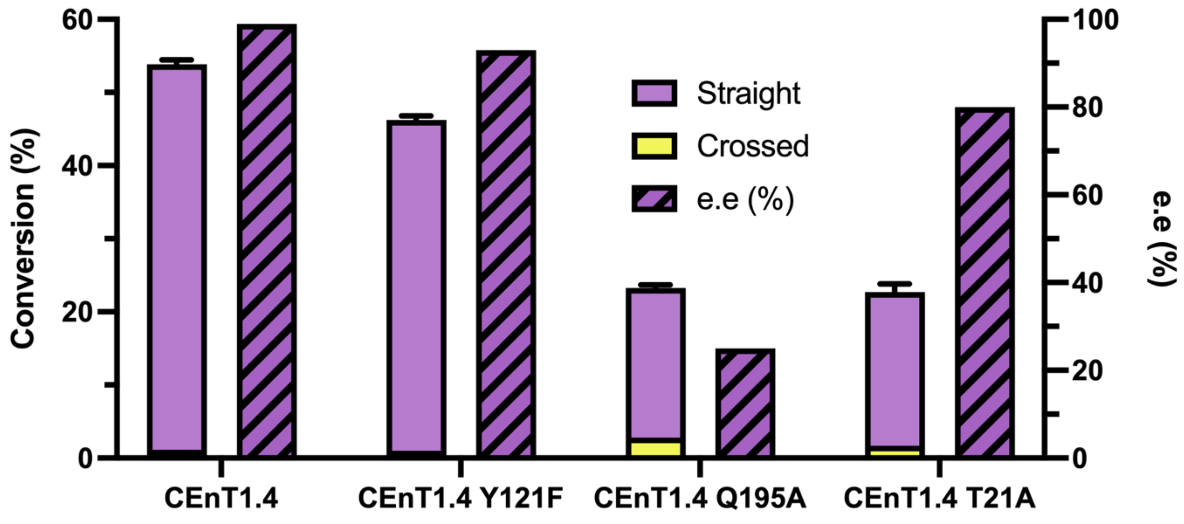

Figure S15 | Knockout variants of CEnT1.4. Reaction conditions: 10 µM catalyst, 400 µM **1**, 30 min irradiation (10 s on/off pulse) at 365 nm, 4 °C, 100 µL reaction volume in PBS (pH 7.4) with 5% DMSO as a cosolvent in 1mL glass vials. Bar chart showing conversions and *e.e.* for the major product, (+)-**1a**, achieved by CEnT1.4 variants. Error bars represent the standard deviation of measurements made in triplicate.

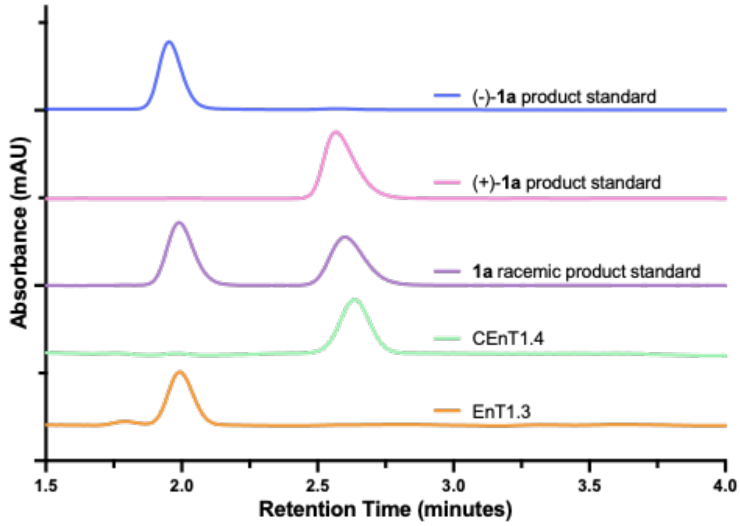

Figure S16 | SFC chromatograms of racemic and optically pure product standards of 1a, and the product of EnT1.3-catalysed and CEnT1.4 reactions using substrate 1. Reaction conditions: 20 µM EnT1.3 or CEnT1.4, 400 µM **1**, 30 minutes irradiation (10 seconds on/off pulse) at 365 nm, at 4 °C, in 100 µL PBS (pH = 7.4) with 5% DMSO as a cosolvent. For SFC analysis, reactions were extracted with 3 volumes of ethyl acetate.

Supporting Tables

Table S1: Summary of conversion and selectivity values of CEnT1.0. Reaction conditions: 15 µM catalyst (BP or protein variant), 400 µM **1**, 30 min total irradiation (10 seconds on/off pulse) at 365 nm, at 4 °C, PBS (pH 7.4) with 5% DMSO as a cosolvent. Standard deviations are given for measurements in triplicate.

	Average Conversion Straight (%) 1a	S.D.	Average Conversion Crossed (%) 1b	S.D.	Straight e.e. (%) 1a	S.D.
**Buffer**	0.8	0.3	0.5	0.01	0.00	-
**BP**	2.3	0.4	2.1	0.4	0.00	-
**DA_20_00 30BpA**	1.1	0.1	0.7	0.1	0.00	-
**EnT1.0**	6.3	1.0	3.4	0.6	-44.8	4.1
**DA_20_00 244BpA 173Trp**	3.8	0.5	1.6	0.2	57.6	3.0
**CEnT1.0 (with light)**	13.7	0.2	3.0	0.3	26.2	2.7
**CEnT1.0 (no light)**	-	-	-	-	-	-

Table S2: Summary of conversion and selectivity values of CEnT1.0 variants along the evolution trajectory. Reaction conditions: 10 µM catalyst (BP or protein variant), 400 µM **1**, 60 min total irradiation (10 seconds on/off pulse) at 365 nm, at 4 °C, PBS (pH 7.4) with 5% DMSO as a cosolvent. Standard deviations are given for measurements in triplicate.

	Average Conversion Straight (%)	S.D.	Average Conversion Crossed (%)	S.D.	Straight e.e. (%)	S.D.
**BP**	3.8	0.7	1.1	0.9	-	-
**CEnT1.0**	13.6	0.7	3.0	0.2	26.2	3.9
**CEnT1.1**	16.1	0.2	3.8	0.02	33.8	2.8
**CEnT1.2**	23.4	3.0	1.8	0.3	64.9	2.3
**CEnT1.3**	62.2	0.5	1.4	0.2	94.9	1.1
**CEnT1.4**	74.5	1.3	1.7	0.5	99.1	1.7

Table S3: Data collection and refinement statistics

	**CEnT1.4**
PDB ascension number	9ENO
Wavelength (Å)	0.9763
Resolution range	51.91 - 1.73 (1.792 - 1.73)[a]
Space group	P 1 2_1 1
Unit cell dimensions<br>a, b, c, (Å)<br>α, β, γ (°)	 51.97, 73.92, 74.87<br>90, 92.85, 90
Total reflections	406927 (39029)
Unique reflections	59053 (5851)
Multiplicity	6.9 (6.7)
Completeness (%)	98.97 (92.25)
Mean I/sigma(I)	6.98 (0.41)
Wilson B-factor	24.07
R-merge	0.1659 (2.152)
R-meas	0.1795 (2.334)
R-pim	0.06792 (0.8932)
$CC_{1/2}$	0.993 (0.459)
CC*	0.998 (0.793)
Reflections used in refinement	58484 (5405)
Reflections used for R-free	2079 (179)
R-work	0.1812 (0.3077)
R-free[b]	0.2258 (0.3188)
$CC_{1/2}$	0.969 (0.743)
CC*	0.957 (0.602)
Protein residues	612
RMS (bonds)	0.007
RMS (angles)	0.96
Ramachandran favoured (%)	96.01
Ramachandran allowed (%)	3.99
Ramachandran outliers (%)	0.00
Rotamer outliers (%)	0.58
Clashscore	3.20
Average B-factor	28.49

[a]Statistics for the highest-resolution shell are shown in parentheses.

[b]R-free was calculated using ~5% of the data separate from the rest.

Table S4: Population analysis of MD simulations of the CEnT1.4 – substrate complex for substrates 1 and 5.

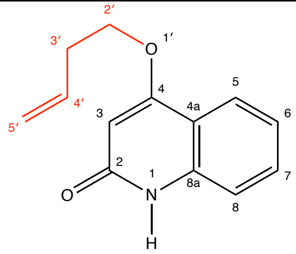 Substrate **1**		Product	Population[a] %	Population[b] %
θ(5-4a-4-4′) > 0	*R*(4′-4) < *R*(4′-3)	(-)-**1a**	5.0	3.1
	R(4′-4) > *R*(4′-3)	(4*S*, 4a*R*, 10b*R*)-**1b**	0.1	0.1
θ(5-4a-4-4′) < 0	*R*(4′-4) < *R*(4′-3)	(+)-**1a**	88.9	85.2
	R(4′-4) > *R*(4′-3)	(4*R*, 4a*S*, 10b*S*)-**1b**	6.0	11.6
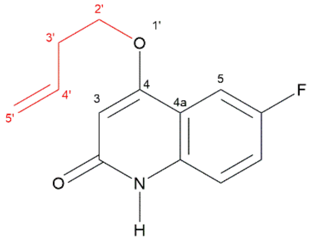 Substrate **5**		Product	Population[a] %	Population[b] %
θ(5-4a-4-4′) > 0	*R*(4′-4) < *R*(4′-3)	(3a*S*, 4a*R*, 10b*R*)-**5a**	1.5	2.4
	R(4′-4) > *R*(4′-3)	(3a*S*, 4a*R*, 10b*R*)-**5b**	0.3	0.6
θ(5-4a-4-4′) < 0	*R*(4′-4) < *R*(4′-3)	(3a*R*, 4a*S*, 10b*S*)-**5a**	92.0	86.2
	R(4′-4) > *R*(4′-3)	(3a*R*, 4a*S*, 10b*S*)-**5b**	6.2	10.8

[a] All data. [b]. Recalculated from the sample where *R*(4′ - 4) < 3.5 Å. Both datasets are shown in Figure S13.

Table S5: Mass spectrometry values for CEnT1.0 and variants.

Variant	Expect Mass	Observed Mass
EnT1.0	36193	36193.2
CEnT1.0	36438	36438.2
CEnT1.1	36424	36424.8
CEnT1.2	36398	36398.0
CEnT1.3	36411	36411.1
CEnT1.4	36408	36405.9

Table S6: List of primer sequences used in this study.

**Flanking Primers**	
**XHO_R**	ATGCATGCCTCGAGCGATCCGA
**NDE_F**	CATGCATGCATATGGAGATCCCTGTCA
**BpA Reposition**	
**B173A_F**	GATACCGACTTTCAGGCACCAGCTGGT
**B173W_F**	GATACCGACTTTCAGTGGCCAGCTGGT
**W244B**	TTGTTAGTTGCCAATTAGGGCTCCTCGCATATC
**173_R**	CTGAAATGCGGTATCCAC
**244_R**	ATTGGCAACTAACAAATTGTTG
**Rational Design**	
**Q149D_F**	TTTACCATCTCGTTAGATGAAAAATTCGGGTCAATCTATTG
**149_R**	TAACGAGATGGTAAA
**Round 1**	
**A21X_F**	ATTCCTGGTGCTNNKGGTCCAGTGTTCGAC
**21_R**	AGCACCAGGAATATCTTC
**Y37X_F**	TATATTGTGGCTCCANNKGTGGAGGTAAACGGTAAA
**37_R**	TGGAGCCACAATATAAAAGTC
**A74X_F**	GGAGGCATACCANNKGGGTGCCAGTGC
**74_R**	TGGTATGCCTCCATAGC
**M90X_F**	TTATTTGTAGCTGATNNKCGTCTGGGCCTTCT
**90_R**	ATCAGCTACAAATAACTGGTTG
**A120X_F**	ATGCAGGGTTGCNNKTACTGCGCTTTTGATTATGAA
**120_R**	CGTATGCAGGGTTGC
**Y121X_F**	CAGGGTTGCGCCNNKTGCGCTTTTGATTATGAAG
**121_R**	GGCGCAACCCTG
**P135X_F**	TGGATTACGGCCNNKGCGGGCGAGGT
**135_R**	GGCCGTAATCCATAAGTT
**I146X_F**	GCCGATTTTACCNNKTCGTTACAAGAAAAATTCGGG
**146_R**	AAAATCGGCAGGGG
**L148X_F**	TTTACCATCTCGNNKCAAGAAAAATTCGGGTCAATC
**148_R**	CGAGATGGTAAAATCGGC
**D149X_F**	TTTACCATCTCGTTANNKGAAAAATTCGGGTCAATCTATTG
**149_R**	TAACGAGATGGTAAA
**A173X_F**	ACCGCATTTCAGNNKCCAGCTGGTATTGCC
**173_R**	CTGAAATGCGGTATCCAC
**A175X_F**	TTTCAGGCGCCANNKGCTGGTATTGCCGTG
**175_R**	CTGAAATGCGGTATCCAC
**P196X_F**	ATTGTGGCCGAGCAGNNKACTAAAAAGTTATGGTCCTATGAC
**196_R**	CTGCTCGGCCACAAT
**K225X_F**	CCGGGCACTCACNNKGGTGGAGCCGC
**225_R**	GTGAGTGCCCGG
**G227X_F**	ACTCACAAAGGTNNKGCCGCCGGTATG
**227_R**	ACCTTTGTGAGTGCC
**A229X_F**	AAAGGTGGAGCCNNKGGTATGGATTTCGACGAAG

**229_R**	CACAAAGGTGGAGCC
**A242X_F**	AATTTGTTAGTTNNKGCCAATTAGGGCTCC
**242_R**	AACTAACAAATTGTTGTCTTCGT
**P254X_F**	GAAGTATTCGGCNNKGATGGAGGTCAACCTAAGA
**254_R**	GCATATCGAAGTATTCGGC
**K269X_F**	TGCCCGTTCGAANNKCCTTCTGCTTTACACTTTAAG
**269_R**	TTCGAACGGGCAG
**S271X_F**	CGTTCGAAAAACCTNNKGCTTTACACTTTAAGCCACA
**271_R**	AGGTTTTTCGAACGGG
**H287X_F**	ATTTTCGTGACGGAGNNKGAAAACAATGCAGTCTGGAA
**287_R**	CTCCGTCACGAAAAT
**N290X_F**	GACGGAGCACGAANNKGCAGTCTGGAAGTTCG
**290_R**	CGGAGCACGAAAACTC
	**Round 2**
**A21X_F**	GATATTCCTGGTGCTNNKGGTCCAGTGTTCGAC
**21_R**	AGCACCAGGAATATCTTC
**Y37X_F**	TATATTGTGGCTCCANNKGTGGAGGTAAACGGTAAA
**37_R**	TGGAGCCACAATATAAAAGTC
**I72X_F**	GTCAACGGCTATGGAGGCNNKCCAGCCGGGTG
**72_R**	GCCTCCATAGCCG
**A74X_F**	TATGGAGGCATACCANNKGGGTGCCAGTGC
**74_R**	TGGTATGCCTCCATAGC
**M90X_F**	TTATTTGTAGCTGATNNKCGTCTGGGCCTTCT
**90_R**	ATCAGCTACAAATAACTGG
**A120X_F**	CGTATGCAGGGTTGCNNKTACTGCGCTTTTGATTATGAA
**120_R**	CGTATGCAGGGTTGC
**Y121X_F**	ATGCAGGGTTGCGCCNNKTGCGCTTTTGATTATGAAG
**121_R**	GGCGCAACCCTG
**P135X_F**	TTATGGATTACGGCCNNKGCGGGCGAGGT
**135_R**	GGCCGTAATCCATAAGTT
**F144X_F**	GAGGTGGCCCCTGCCGATNNKACCATCTCGGTAGACG
**144_R**	ATCGGCAGGGGC
**I146X_F**	CCCTGCCGATTTTACCNNKTCGGTACAAGAAAAATTCGG
**146_R**	AAAATCGGCAGGGG
**Q172X_F**	GTGGATACCGCATTTNNKGCGCCAGCTGG
**172_R**	CAAGTGGATACCGCATTT
**A173X_F**	GATACCGCATTTCAGNNKCCAGCTGGTATTGCC
**173_R**	CTGAAATGCGGTATCCAC
**Q195X_F**	CTGATTGTGGCCGAGNNKCCAACTAAAAAGTTATGGTCC
**195_R**	CTCGGCCACAATCAG
**P196X_F**	ATTGTGGCCGAGCAGNNKACTAAAAAGTTATGGTCCTATGAC
**196_R**	CTGCTCGGCCAC
**A229X_F**	CACGTAGGTGGAGCCNNKGGTATGGATTTCGACGAAG
**229_R**	GGCTCCACCTACGT
**A242X_F**	AACAATTTGTTAGTTNNKGCCAATTAGGGCTCC
**242_R**	AACTAACAAATTGTTGTCTTCGT

**K269X_F**	CGCTGCCCGTTCGAANNKCCTTGCGCTTTACACTTT
**269_R**	TTCGAACGGGCAG
**A272X_F**	TTCGAAAAACCTTGCNNKTTACACTTTAAGCCACAGAC
**272_R**	GCAAGGTTTTTCGAACG
**H287X_F**	ATTTTCGTGACGGAGNNKGAAAACAATGCAGTCTGGAA
**287_R**	CTCCGTCACGAAAATGG
**Round 3**	
**V34X_F**	GACAAGAATGGGGACTTTTATATTNNKGCTCCATTTGTGGAGG
**34_R**	AATATAAAAGTCCCCATTCTTGTC
**P36X_F**	GGGACTTTTATATTGTGGCTNNKTTTGTGGAGGTAAACGG
**36_R**	AGCCACAATATAAAAGTCCC
**I72X_F**	ACGGCTATGGAGGCNNKCCAGCCGGGTG
**72_R**	GCCTCCATAGCCGT
**A74X_F**	GCTATGGAGGCATACCANNKGGGTGCCAGTGC
**74_R**	GCTATGGAGGCATACCA
**M90X_F**	CAACCAGTTATTTGTAGCTGATNNKCGTCTGGGCCTTCT
**90_R**	ATCAGCTACAAATAACTGGTTG
**A136X_F**	GGATTACGGCCCCANNKGGCGAGGTGGC
**136_R**	TGGGGCCGTAATCC
**F144X_F**	GTGGCCCCTGCCGATNNKACCATCTCGGTTGATGAA
**144_R**	ATCGGCAGGGGC
**T145X_F**	GCCCCTGCCGATTTTNNKATCTCGGTTGATGAAAAATTC
**145_R**	AAAATCGGCAGGGGC
**I146X_F**	CCCTGCCGATTTTACCNNKTCGGTTGATGAAAAATTCGG
**146_R**	GGTAAAATCGGCAGGG
**S147X_F**	CCTGCCGATTTTACCATCNNKGTTGATGAAAAATTCGGGTC
**147_R**	GATGGTAAAATCGGCAGG
**V148X_F**	GCCGATTTTACCATCTCGNNKGATGAAAAATTCGGGTCAATC
**148_R**	CGAGATGGTAAAATCGGC
**D149X_F**	CGATTTTACCATCTCGGTTNNKGAAAAATTCGGGTCAATCTATTG
**149_R**	AACCGAGATGGTAAAATCG
**Q172X_F**	CAAGTGGATACCGCATTTNNKGCGCCAGCTGGT
**172_R**	AAATGCGGTATCCACTTG
**A173X_F**	GTGGATACCGCATTTCAGNNKCCAGCTGGTATTGCC
**173_R**	CTGAAATGCGGTATCCAC
**A175X_F**	GCATTTCAGGCGCCANNKGGTATTGCCGTGCG
**175_R**	TGGCGCCTGAAATGC
**Q195X_F**	CTGATTGTGGCCGAGNNKCCAACTAAAAAGTTATGGTCCT
**195_R**	CTCGGCCACAATCAG
**P196X_F**	ATTGTGGCCGAGCAGNNKACTAAAAAGTTATGGTCCTATGAC
**196_R**	CTGCTCGGCCACA
**G227X_F**	GGCACTCACGTGGGTNNKGCCGCCGGTATG
**227_R**	ACCCACGTGAGTGCC
**A229X_F**	CACGTGGGTGGAGCCNNKGGTATGGATTTCGACGAAG
**229_R**	GGCTCCACCCACG
**V271X_F**	CCCGTTCGAAAAACCTNNKGCTTTACACTTTAAGCCAC

**271_R**	AGGTTTTTCGAACGGG
**A272_F**	CCGTTCGAAAAACCTTGTNNKTTACACTTTAAGCCACAGAC
**272_R**	ACAAGGTTTTTCGAACGG
**T285_F**	CAGACAAAAACCATTTTCGTGNNKGAGCACGAAAACAATGCA
**285_R**	CACGAAAATGGTTTTTGTCTG
**H287_F**	CCATTTTCGTGACGGAGNNKGAAAACAATGCAGTCTGGAA
**287_R**	CTCCGTCACGAAAATGG
**Primers to introduce point mutations**	
**Q195A_F**	CTGATTGTGGCCGAGGCTCCAACTAAAAAGTTATGGTCCT
**195_R**	CTCGGCCACAATCAG
**T21A_F**	GAAGATATTCCTGGTGCTGCTGGTCCAGTGTTCGAC
**21_R**	AGCACCAGGAATATCTTC
**Y121F_F**	ATGCAGGGTTGCGCCTTTTGCGCTTTTGATTATGAAG
**121_R**	GGCGCAACCCTGCAT
	**Round 4 (substrate 2)**
**A20_F**	GAAGATATTCCTGGTNNKACTGGTCCAGTGTTC
**G22_F**	ATTCCTGGTGCTACTNNKCCAGTGTTCGACAAGAAT
**P36_F**	TTTTATATTGTGGCTNNKTTTGTGGAGGTAAACGG
**V38_F**	ATTGTGGCTCCATTTNNKGAGGTAAACGGTAAACCA
**I72_F**	AACGGCTATGGAGGCNNKCCAGCCGGGTG
**P73_F**	GGCTATGGAGGCATANNKGCCGGGTGCC
**A74_F**	TATGGAGGCATACCANNKGGGTGCCAGTGC
**M90_F**	TTATTTGTAGCTGATNNKCGTCTGGGCCTTC
**A120_F**	CGTATGCAGGGTTGCNNKTACTGCGCTTTTGATTATGA
**Y121_F**	ATGCAGGGTTGCGCCNNKTGCGCTTTTGATTATGAAG
**P135_F**	TTATGGATTACGGCCNNKGCGGGCGAGG
**L146_F**	CCTGCCGATTTTACCNNKTCGGTTGATGAAAAATTCG
**S147_F**	GCCGATTTTACCATCNNKGTTGATGAAAAATTCGGGTC
**P174_F**	ACCGCATTTCAGGCGNNKGCTGGTATTGCCGT
**A175_F**	GCATTTCAGGCGCCANNKGGTATTGCCGTGCG
**A228_F**	ACTCACGTGGGTGGANNKGCCGGTATGGATTTCG
**A229_F**	CACGTGGGTGGAGCCNNKGGTATGGATTTCGACGAA
**P270_F**	TGCCCGTTCGAAAAANNKGTGGGTTTACACTTTAAGC
**E286_F**	ACCATTTTCGTGACGNNKCACGAAAACAATGCAGTC
**E288_F**	TTCGTGACGGAGCACNNKAACAATGCAGTCTGGAAG
**A20_R**	ACCAGGAATATCTTCGGT
**G22_R**	AGTAGCACCAGGAATATCT
**P36_R**	AGCCACAATATAAAAGTCCC
**V38_R**	AAATGGAGCCACAATATAAAAGT
**I72_R**	GCCTCCATAGCCG
**P73_R**	TATGCCTCCATAGCC
**A74_R**	TGGTATGCCTCCATAG
**M90_R**	ATCAGCTACAAATAACTGGTT
**A120_R**	GCAACCCTGCATACG
**Y121_R**	GGCGCAACCCTG
**P135_R**	GGCCGTAATCCATAAGTTT

**L146_R**	GGTAAAATCGGCAGGG
**S147_R**	GATGGTAAAATCGGCAGG
**P174_R**	CGCCTGAAATGCG
**A175_R**	TGGCGCCTGAAATG
**A228_R**	TCCACCCACGTGAG
**A229_R**	GGCTCCACCCAC
**P270_R**	TTTTTCGAACGGGCA
**E286_R**	CGTCACGAAAATGGTTTTT
**E288_R**	GTGCTCCGTCACG

Table S7: Charges used in the MD parameter (prep) file for BpA

Atom Name	Atom Type	Atom Charge
N1	N	-0.4175
H13	H	0.2719
C2	CT	0.128123
C4	CT	-0.24154
C5	CA	0.169547
C6	CA	-0.23294
C8	CA	-0.08319
C10	CA	-0.21154
C9	CA	-0.08319
C7	CA	-0.23294
H4	HA	0.169127
H6	HA	0.12735
C16	C	0.737119
O3	O	-0.52717
C17	CA	-0.25571
C11	CA	-0.03232
C13	CA	-0.17734
C15	CA	-0.08451
C14	CA	-0.17734
C12	CA	-0.03232
H8	HA	0.114657
H10	HA	0.139864
H12	HA	0.132281
H9	HA	0.139864
H7	HA	0.114657
H5	HA	0.12735
H3	HA	0.169127
H1	HC	0.11346
H2	HC	0.11346
H11	H1	-0.00775
C3	C	0.5973
O2	O	-0.5679

Table S8: Charges used in the MD parameter (prep) file for product (+)-**1**.

Atom Name	Atom Type	Atom Charge
C8	cy	0.198100
C10	cy	-0.099700
C12	c3	0.123400
C13	c	0.717500
C4	ca	0.094600
C3	ca	-0.187000
C2	ca	-0.087000
C1	ca	-0.168000
C6	ca	-0.066000
C5	ca	-0.124300
C7	cy	-0.162700
C9	cy	-0.066400
C11	c3	-0.101400
N1	n	-0.468100
O1	o	-0.626500
O2	os	-0.400600
H9	hc	0.084700
H8	h1	0.049700
H12	h1	0.049700
H3	ha	0.136000
H2	ha	0.135000
H1	ha	0.136000
H4	ha	0.142000
H5	hc	0.110700
H6	hc	0.066200
H7	hc	0.066200
H10	hc	0.058200
H11	hc	0.058200
H13	hn	0.329500

Table S9: Charges used in the MD parameter (prep) file for substrate **1**.

Atom Name	Atom Type	Atom Charge
C3	ca	-0.379461
H3	ha	0.188796
C2	ca	-0.035920
H2	ha	0.147000
C1	ca	-0.264916
H1	ha	0.161944
C6	ca	-0.023903
H4	ha	0.124020
C5	ca	-0.223546
C4	ca	0.458151
N1	n	-0.699696
H5	hn	0.389016
C7	c	0.834608
O1	o	-0.662298
C8	cc	-0.615865
H6	ha	0.194791
C9	cd	0.504662
O2	os	-0.411154
C10	c3	0.166969
H7	h1	0.058137
H8	h1	0.058137
C11	c3	-0.023728
H9	hc	0.069736
H10	hc	0.069736
C12	c2	-0.170133
H11	ha	0.142598
C13	c2	-0.448385
H12	ha	0.195353
H13	ha	0.195353

Table S10: Analytical UPLC methods for the products 1a-9a and 1b-8b. Column temperature was set to 20°C, and detector wavelength was set to 260nm.

Substrate	Flow (mL^{-1})	Mobile Phase (%MeCN in H_2O)	Run Time (minutes)	Extinction Coefficient (mM^{-1} cm^{-1})	
				Substrate	Product(s)
**1**	1.2	5-50% (2.0 min)<br>5% (2.1-2.5 min)	2.5	747 (**1**)	718 (**1a**)<br>693 (**1b**)
**2**	1.25	27% (1.0 min)<br>27-55% (1.0-1.75 min)<br>27% (1.9-2.5 min)	2.5	283 (**2**)	496 (**2a**)<br>488 (**2b**)
**3**	1.0	25-40% (2.5 min)<br>40% (2.5-3.0 min)<br>25% (3.1-4.0 min)	4.0	800 (**3**)	883 (**3a**)<br>491 (**3b**)
**4**	1.0	25-40% (2.5 min)<br>40% (2.5-3.0 min)<br>25% (3.1-4.0 min)	4.0	313 (**4**)	1105 (**4a**)<br>876 (**4b**)
**5**	1.0	15-40% (3.0 min)<br>15% (3.1-3.75 min)	3.75	481 (**5**)	1077 (**5a**)<br>581 (**5b**)
**6**	1.0	15-40% (3.0 min)<br>15% (3.1-3.75 min)	3.75	413 (**6**)	1323 (**6a**)<br>1104 (**6b**)
**7**	1.0	18-40% (3.0 min)<br>18% (3.1-3.75 min)	3.75	542 (**7**)	1475 (**7a**)<br>398 (**7b**)
**8**	1.0	12-38% (3.0 min)<br>12% (3.1-3.75 min)	3.75	482 (**8**)	622 (**8a**)<br>182 (**8b**)
**9**	1.0	15-40% (3.0 min)<br>15% (3.1-3.75 min)	3.75	333 (**9**)	610 (**9a**)

Table S11: Chiral analytical methods for the products 1a-9a and 1b-8b.

SFC chiral analytical methods						
**Substrate**	**Flow (mL^{-1})**	**Mobile Phase (%MeOH in CO_2)**	**Run Time (minutes)**	**Products(s)**	**Column Temperature (°C)**	**Column Identity**
**1**	1	24	12	(**1a**)<br>(**1b**)	15	IG-3
**2**	1	3-15% 15 minutes<br>3% 1 minute	16	(**2a**)<br>(**2b**)	10	IG-3
**4**	0.8	15	8	(**4a**)<br>(**4b**)	20	IG-3
**9**	0.8	15	5	(**9a**)	20	IC-3
**UPLC chiral analytical methods**						
**Substrate**	**Flow (mL^{-1})**	**Mobile Phase (%IPA in Hexane)**	**Run Time (minutes)**	**Products(s)**	**Column Temperature (°C)**	**Column Identity**
**3**	0.75	6%	6	(**3a**)<br>(**3b**)	20	IG-3
**5**	0.4	10%	6	(**5a**)<br>(**5b**)	15	IG-3
**6**	0.2	12%	5	(**6a**)<br>(**6b**)	15	IB N-3
**7**	0.2	12%	6	(**7a**)<br>(**7b**)	15	IB N-3
**8**	0.2	15%	10	(**8a**)<br>**(8b)**	15	IB N-3

Table S12: Substrate scope of CEnT variants. Reaction conditions for the synthesis of **1a-9a** and **1b-8b** conversion is displayed as a total of major and minor product where appropriate. All reactions were performed using PBS buffer pH 7.4 and 5% DMSO in triplicate. Standard deviations are given for measurements in triplicate. Reaction conversions are determined based on UPLC calibration curves of authentic standards of starting materials and products. Extinction coefficients and UPLC methods are given in Table S10. The *e.e.* values are determined using chiral UPLC analysis (methods in Table S11). Chromatograms of substrate standards, product standards, and biotransformations are provided at the end of the Supporting Information.

Substrate	Product(s)	Variant	Catalyst Loading (mol%)	Substrate Loading (mM)	Time (minutes)	Conversion (%)	S.D.	*e.e.* (%)
1	1a + 1b	CEnT1.4	5	0.4	90	98		99
2[†]	2a + 2b	CEnT1.4b	10	0.75	90	92	0.47	81
3	3a + 3b	CEnT1.3	10	0.30	90	84	0.54	78
4	4a + 4b	CEnT1.3_C271V_A272G	7.5	0.30	45	81	1.88	86
5[†]	5a + 5b	CEnT1.3_C271V_A272G	7.5	0.30	120	89	1.25	90
6	6a + 6b	CEnT1.3	10	0.30	45	84	1.38	84
7	7a + 7b	CEnT1.3_Y121A_C271V _A272G	10	0.40	20	86	4.92	85
8	8a + 8b	CEnT1.3_C271V_A272G	7.5	0.40	90	98	0.22	90
9	9a	CEnT1.3_C271V_A272G	10	0.40	60	83	0.98	89

[†] LED intensity was set to 50% to minimise background reactions.

Protein and DNA sequences:
B = 4-benzoylphenylalanine, BpA

CEnT1.0

ATGGGCAGCAGCCATCATCATCATCATCACAGCAGCGGCCTGGTGCCGCGCGGCAGCCATAT
GGAGATCCCTGTCATTGAGCCTTTGTTTACCAAAGTGACCGAAGATATTCCTGGTGCTGCTG
GTCCAGTGTTCGACAAGAATGGGGACTTTTATATTGTGGCTCCATACGTGGAGGTAAACGGT
AAACCAGCAGGTGAAATCTTGCGCATCGATTTGAAGACAGGTAAGAAGACGGTCATCTGTAA
GCCTGAAGTCAACGGCTATGGAGGCATACCAGCCGGGTGCCAGTGCGACCGTGACGCCAACC
AGTTATTTGTAGCTGATATGCGTCTGGGCCTTCTTGTTGTGCAAACTGATGGGACATTTGAA
GAAATTGCAAAAAAGGACAGCGAGGGGCGTCGTATGCAGGGTTGCGCCTACTGCGCTTTTGA
TTATGAAGGAAACTTATGGATTACGGCCCCAGCGGGCGAGGTGGCCCCTGCCGATTTTACCA
TCTCGTTACAGGAAAAATTCGGGTCAATCTATTGTTTTACCACCGACGGGCAAATGATCCAA
GTGGATACCGCATTTCAGGCGCCAGCTGGTATTGCCGTGCGCCACATGAACGACGGTCGCCC
ATATCAACTGATTGTGGCCGAGCAGCCAACTAAAAAGTTATGGTCCTATGACATTAAAGGAC
CGGCAAAAATTGAAAATAAGAAAGTGTGGGGGCACATCCCGGGCACTCACAAAGGTGGAGCC
GCCGGTATGGATTTCGACGAAGACAACAATTTGTTAGTTGCCAATTAGGGCTCCTCGCATAT
CGAAGTATTCGGCCCGGATGGAGGTCAACCTAAGATGCGCATCCGCTGCCCGTTCGAAAAAC
CTTCGGCTTTACACTTTAAGCCACAGACAAAAACCATTTTCGTGACGGAGCACGAAAACAAT
GCAGTCTGGAAGTTCGAGTGGCAGCGCAATGGCAAAAAGCAGTATTGCGAAACTCCTAAGTG
A

MGSSHHHHHHSSGLVPRGSHMEIPVIEPLFTKVTEDIPGAAGPVFDKNGDFYIVAPYV
EVNGKPAGEILRIDLKTGKKTVICKPEVNGYGGIPAGCQCDRDANQLFVADMRLGLL
VVQTDGTFEEIAKKDSEGRRMQGCAYCAFDYEGNLWITAPAGEVAPADFTISLQEKF
GSIYCFTTDGQMIQVDTAFQAPAGIAVRHMNDGRPYQLIVAEQPTKKLWSYDIKGPA
KIENKKVWGHIPGTHKGGAAGMDFDEDNNLLVANBGSSHIEVFGPDGGQPKMRIRC
PFEKPSALHFKPQTKTIFVTEHENNAVWKFEWQRNGKKQYCETPK

CEnT1.1

ATGGGCAGCAGCCATCATCATCATCATCACAGCAGCGGCCTGGTGCCGCGCGGCAGCCATAT
GGAGATCCCTGTCATTGAGCCTTTGTTTACCAAAGTGACCGAAGATATTCCTGGTGCTGCTG
GTCCAGTGTTCGACAAGAATGGGGACTTTTATATTGTGGCTCCATACGTGGAGGTAAACGGT
AAACCAGCAGGTGAAATCTTGCGCATCGATTTGAAGACAGGTAAGAAGACGGTCATCTGTAA
GCCTGAAGTCAACGGCTATGGAGGCATACCAGCCGGGTGCCAGTGCGACCGTGACGCCAACC
AGTTATTTGTAGCTGATATGCGTCTGGGCCTTCTTGTTGTGCAAACTGATGGGACATTTGAA
GAAATTGCAAAAAAGGACAGCGAGGGGCGTCGTATGCAGGGTTGCGCCTACTGCGCTTTTGA
TTATGAAGGAAACTTATGGATTACGGCCCCAGCGGGCGAGGTGGCCCCTGCCGATTTTACCA
TCTCGTTAGATGAAAAATTCGGGTCAATCTATTGTTTTACCACCGACGGGCAAATGATCCAA
GTGGATACCGCATTTCAGGCGCCAGCTGGTATTGCCGTGCGCCACATGAACGACGGTCGCCC
ATATCAACTGATTGTGGCCGAGCAGCCAACTAAAAAGTTATGGTCCTATGACATTAAAGGAC
CGGCAAAAATTGAAAATAAGAAAGTGTGGGGGCACATCCCGGGCACTCACAAAGGTGGAGCC
GCCGGTATGGATTTCGACGAAGACAACAATTTGTTAGTTGCCAATTAGGGCTCCTCGCATAT
CGAAGTATTCGGCCCGGATGGAGGTCAACCTAAGATGCGCATCCGCTGCCCGTTCGAAAAAC
CTTCGGCTTTACACTTTAAGCCACAGACAAAAACCATTTTCGTGACGGAGCACGAAAACAAT
GCAGTCTGGAAGTTCGAGTGGCAGCGCAATGGCAAAAAGCAGTATTGCGAAACTCCTAAGTG
A

MGSSHHHHHHSSGLVPRGSHMEIPVIEPLFTKVTEDIPGAAGPVFDKNGDFYIVAPYV
EVNGKPAGEILRIDLKTGKKTVICKPEVNGYGGIPAGCQCDRDANQLFVADMRLGLL
VVQTDGTFEEIAKKDSEGRRMQGCAYCAFDYEGNLWITAPAGEVAPADFTISLDEKF

GSIYCFTTDGQMIQVDTAFQAPAGIAVRHMNDGRPYQLIVAEQPTKKLWSYDIKGPA
KIENKKVWGHIPGTHKGGAAGMDFDEDNNLLVANBGSSHIEVFGPDGGQPKMRIRC
PFEKPSALHFKPQTKTIFVTEHENNAVWKFEWQRNGKKQYCETLK

CEnT1.2

```
ATGGGCAGCAGCCATCATCATCATCATCACAGCAGCGGCCTGGTGCCGCGCGGCAGCCATAT
GGAGATCCCTGTCATTGAGCCTTTGTTTACCAAAGTGACCGAAGATATTCCTGGTGCTGCTG
GTCCAGTGTTCGACAAGAATGGGGACTTTTATATTGTGGCTCCATACGTGGAGGTAAACGGT
AAACCAGCAGGTGAAATCTTGCGCATCGATTTGAAGACAGGTAAGAAGACGGTCATCTGTAA
GCCTGAAGTCAACGGCTATGGAGGCATACCAGCCGGGTGCCAGTGCGACCGTGACGCCAACC
AGTTATTTGTAGCTGATATGCGTCTGGGCCTTCTTGTTGTGCAAACTGATGGGACATTTGAA
GAAATTGCAAAAAAGGACAGCGAGGGGCGTCGTATGCAGGGTTGCGCCTACTGCGCTTTTGA
TTATGAAGGAAACTTATGGATTACGGCCCCAGCGGGCGAGGTGGCCCCTGCCGATTTTACCA
TCTCGGTTGATGAAAAATTCGGGTCAATCTATTGTTTTACCACCGACGGGCAAATGATCCAA
GTGGATACCGCATTTCAGGCGCCAGCTGGTATTGCCGTGCGCCACATGAACGACGGTCGCCC
ATATCAACTGATTGTGGCCGAGCAGCCAACTAAAAAGTTATGGTCCTATGACATTAAAGGAC
CGGCAAAAATTGAAAATAAGAAAGTGTGGGGGCACATCCCGGGCACTCACGTGGGTGGAGCC
GCCGGTATGGATTTCGACGAAGACAACAATTTGTTAGTTGCCAATTAGGGCTCCTCGCATAT
CGAAGTATTCGGCCCGGATGGAGGTCAACCTAAGATGCGCATCCGCTGCCCGTTCGAAAAAC
CTTGCGCTTTACACTTTAAGCCACAGACAAAAACCATTTTCGTGACGGAGCACGAAAACAAT
GCAGTCTGGAAGTTCGAGTGGCAGCGCAATGGCAAAAAGCAGTATTGCGAAACTCCTAAGTG
A
```

MGSSHHHHHHSSGLVPRGSHMEIPVIEPLFTKVTEDIPGAAGPVFDKNGDFYIVAPYV
EVNGKPAGEILRIDLKTGKKTVICKPEVNGYGGIPAGCQCDRDANQLFVADMRLGLL
VVQTDGTFEEIAKKDSEGRRMQGCAYCAFDYEGNLWITAPAGEVAPADFTISVDEK
FGSIYCFTTDGQMIQVDTAFQAPAGIAVRHMNDGRPYQLIVAEQPTKKLWSYDIKGP
AKIENKKVWGHIPGTHVGGAAGMDFDEDNNLLVANBGSSHIEVFGPDGGQPKMRIR
CPFEKPCALHFKPQTKTIFVTEHENNAVWKFEWQRNGKKQYCETLK

CEnT1.3

```
ATGGGCAGCAGCCATCATCATCATCATCACAGCAGCGGCCTGGTGCCGCGCGGCAGCCATAT
GGAGATCCCTGTCATTGAGCCTTTGTTTACCAAAGTGACCGAAGATATTCCTGGTGCTACTG
GTCCAGTGTTCGACAAGAATGGGGACTTTTATATTGTGGCTCCATTTGTGGAGGTAAACGGT
AAACCAGCAGGTGAAATCTTGCGCATCGATTTGAAGACAGGTAAGAAGACGGTCATCTGTAA
GCCTGAAGTCAACGGCTATGGAGGCATACCAGCCGGGTGCCAGTGCGACCGTGACGCCAACC
AGTTATTTGTAGCTGATATGCGTCTGGGCCTTCTTGTTGTGCAAACTGATGGGACATTTGAA
GAAATTGCAAAAAAGGACAGCGAGGGGCGTCGTATGCAGGGTTGCGCCTACTGCGCTTTTGA
TTATGAAGGAAACTTATGGATTACGGCCCCAGCGGGCGAGGTGGCCCCTGCCGATTTTACCA
TCTCGGTTGATGAAAAATTCGGGTCAATCTATTGTTTTACCACCGACGGGCAAATGATCCAA
GTGGATACCGCATTTCAGGCGCCAGCTGGTATTGCCGTGCGCCACATGAACGACGGTCGCCC
ATATCAACTGATTGTGGCCGAGCAGCCAACTAAAAAGTTATGGTCCTATGACATTAAAGGAC
CGGCAAAAATTGAAAATAAGAAAGTGTGGGGGCACATCCCGGGCACTCACGTGGGTGGAGCC
GCCGGTATGGATTTCGACGAAGACAACAATTTGTTAGTTGCCAATTAGGGCTCCTCGCATAT
CGAAGTATTCGGCCCGGATGGAGGTCAACCTAAGATGCGCATCCGCTGCCCGTTCGAAAAAC
CTTGCGCTTTACACTTTAAGCCACAGACAAAAACCATTTTCGTGACGGAGCACGAAAACAAT
GCAGTCTGGAAGTTCGAGTGGCAGCGCAATGGCAAAAAGCAGTATTGCGAAACTCCTAAGTG
A
```

MGSSHHHHHHSSGLVPRGSHMEIPVIEPLFTKVTEDIPGATGPVFDKNGDFYIVAPFV
EVNGKPAGEILRIDLKTGKKTVICKPEVNGYGGIPAGCQCDRDANQLFVADMRLGLL

VVQTDGTFEEIAKKDSEGRRMQGCAYCAFDYEGNLWITAPAGEVAPADFTISVDEK
FGSIYCFTTDGQMIQVDTAFQAPAGIAVRHMNDGRPYQLIVAEQPTKKLWSYDIKGP
AKIENKKVWGHIPGTHVGGAAGMDFDEDNNLLVANBGSSHIEVFGPDGGQPKMRIR
CPFEKPCALHFKPQTKTIFVTEHENNAVWKFEWQRNGKKQYCETLK

CEnT1.4

ATGGGCAGCAGCCATCATCATCATCATCACAGCAGCGGCCTGGTGCCGCGCGGCAGCCATAT
GGAGATCCCTGTCATTGAGCCTTTGTTTACCAAAGTGACCGAAGATATTCCTGGTGCTACTG
GTCCAGTGTTCGACAAGAATGGGGACTTTTATATTGTGGCTCCATTTGTGGAGGTAAACGGT
AAACCAGCAGGTGAAATCTTGCGCATCGATTTGAAGACAGGTAAGAAGACGGTCATCTGTAA
GCCTGAAGTCAACGGCTATGGAGGCATACCAGCCGGGTGCCAGTGCGACCGTGACGCCAACC
AGTTATTTGTAGCTGATATGCGTCTGGGCCTTCTTGTTGTGCAAACTGATGGGACATTTGAA
GAAATTGCAAAAAAGGACAGCGAGGGGCGTCGTATGCAGGGTTGCGCCTACTGCGCTTTTGA
TTATGAAGGAAACTTATGGATTACGGCCCCAGCGGGCGAGGTGGCCCCTGCCGATTTTACCC
TGTCGGTTGATGAAAAATTCGGGTCAATCTATTGTTTTACCACCGACGGGCAAATGATCCAA
GTGGATACCGCATTTCAGGCGCCAGCTGGTATTGCCGTGCGCCACATGAACGACGGTCGCCC
ATATCAACTGATTGTGGCCGAGCAGCCAACTAAAAAGTTATGGTCCTATGACATTAAAGGAC
CGGCAAAAATTGAAAATAAGAAAGTGTGGGGGCACATCCCGGGCACTCACGTGGGTGGAGCC
GCCGGTATGGATTTCGACGAAGACAACAATTTGTTAGTTGCCAATTAGGGCTCCTCGCATAT
CGAAGTATTCGGCCCGGATGGAGGTCAACCTAAGATGCGCATCCGCTGCCCGTTCGAAAAAC
CTGTGGCTTTACACTTTAAGCCACAGACAAAAACCATTTTCGTGACGGAGCACGAAAACAAT
GCAGTCTGGAAGTTCGAGTGGCAGCGCAATGGCAAAAAGCAGTATTGCGAAACTCCTAAGTG
A

MGSSHHHHHHSSGLVPRGSHMEIPVIEPLFTKVTEDIPGATGPVFDKNGDFYIVAPFV
EVNGKPAGEILRIDLKTGKKTVICKPEVNGYGGIPAGCQCDRDANQLFVADMRLGLL
VVQTDGTFEEIAKKDSEGRRMQGCAYCAFDYEGNLWITAPAGEVAPADFTLSVDEK
FGSIYCFTTDGQMIQVDTAFQAPAGIAVRHMNDGRPYQLIVAEQPTKKLWSYDIKGP
AKIENKKVWGHIPGTHVGGAAGMDFDEDNNLLVANBGSSHIEVFGPDGGQPKMRIR
CPFEKPVALHFKPQTKTIFVTEHENNAVWKFEWQRNGKKQYCETLK

CEnT1.4b

ATGGGCAGCAGCCATCATCATCATCATCACAGCAGCGGCCTGGTGCCGCGCGGCAGCCATAT
GGAGATCCCTGTCATTGAGCCTTTGTTTACCAAAGTGACCGAAGATATTCCTGGTGCTACTG
GTCCAGTGTTCGACAAGAATGGGGACTTTTATATTGTGGCTCCATTTGTGGAGGTAAACGGT
AAACCAGCAGGTGAAATCTTGCGCATCGATTTGAAGACAGGTAAGAAGACGGTCATCTGTAA
GCCTGAAGTCAACGGCTATGGAGGCATACCAGCCGGGTGCCAGTGCGACCGTGACGCCAACC
AGTTATTTGTAGCTGATGCTCGTCTGGGCCTTCTTGTTGTGCAAACTGATGGGACATTTGAA
GAAATTGCAAAAAAGGACAGCGAGGGGCGTCGTATGCAGGGTTGCGCCTACTGCGCTTTTGA
TTATGAAGGAAACTTATGGATTACGGCCCCAGCGGGCGAGGTGGCCCCTGCCGATTTTACCA
TCTCGGTTGATGAAAAATTCGGGTCAATCTATTGTTTTACCACCGACGGGCAAATGATCCAA
GTGGATACCGCATTTCAGGCGCCAACTGGTATTGCCGTGCGCCACATGAACGACGGTCGCCC
ATATCAACTGATTGTGGCCGAGCAGCCAACTAAAAAGTTATGGTCCTATGACATTAAAGGAC
CGGCAAAAATTGAAAATAAGAAAGTGTGGGGGCACATCCCGGGCACTCACGTGGGTGGAGCC
TGTGGTATGGATTTCGACGAAGACAACAATTTGTTAGTTGCCAATTAGGGCTCCTCGCATAT
CGAAGTATTCGGCCCGGATGGAGGTCAACCTAAGATGCGCATCCGCTGCCCGTTCGAAAAAC
CTGTGGGTTTACACTTTAAGCCACAGACAAAAACCATTTTCGTGACGGAGCACGAAAACAAT
GCAGTCTGGAAGTTCGAGTGGCAGCGCAATGGCAAAAAGCAGTATTGCGAAACTCTTAAGTG
A

MGSSHHHHHHSSGLVPRGSHMEIPVIEPLFTKVTEDIPGATGPVFDKNGDFYIVAPFV
EVNGKPAGEILRIDLKTGKKTVICKPEVNGYGGIPAGCQCDRDANQLFVADARLGLL
VVQTDGTFEEIAKKDSEGRRMQGCAYCAFDYEGNLWITAPAGEVAPADFTISVDEK
FGSIYCFTTDGQMIQVDTAFQAPTGIAVRHMNDGRPYQLIVAEQPTKKLWSYDIKGP
AKIENKKVWGHIPGTHVGGACGMDFDEDNNLLVANBGSSHIEVFGPDGGQPKMRIR
CPFEKPVGLHFKPQTKTIFVTEHENNAVWKFEWQRNGKKQYCETLK

NMR Spectra

4-(but-3-en-1-yloxy)-6-methylquinolin-2(1H)-one (4)

^{1}H-NMR (400 MHz, $CDCl_3$, 298 K):

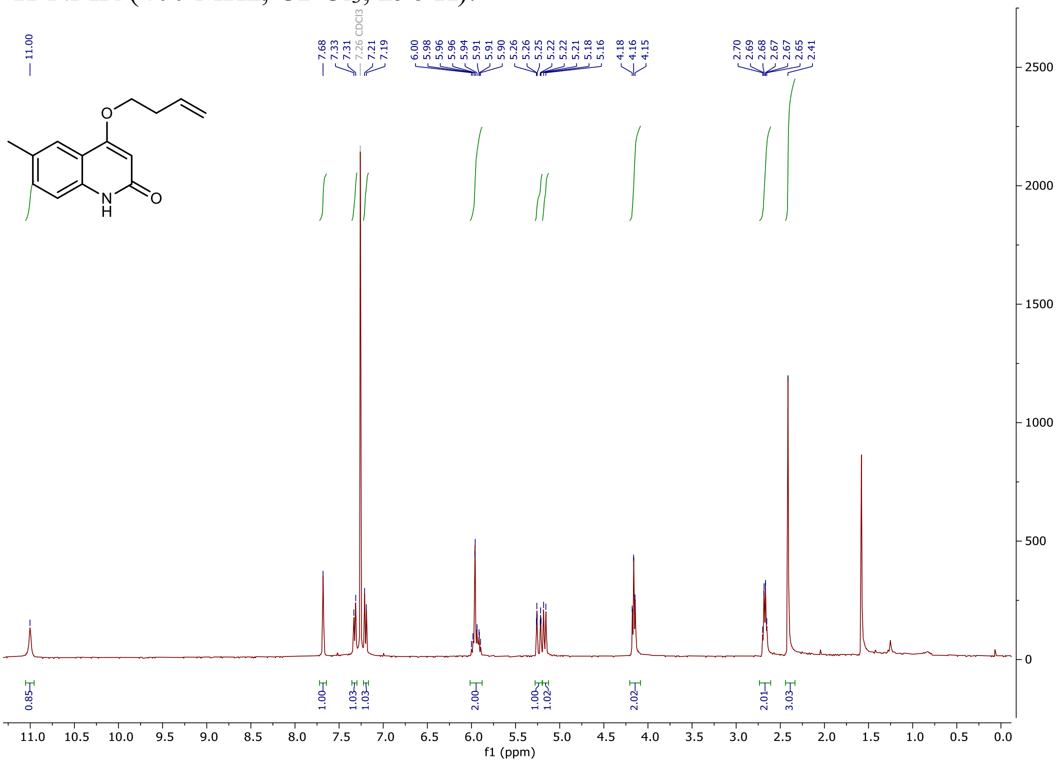

^{13}C-NMR (101 MHz, DMSO-d_6, 298K):

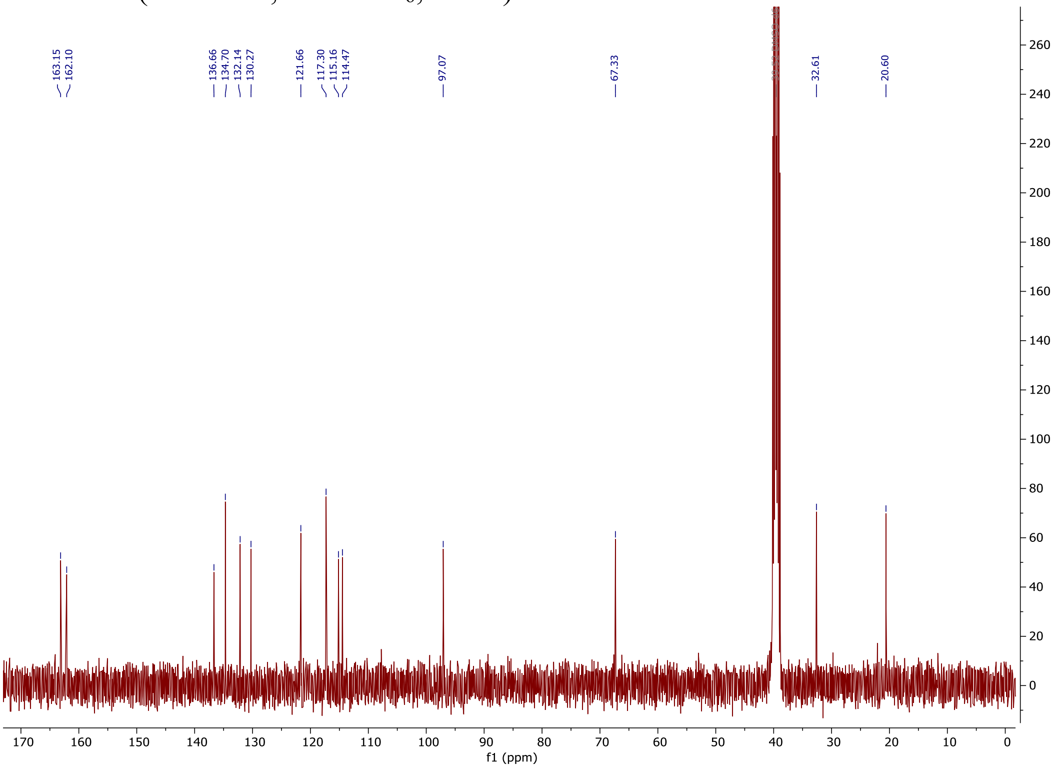

4-(but-3-en-1-yloxy)-6-fluoroquinolin-2(1H)-one (5)

^{1}H-NMR (500 MHz, $CDCl_3$, 298K):

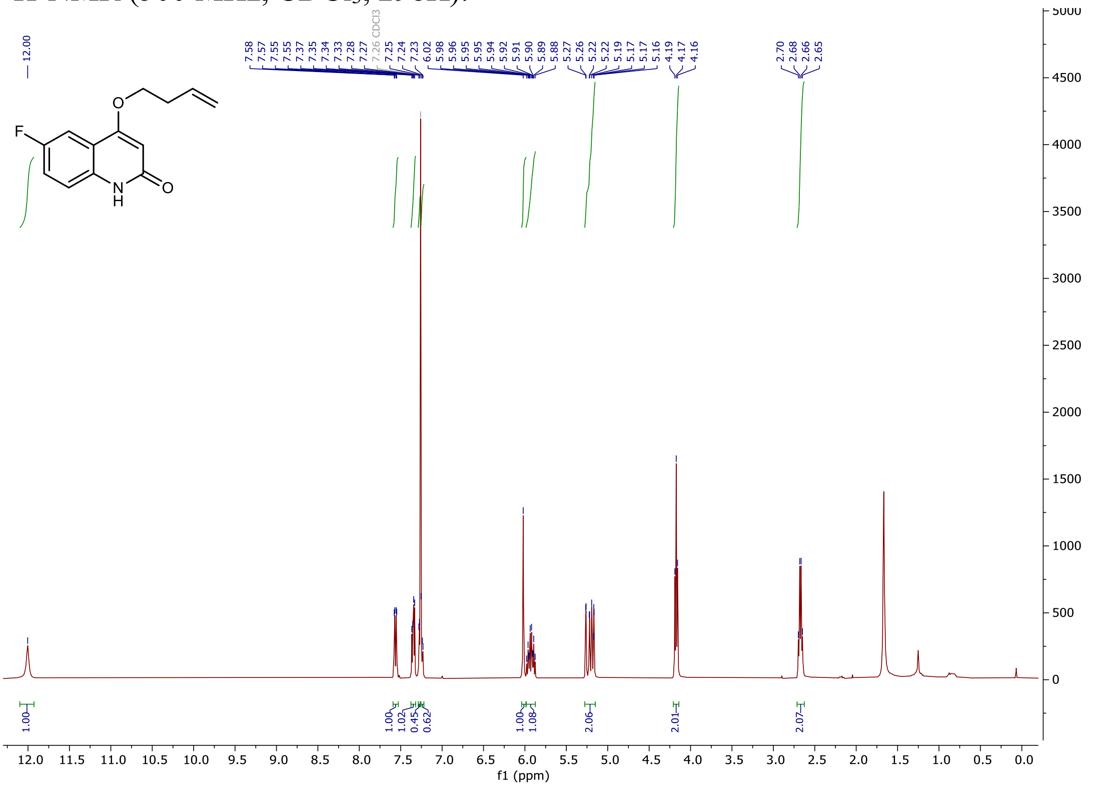

^{13}C-NMR (101 MHz, $CDCl_3$, 298K):

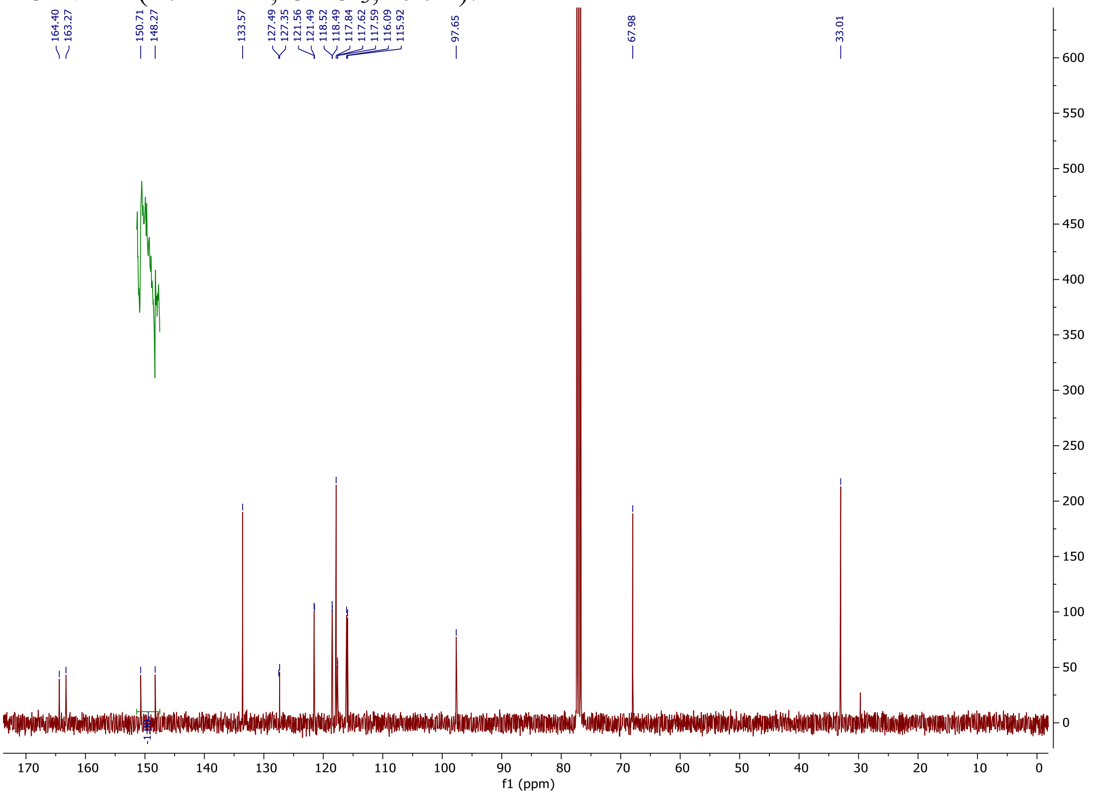

^{19}F NMR (376 MHz, $CDCl_3$, 298K)

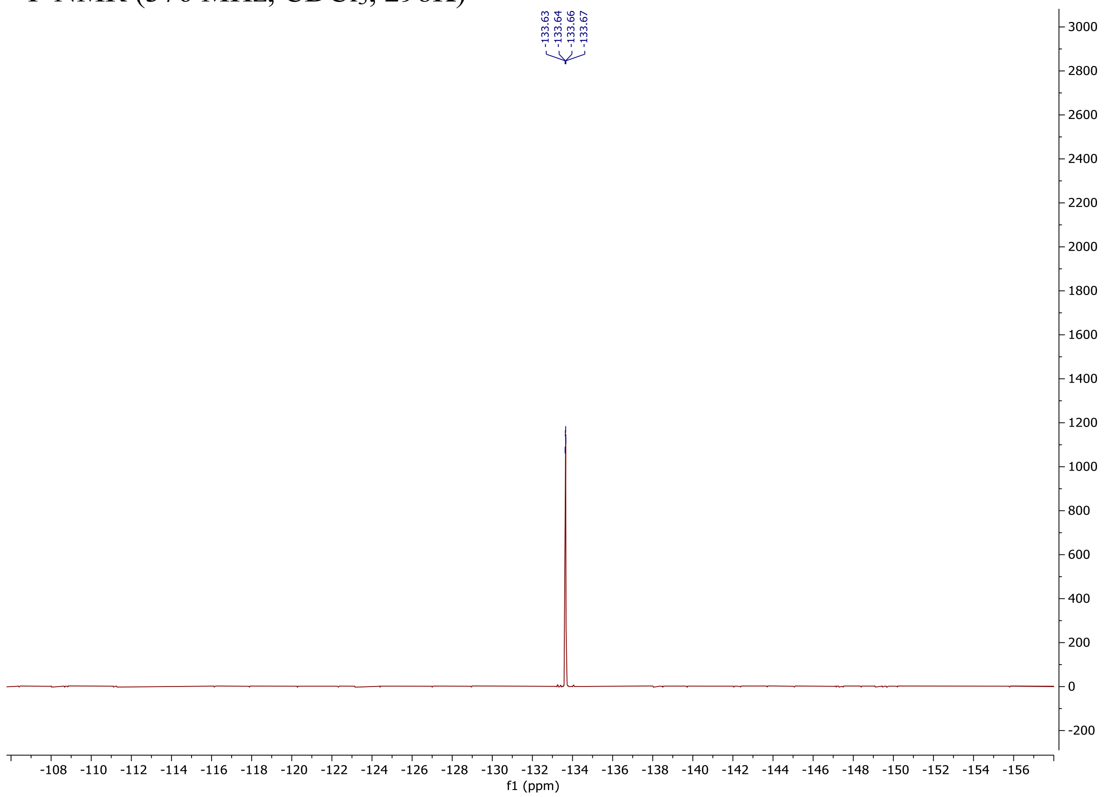

4-(but-3-en-1-yloxy)-6-chloroquinolin-2(1H)-one (6)

^{1}H-NMR (500 MHz, $CDCl_3$, 298K):

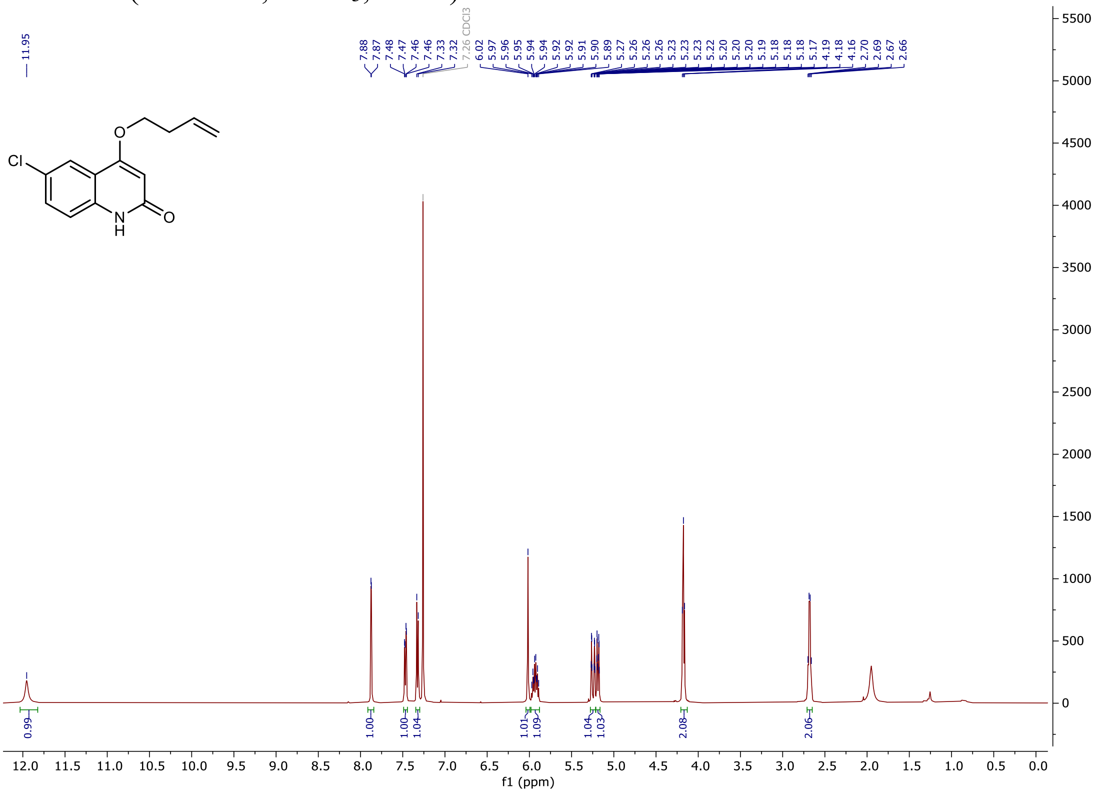

^{13}C-NMR (101 MHz, DMSO-d_6, 298K):

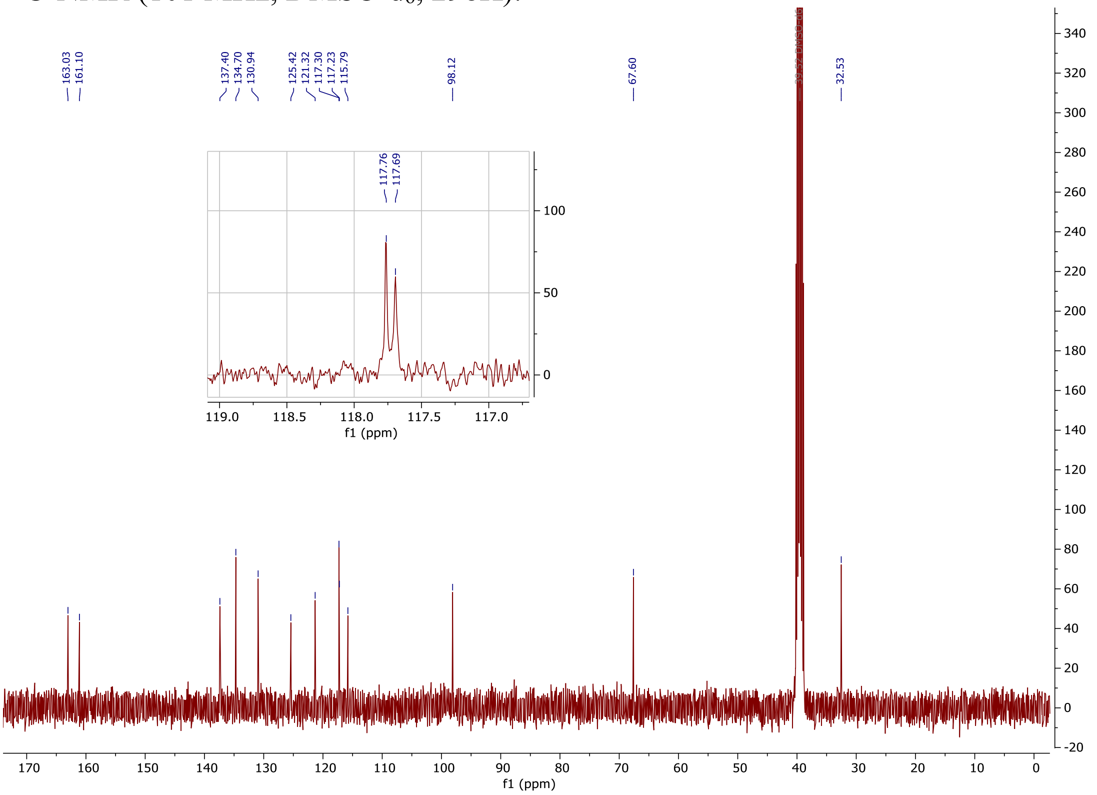

6-bromo-4-(but-3-en-1-yloxy)quinolin-2(1H)-one (7)

^{1}H-NMR (500 MHz, $CDCl_3$, 298K):

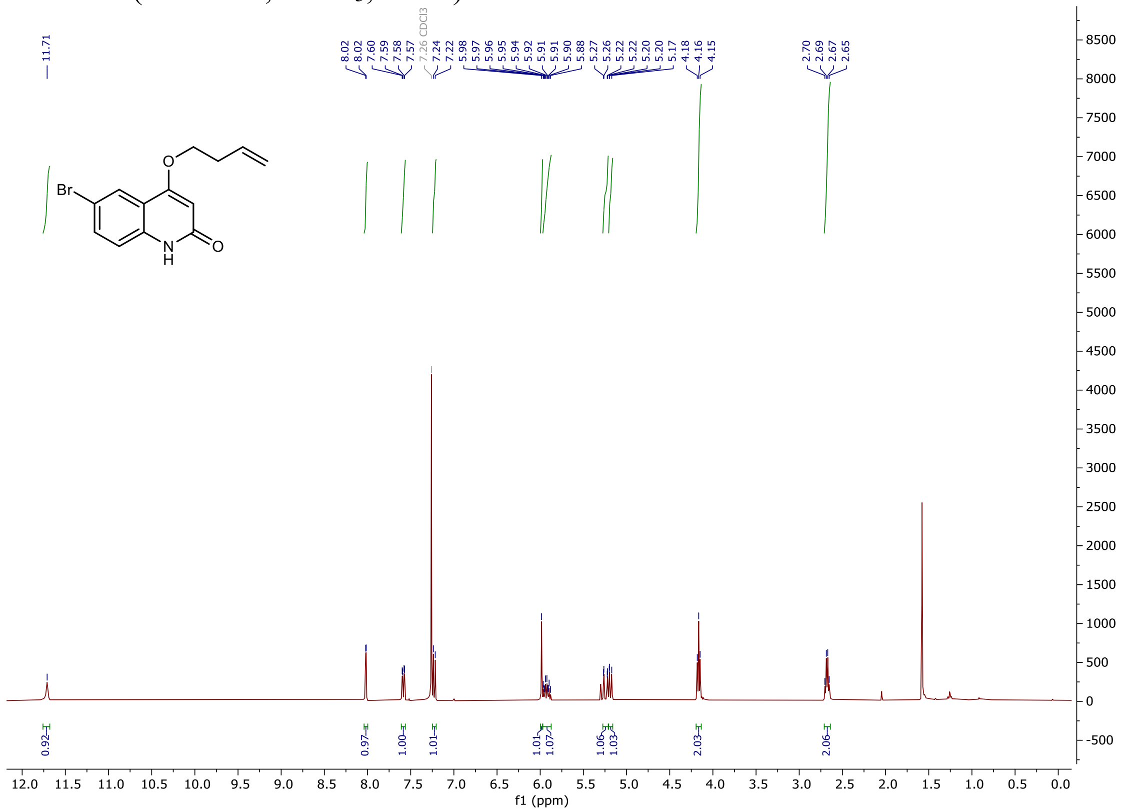

^{13}C-NMR (126 MHz, $CDCl_3$, 298K):

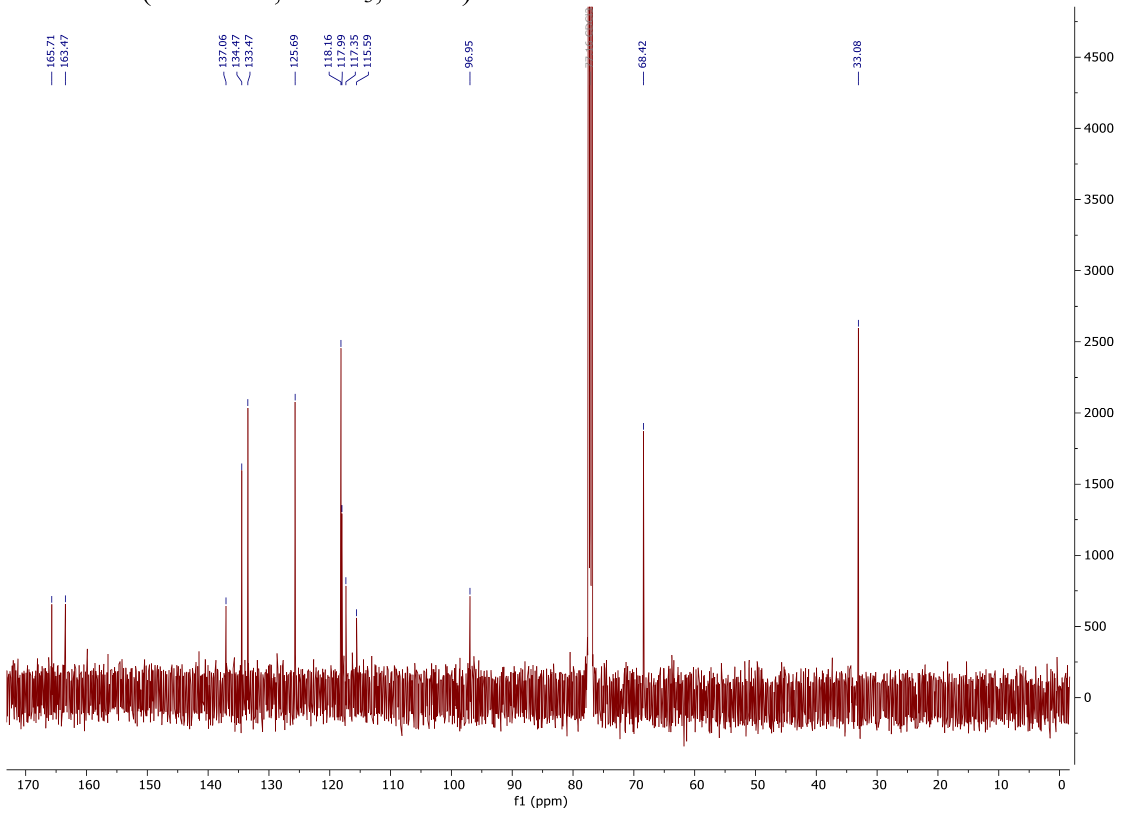

4-(but-3-en-1-yloxy)-8-fluoroquinolin-2(1H)-one (8)

^{1}H NMR (500 MHz, $CDCl_3$, 298K)

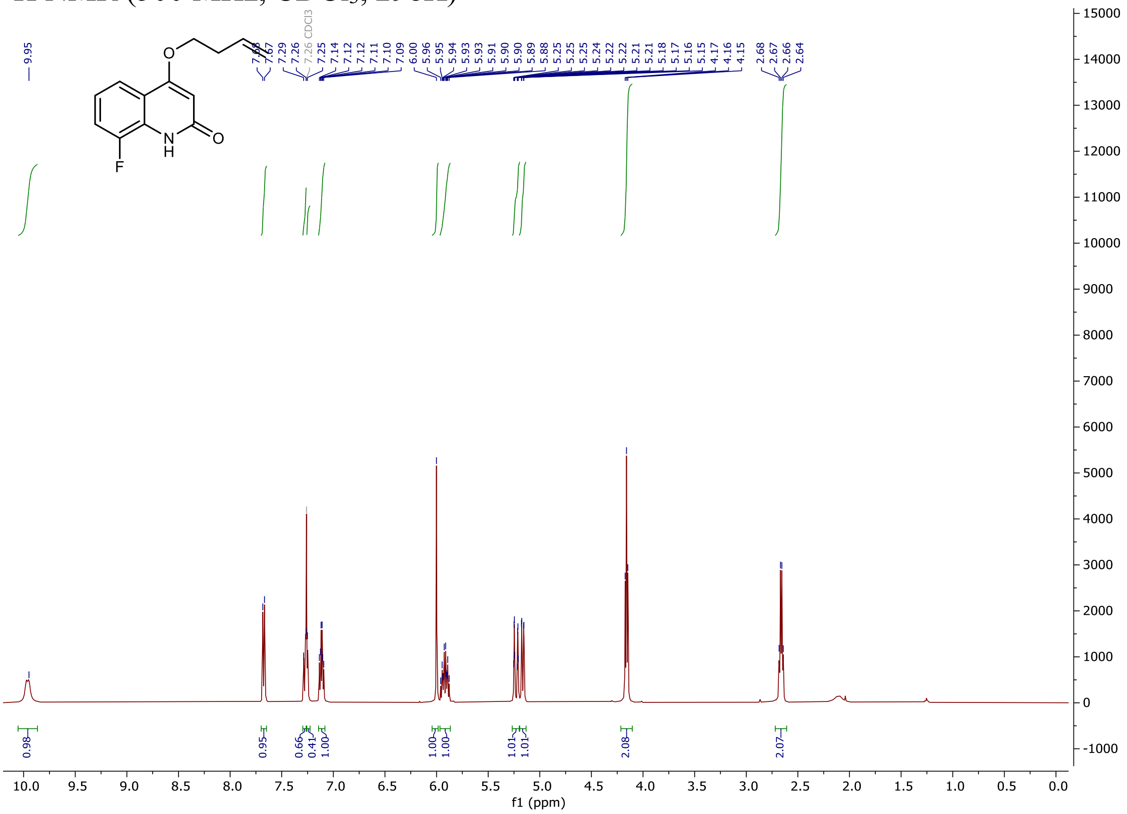

^{13}C NMR (126 MHz, $CDCl_3$, 298K)

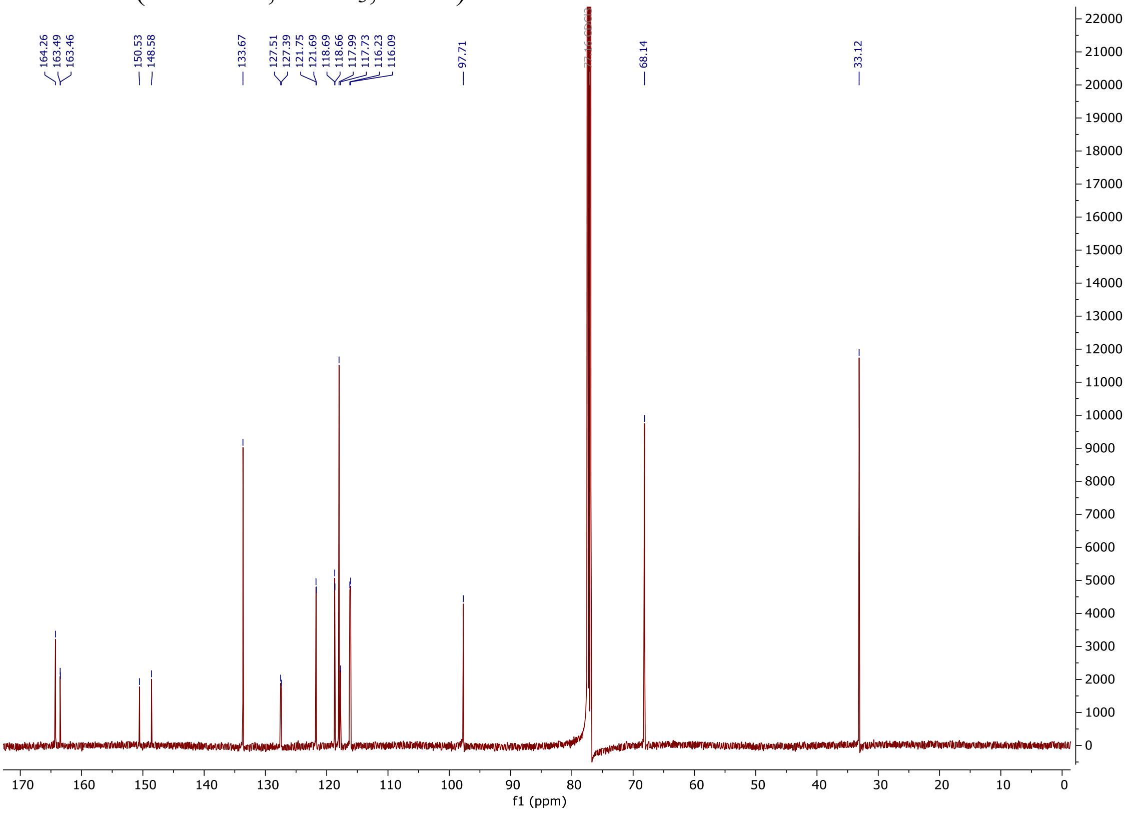

^{19}F NMR (471 MHz, $CDCl_3$)

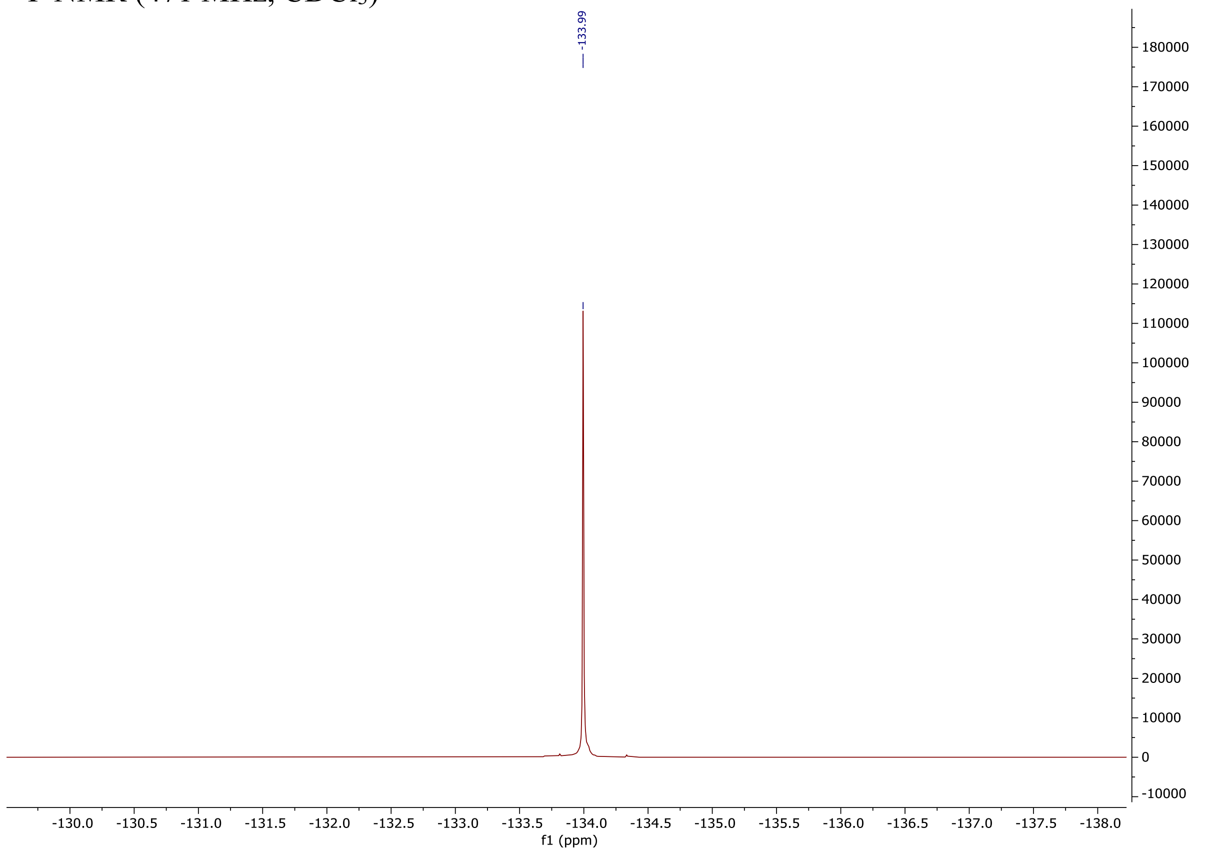

4-(but-3-en-1-yloxy)-7-methoxyquinolin-2(1H)-one (9)

^{1}H NMR (400 MHz, $CDCl_3$, 298K)

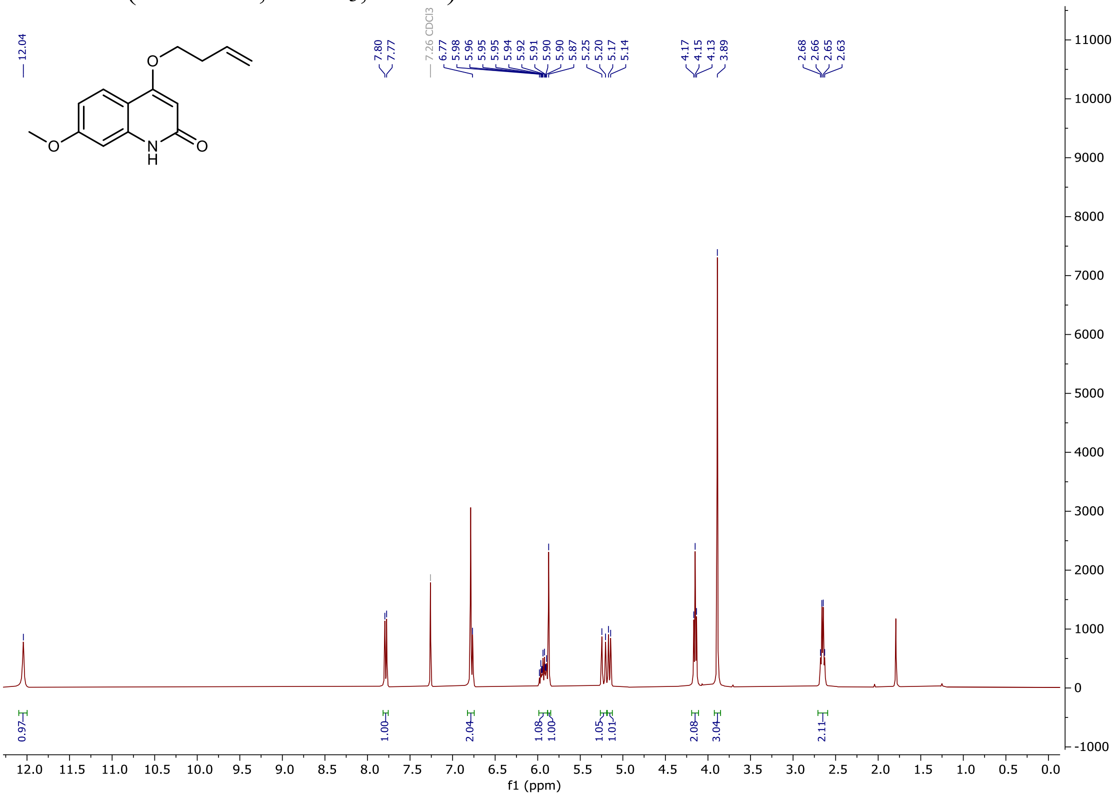

^{13}C NMR (101 MHz, $CDCl_3$, 298K)

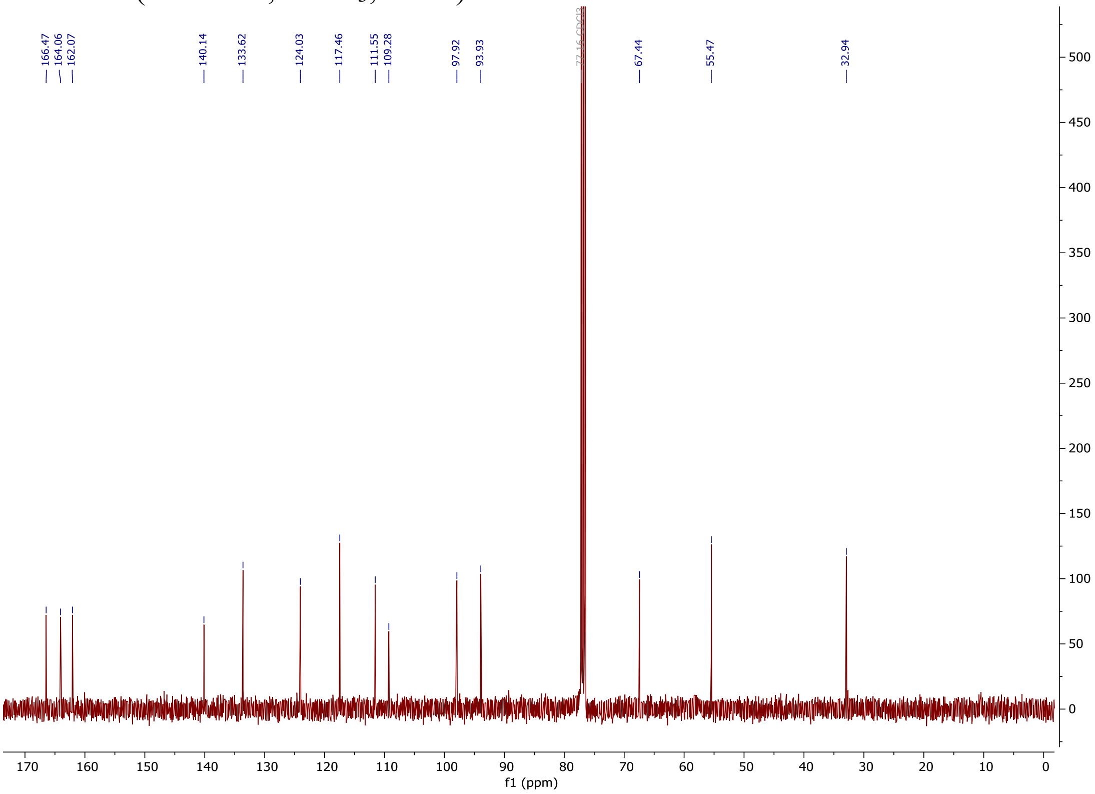

9-methyl-3,3a,4,4a-tetrahydro-2H-furo[2',3':2,3]cyclobuta[1,2-c]quinolin-5(6H)-one (4a, straight product)

^{1}H NMR (400 MHz, $CDCl_3$, 298K)

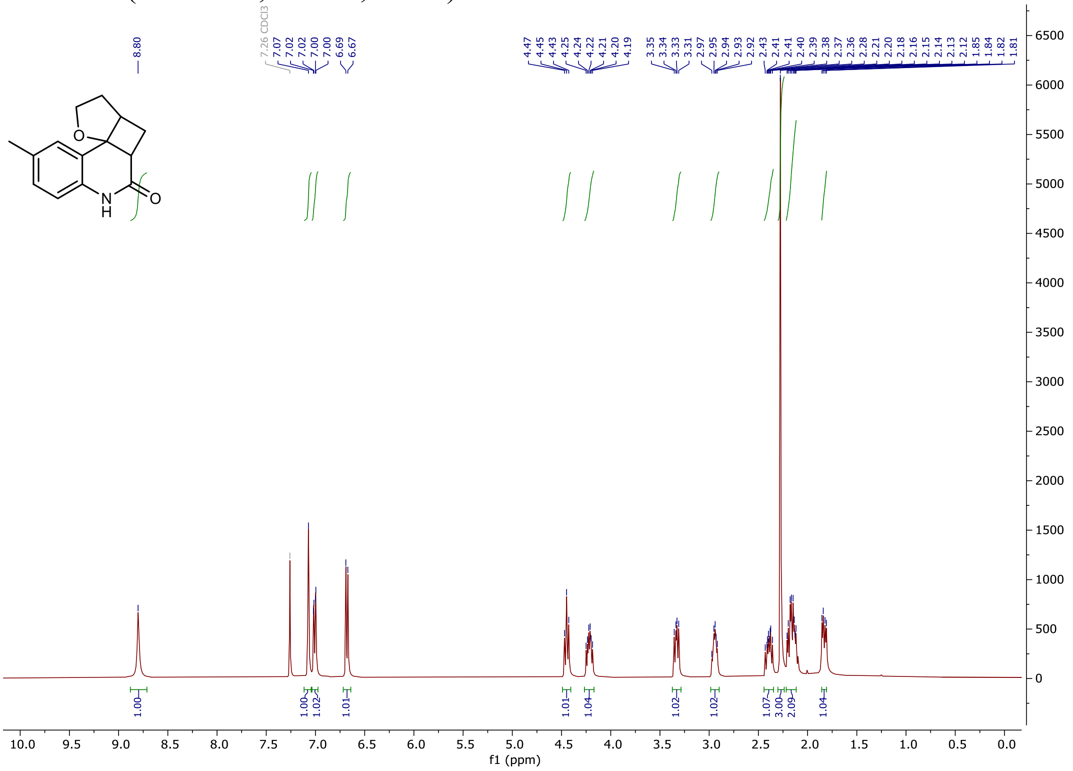

^{13}C NMR (101 MHz, $CDCl_3$, 298K)

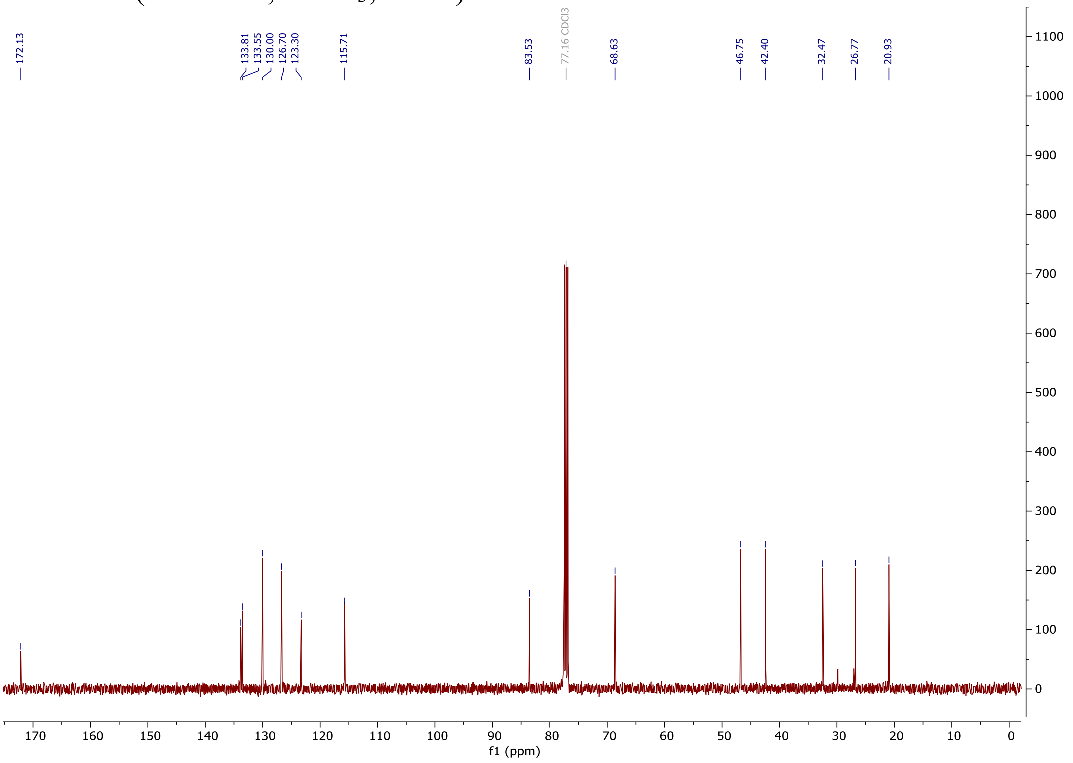

9-methyl-2,3,4,4a-tetrahydro-4,10b-methanopyrano[3,2-c]quinolin-5(6H)-one (4, crossed product)

^{1}H NMR (400 MHz, $CDCl_3$, 298K)

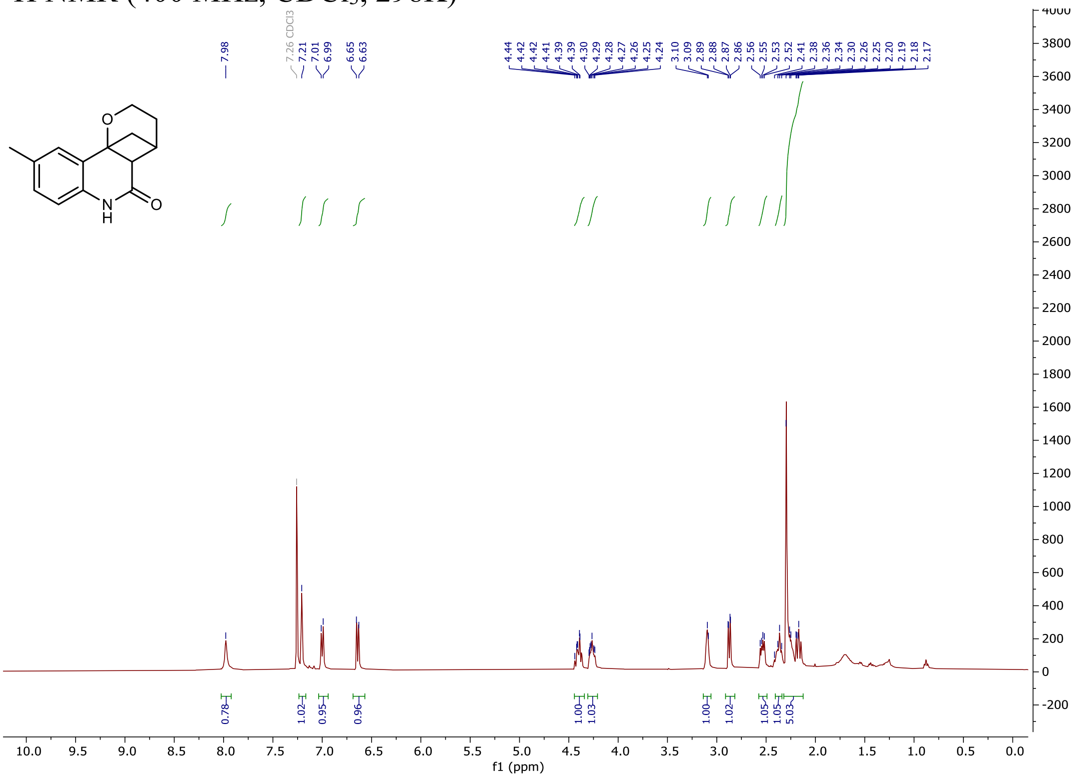

^{13}C NMR (126 MHz, $CDCl_3$, 298K)

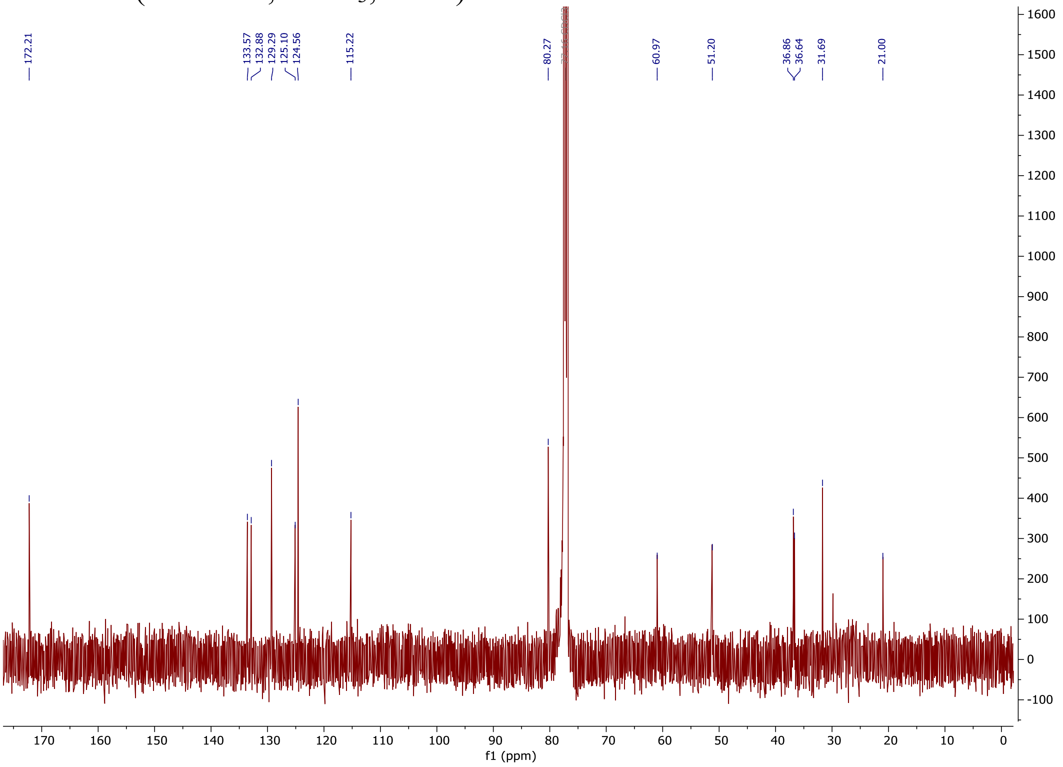

9-fluoro-3,3a,4,4a-tetrahydro-2H-furo[2',3':2,3]cyclobuta[1,2-c]quinolin-5(6H)-one (5a, straight product)

^{1}H NMR (400 MHz, CDCl$_3$, 298K)

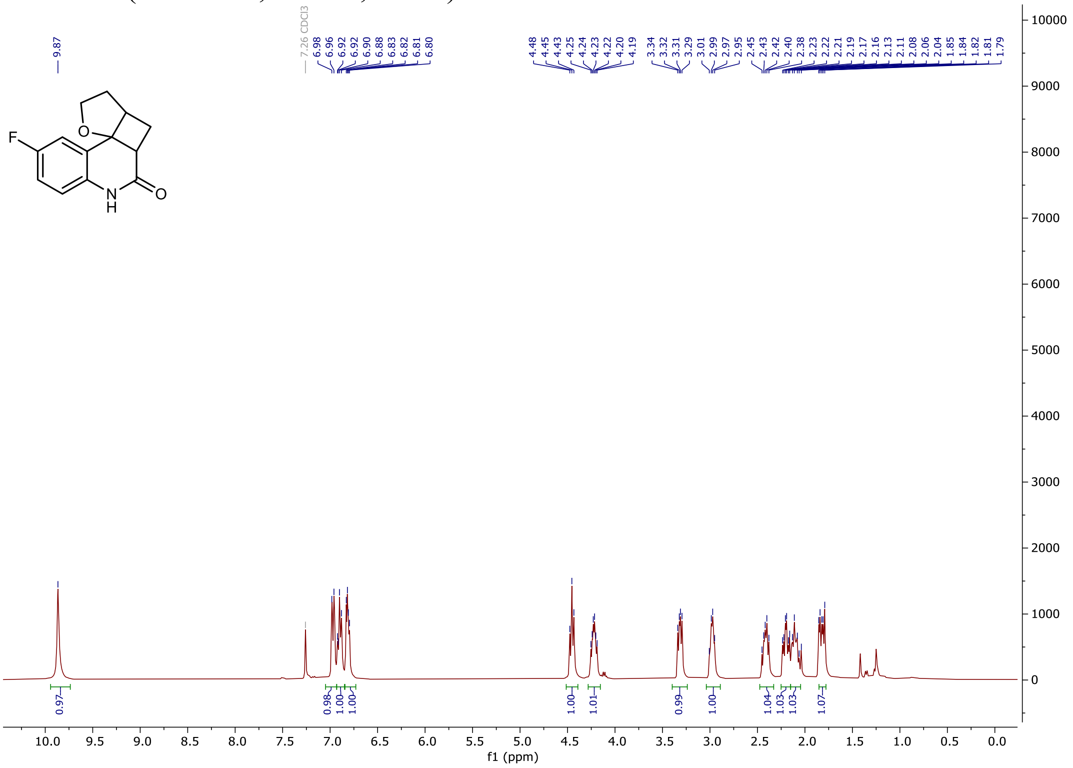

^{13}C NMR (101 MHz, CDCl$_3$, 298K)

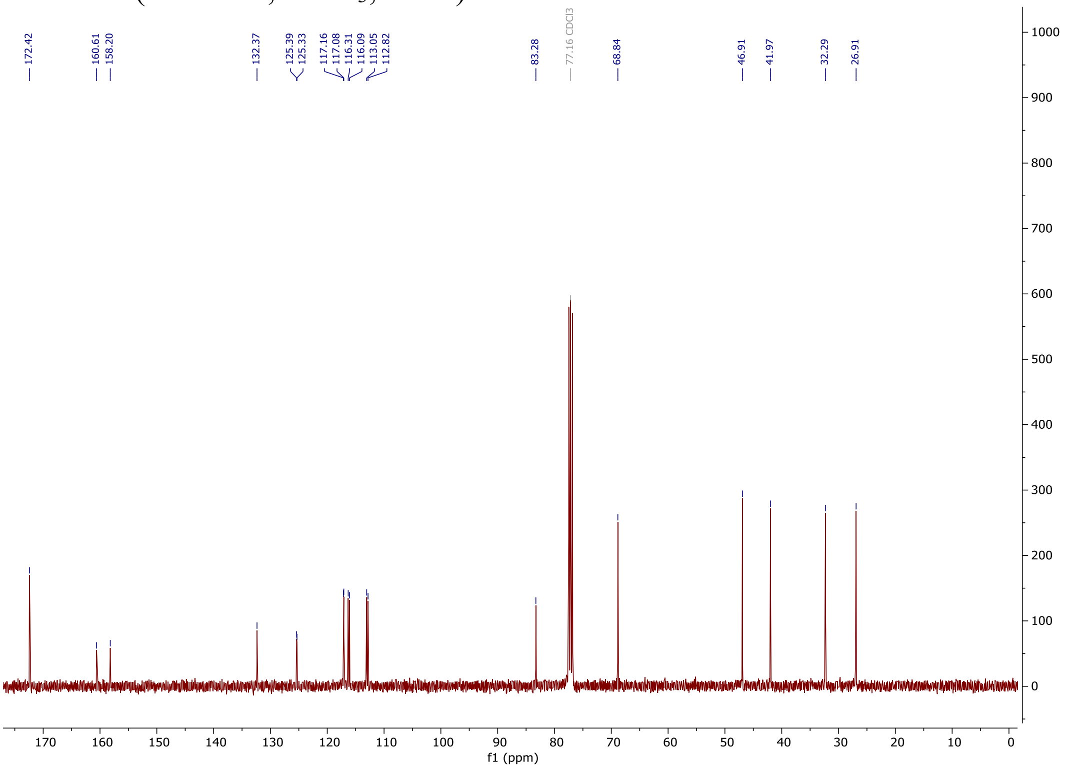

^{19}F NMR (376 MHz, $CDCl_3$, 298K)

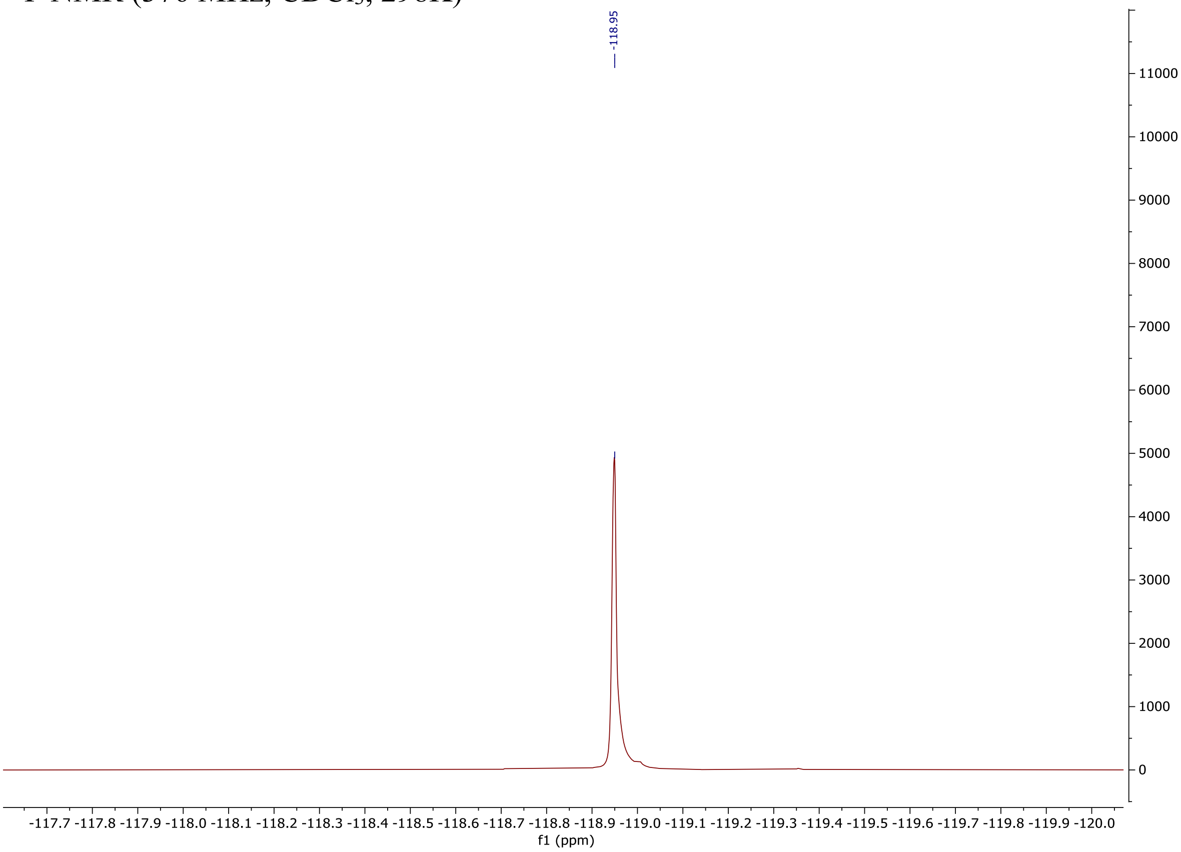

9-fluoro-2,3,4,4a-tetrahydro-4,10b-methanopyrano[3,2-c]quinolin-5(6H)-one (5, crossed product)

^{1}H NMR (500 MHz, $CDCl_3$, 298K)

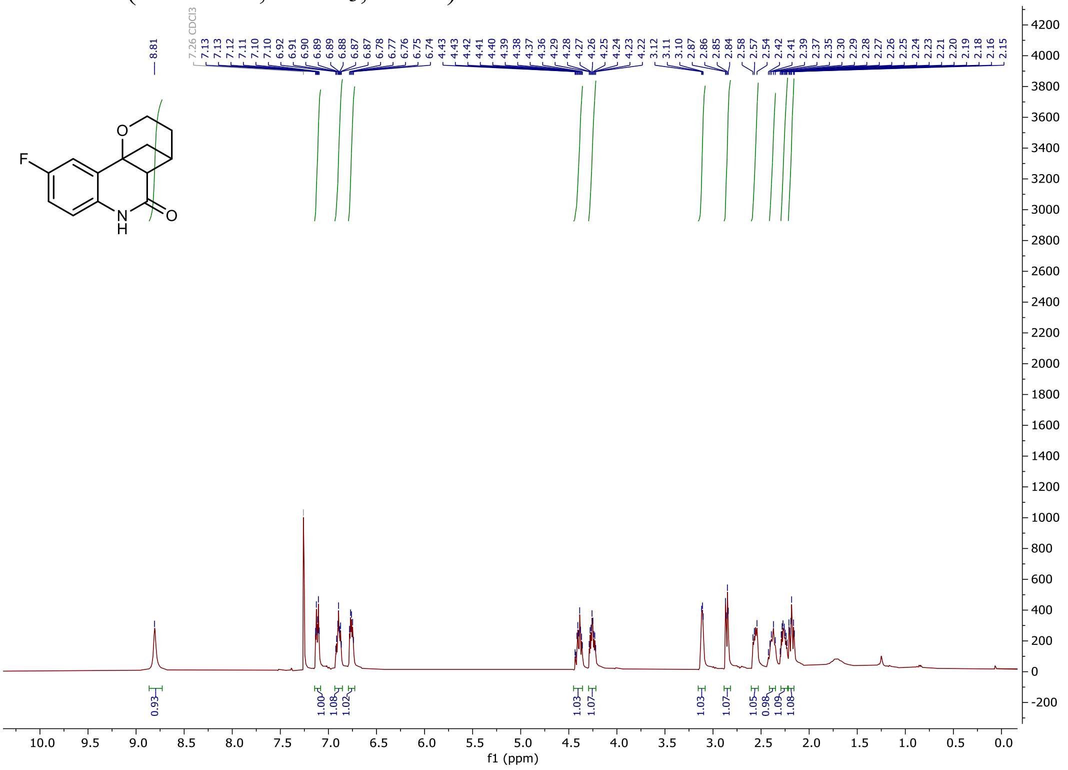

^{13}C NMR (126 MHz, $CDCl_3$, 298K)

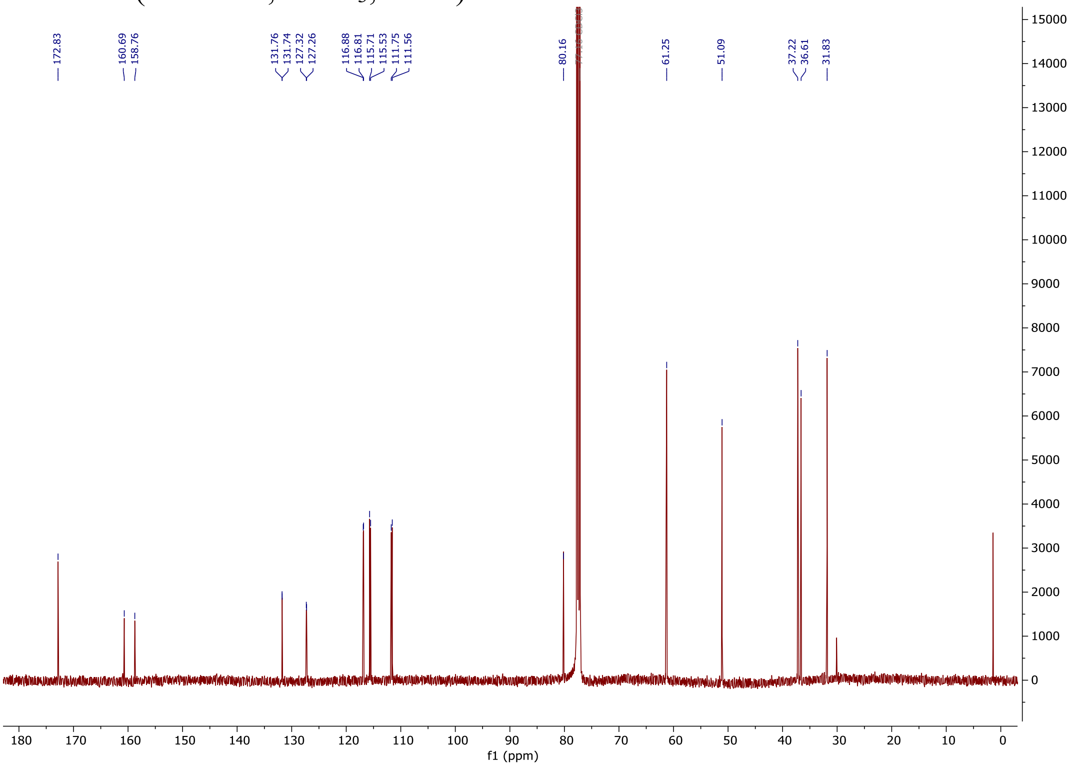

^{19}F NMR (471 MHz, $CDCl_3$, 298K)

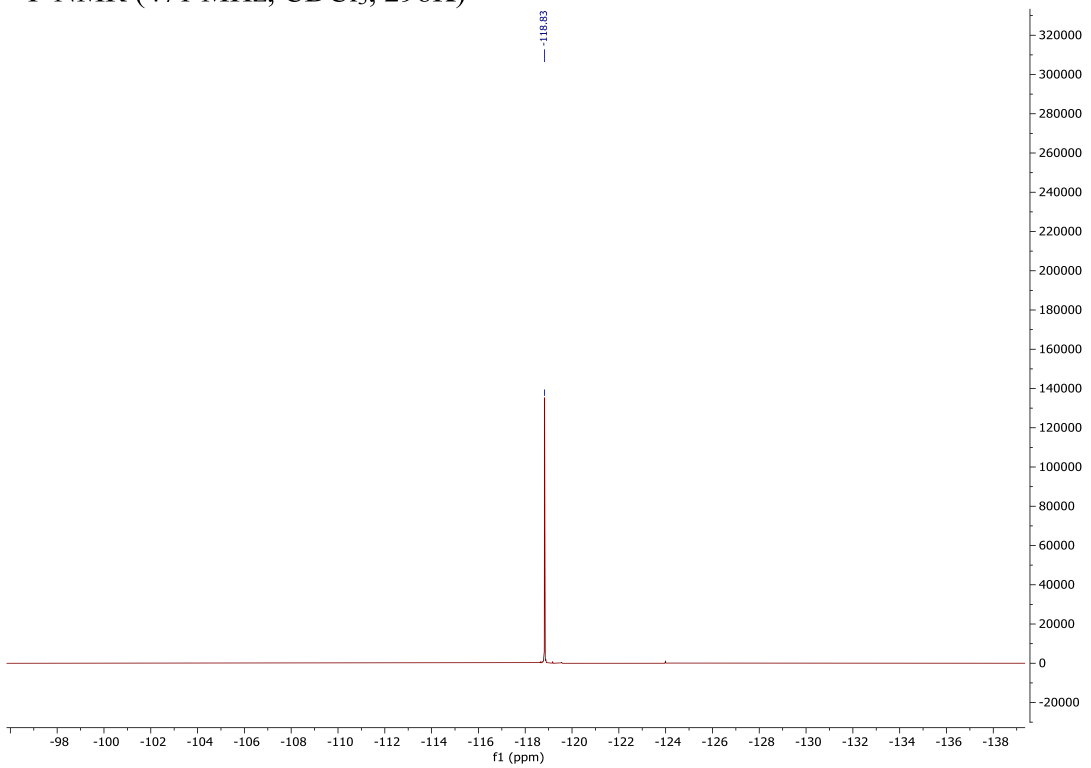

9-chloro-3,3a,4,4a-tetrahydro-2H-furo[2',3':2,3]cyclobuta[1,2-c]quinolin-5(6H)-one (6a, straight product)

^{1}H NMR (500 MHz, $CDCl_3$, 298K)

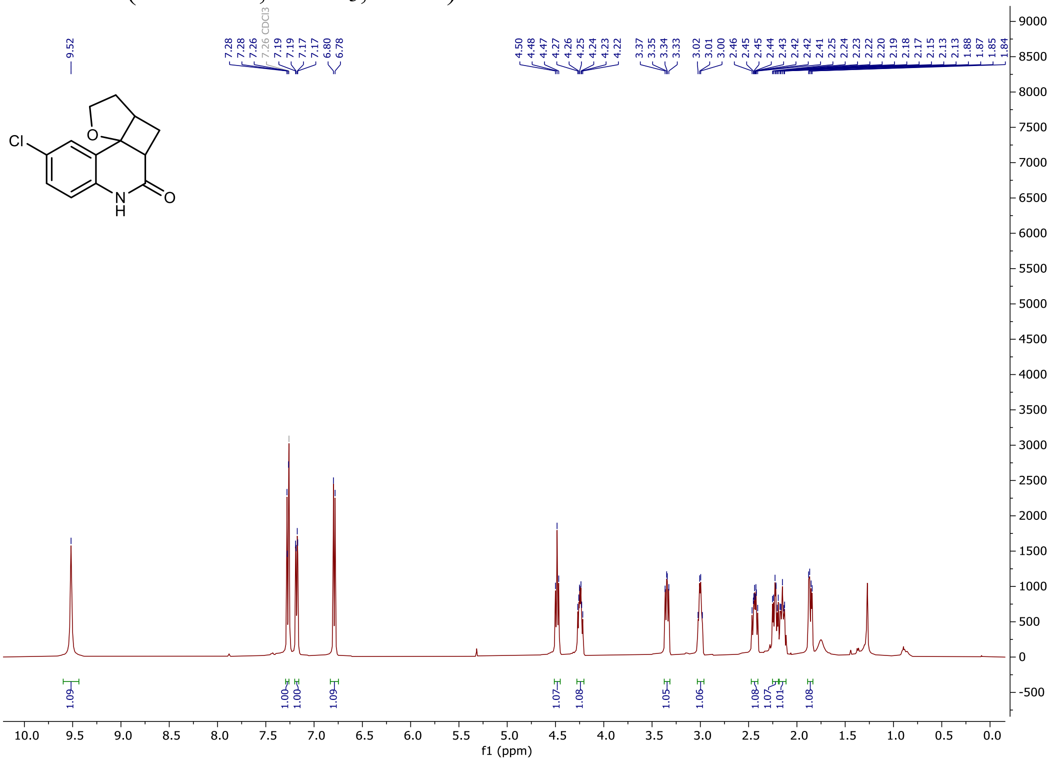

^{13}C NMR (101 MHz, $CDCl_3$, 298K)

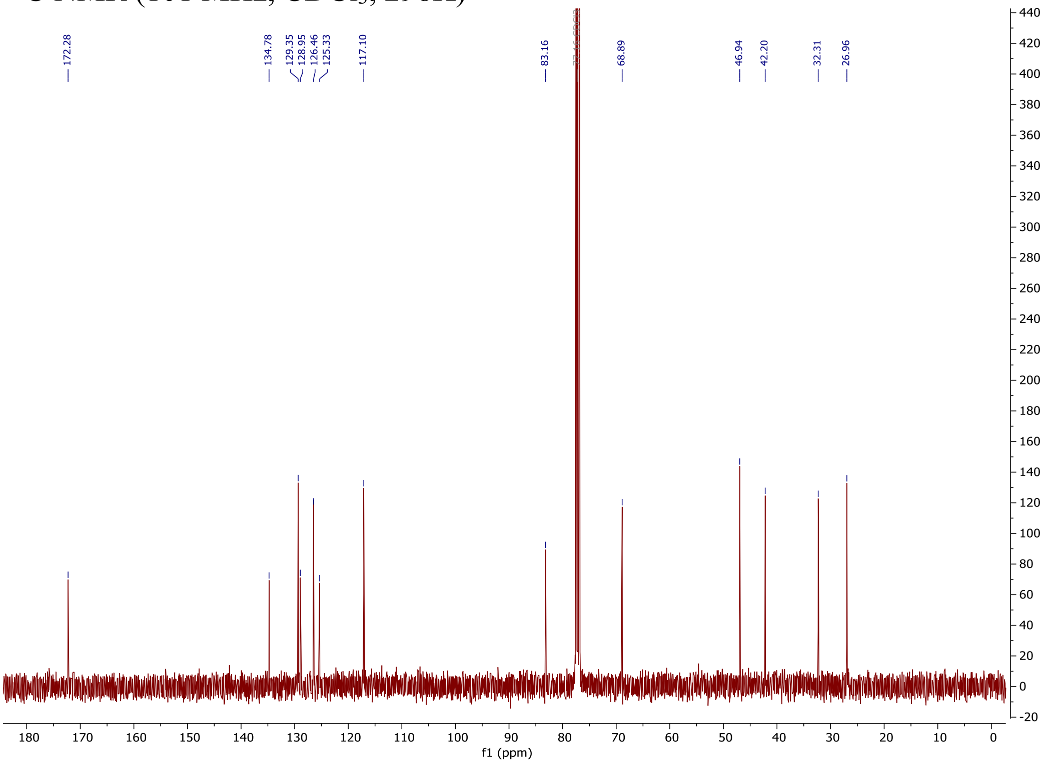

9-chloro-2,3,4,4a-tetrahydro-4,10b-methanopyrano[3,2-c]quinolin-5(6H)-one (6, crossed product)

^{1}H NMR (500 MHz, $CDCl_3$, 298K)

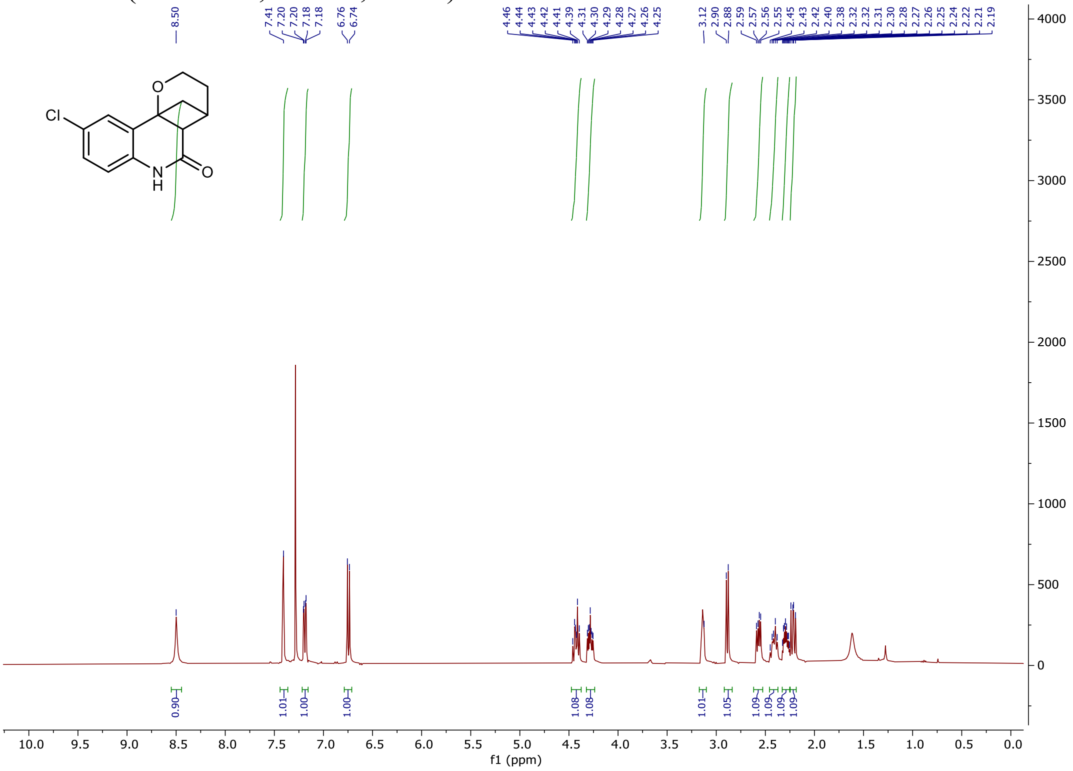

^{13}C NMR (101 MHz, $CDCl_3$, 298K)

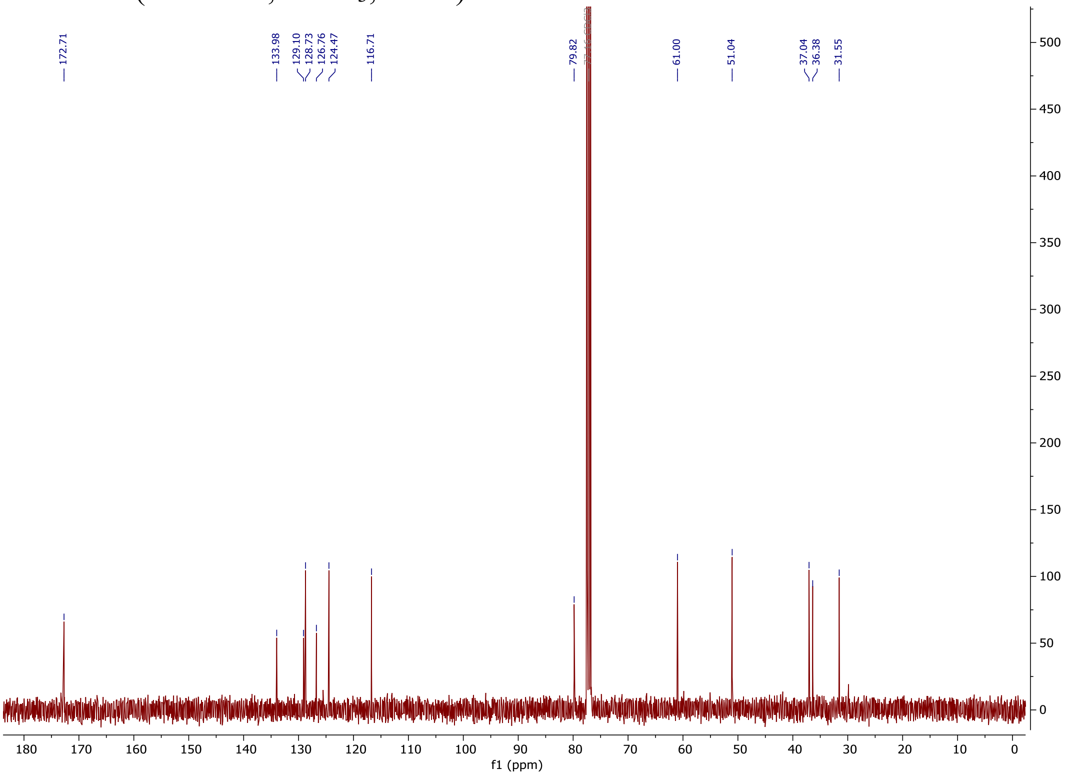

9-bromo-3,3a,4,4a-tetrahydro-2H-furo[2',3':2,3]cyclobuta[1,2-c]quinolin-5(6H)-one (7a, straight product)

^{1}H NMR (500 MHz, $CDCl_3$, 298K)

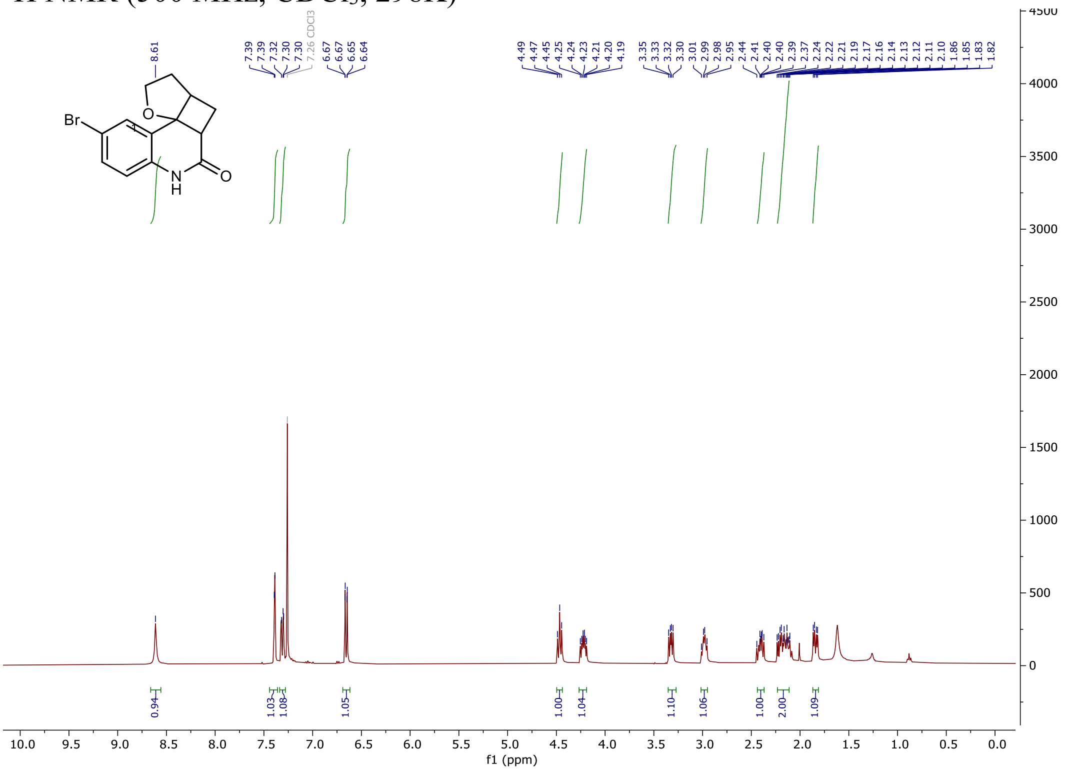

^{13}C NMR (126 MHz, $CDCl_3$, 298K)

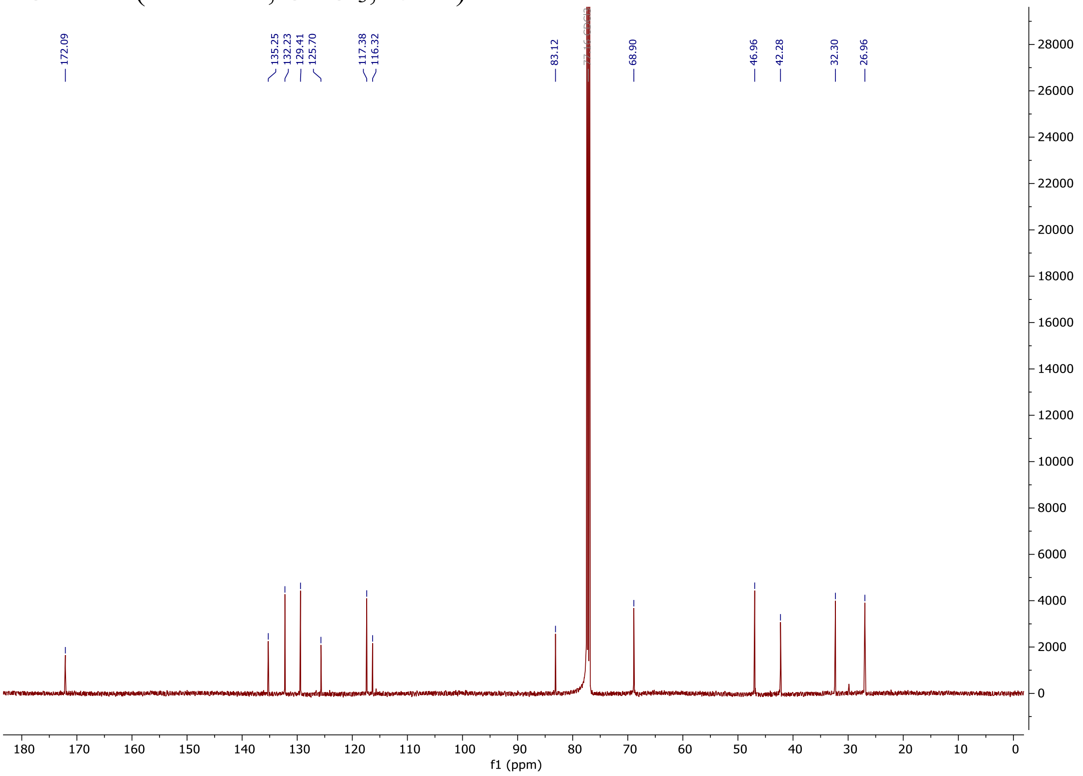

9-bromo-2,3,4,4a-tetrahydro-4,10b-methanopyrano[3,2-c]quinolin-5(6H)-one (7, crossed product)

^{1}H NMR (500 MHz, $CDCl_3$, 298K)

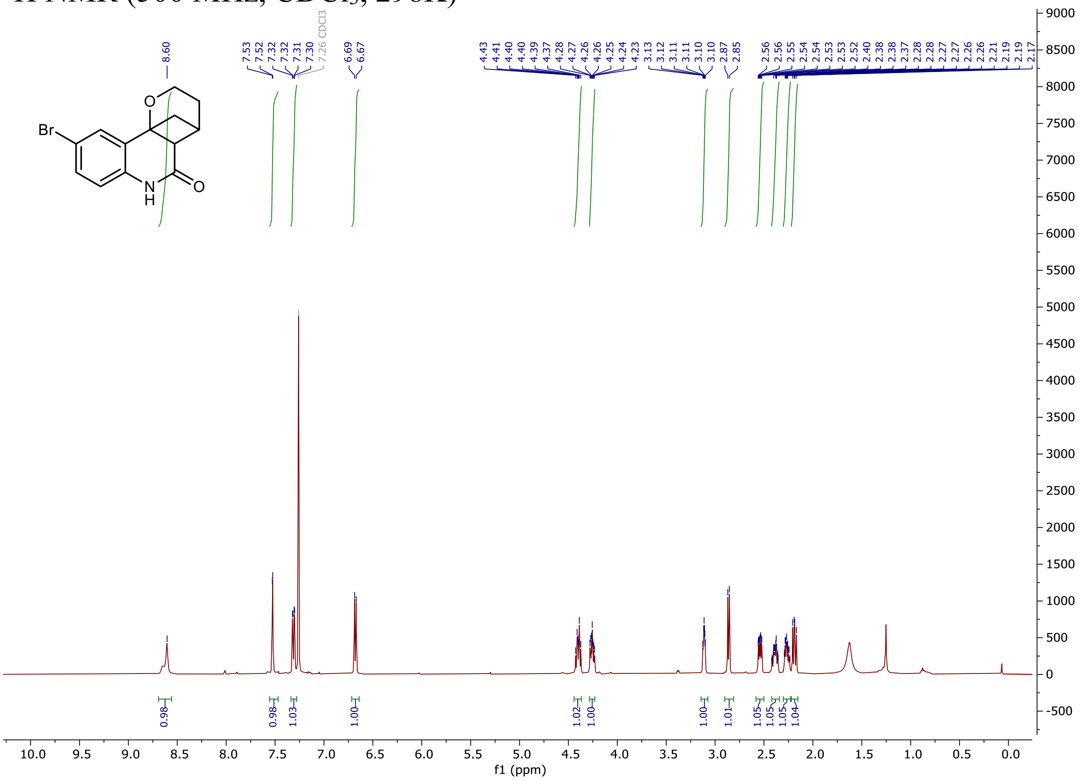

^{13}C NMR (126 MHz, $CDCl_3$, 298K)

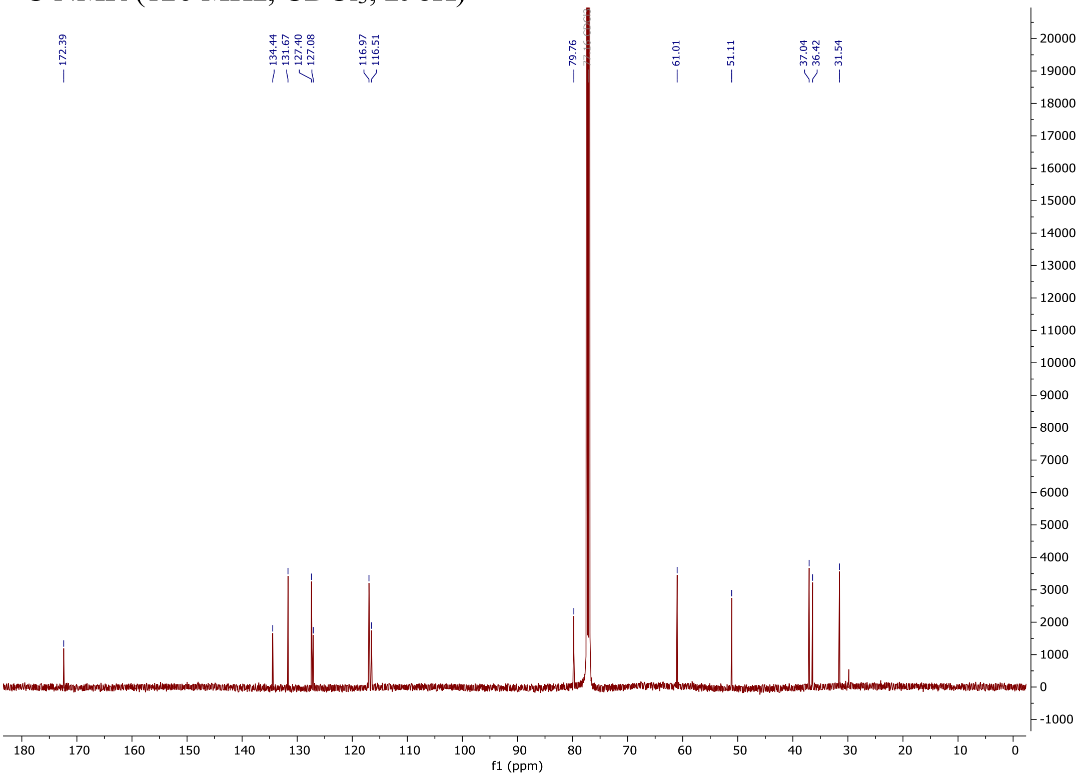

7-fluoro-3,3a,4,4a-tetrahydro-2H-furo[2',3':2,3]cyclobuta[1,2-c]quinolin-5(6H)-one (8a, straight)

^{1}H NMR (500 MHz, $CDCl_3$, 298K)

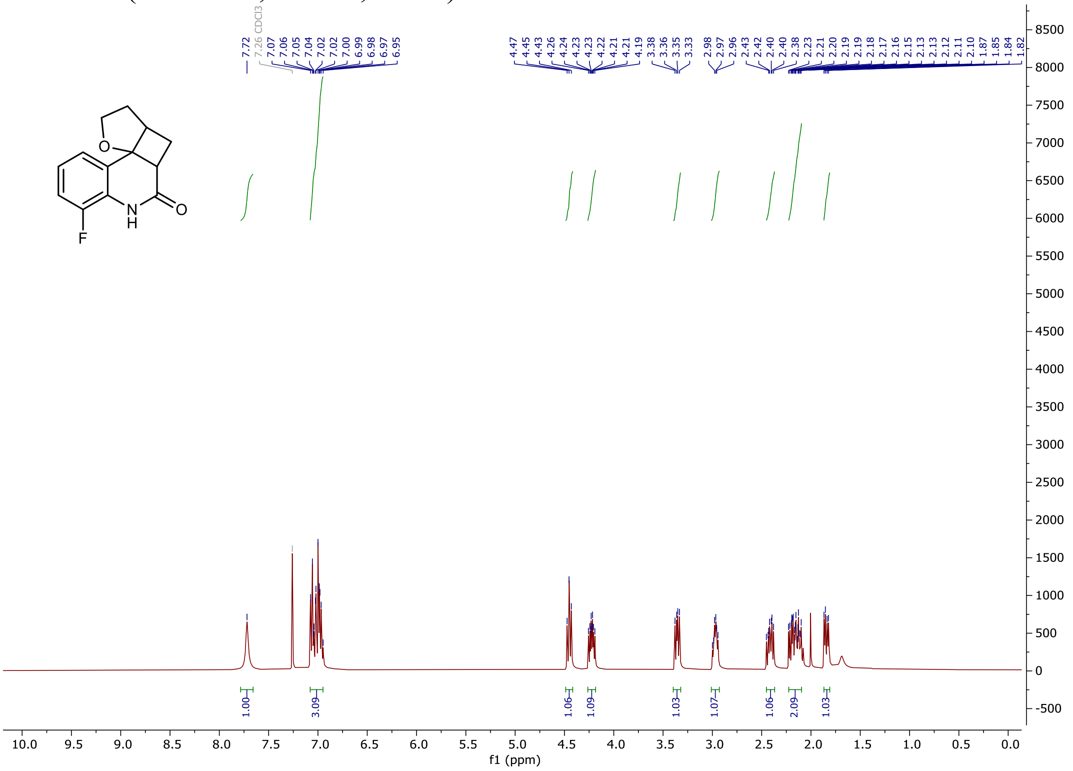

^{13}C NMR (126 MHz, $CDCl_3$, 298K)

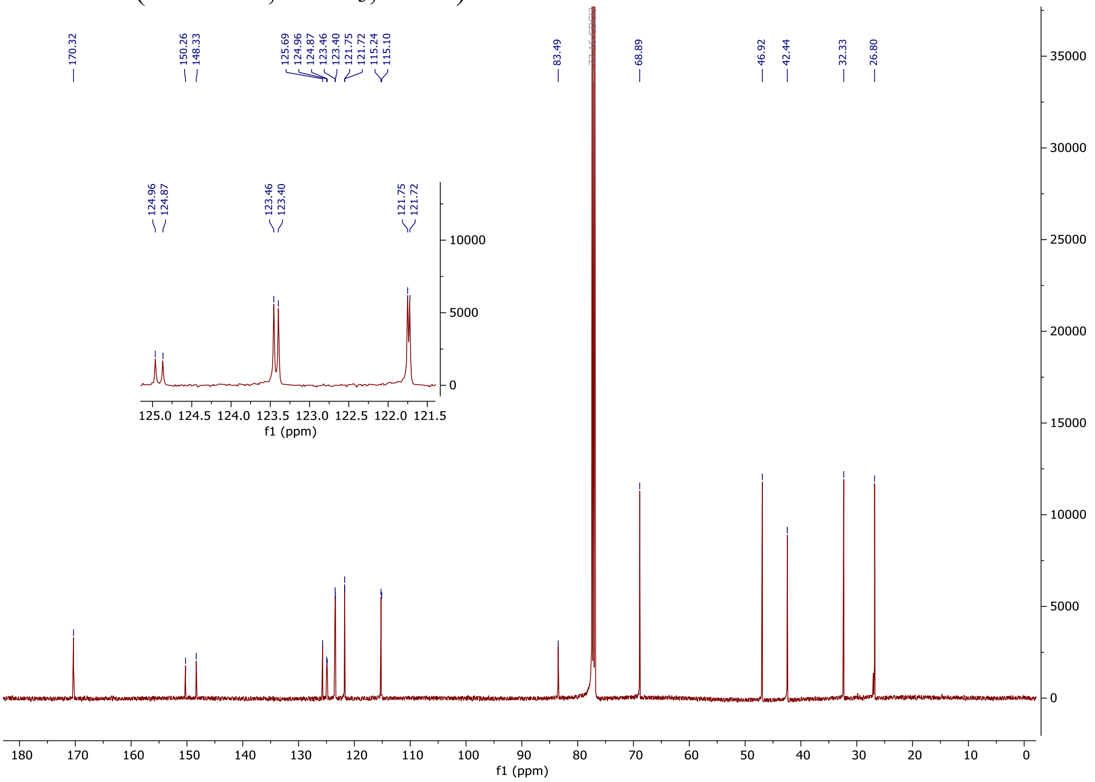

^{19}F NMR (471 MHz, $CDCl_3$, 298K)

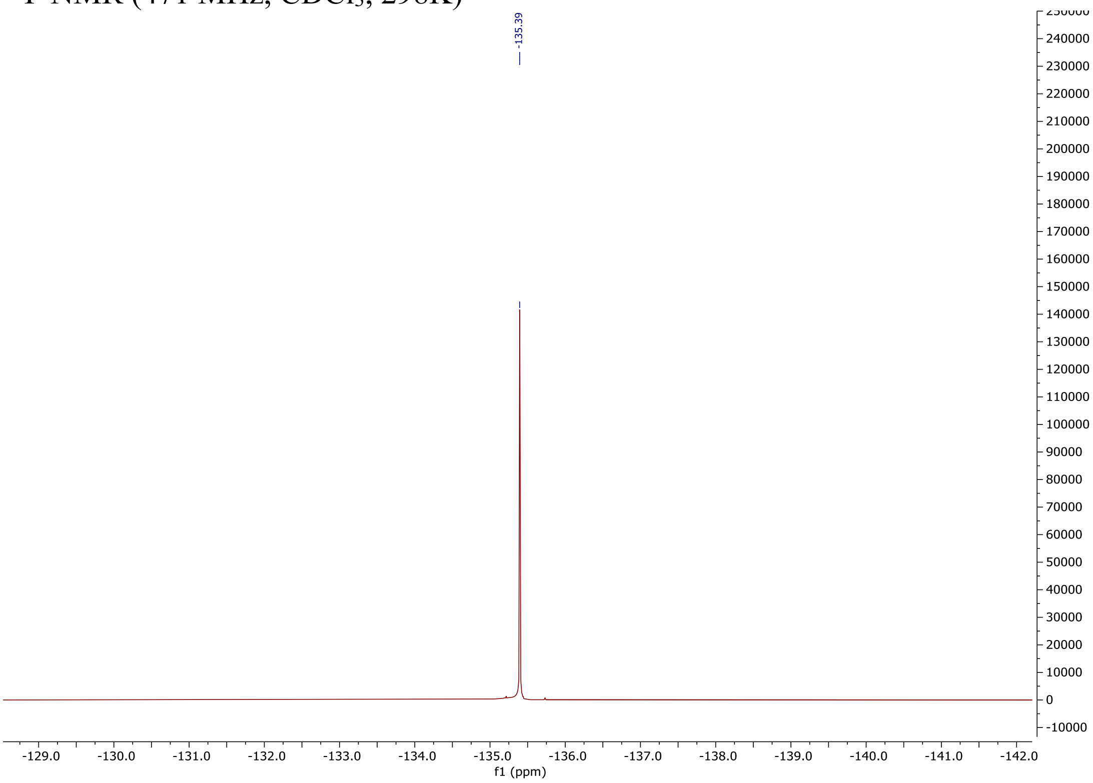

7-fluoro-2,3,4,4a-tetrahydro-4,10b-methanopyrano[3,2-c]quinolin-5(6H)-one (8, crossed product)

^{1}H NMR (400 MHz, $CDCl_3$, 298K)

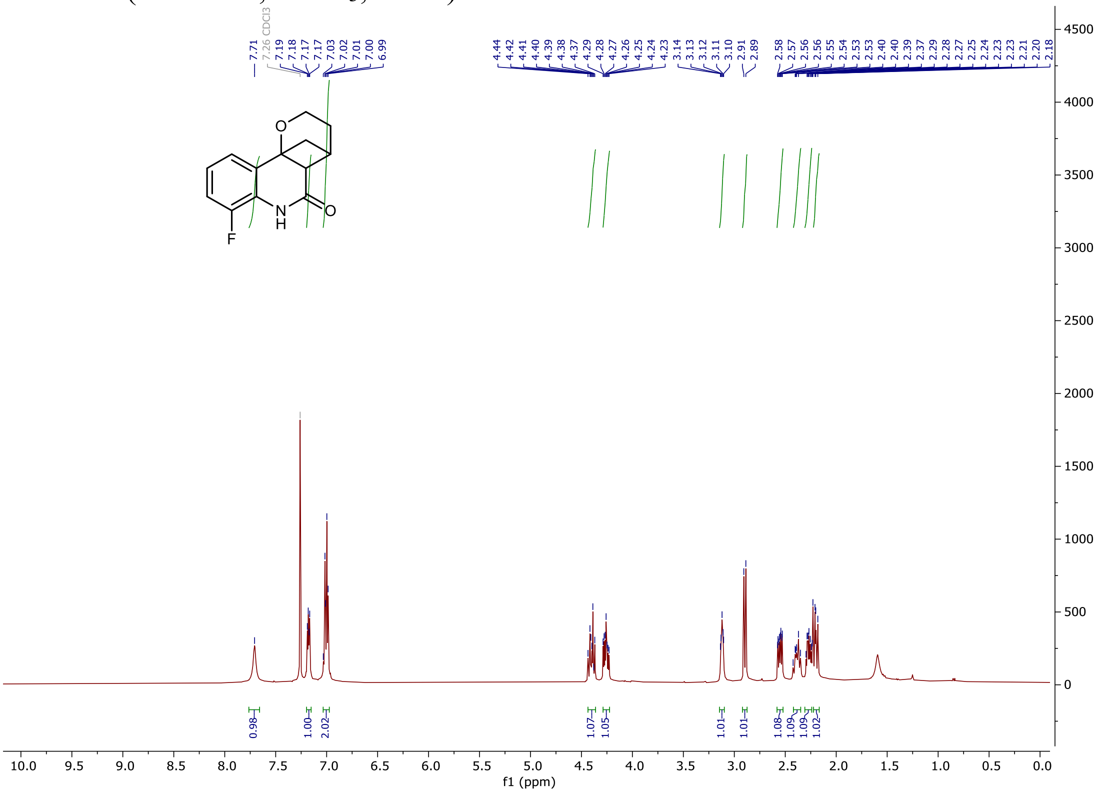

^{13}C NMR (126 MHz, $CDCl_3$, 298K)

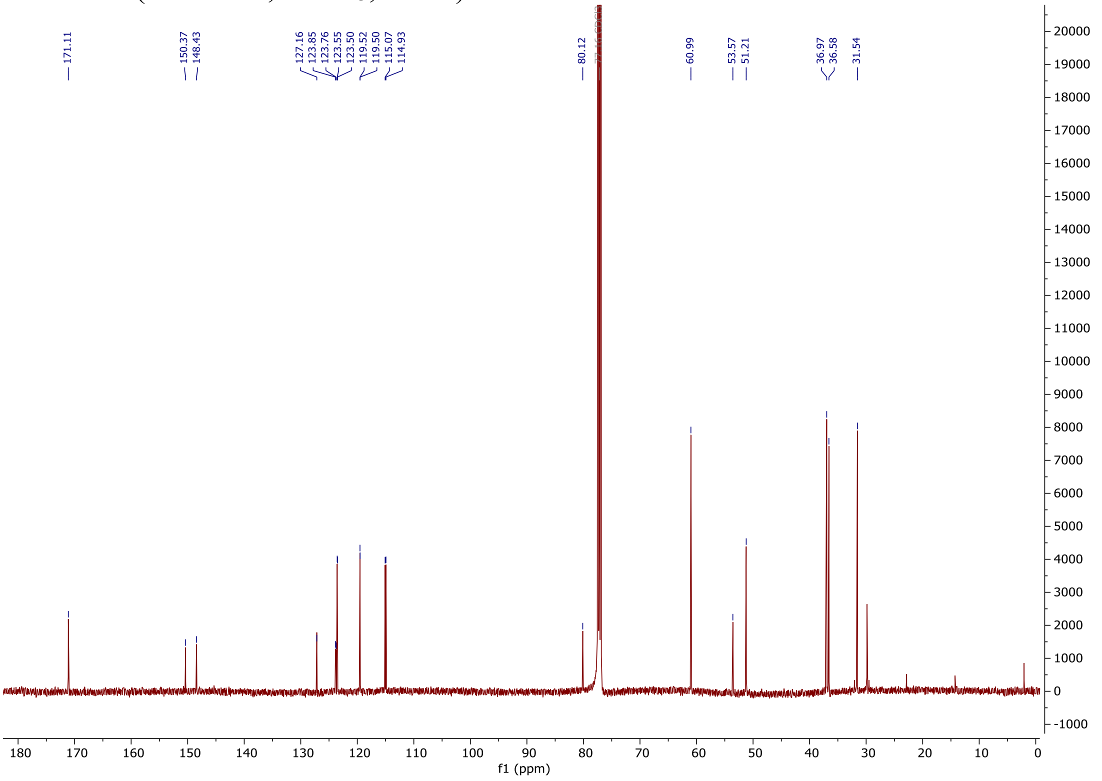

^{19}F NMR (376 MHz, $CDCl_3$, 298K)

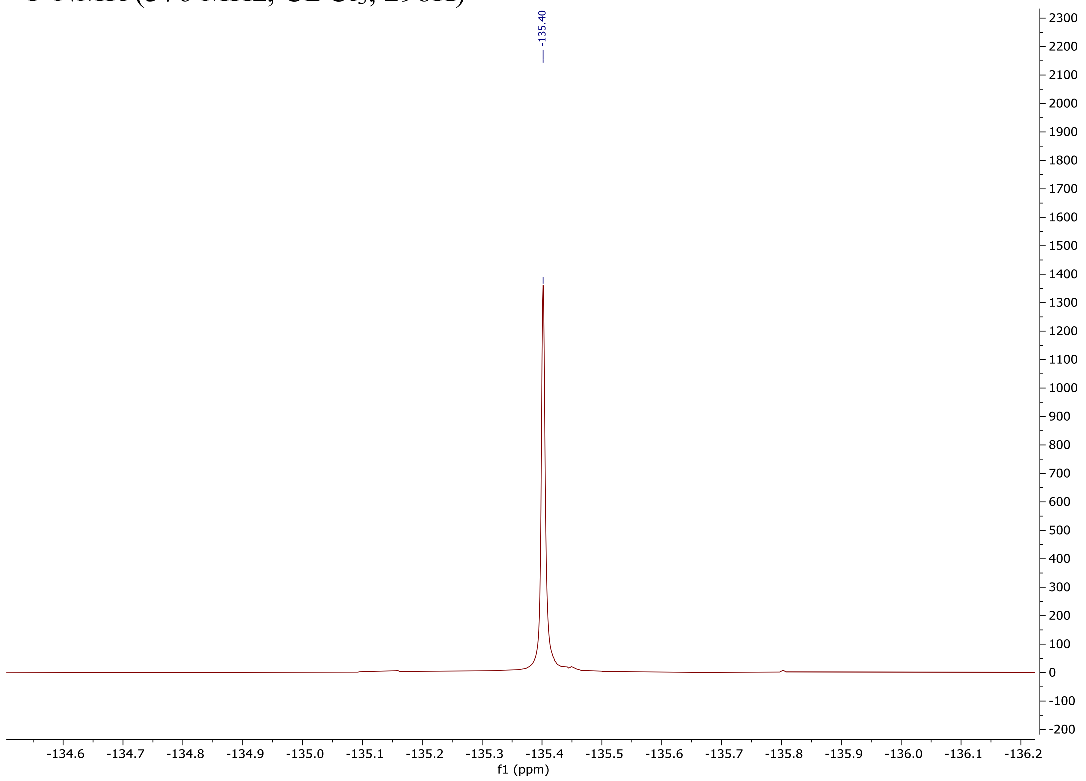

8-methoxy-3,3a,4,4a-tetrahydro-2H-furo[2',3':2,3]cyclobuta[1,2-c]quinolin-5(6H)-one (9a, straight product)

^{1}H NMR (500 MHz, $CDCl_3$, 298K)

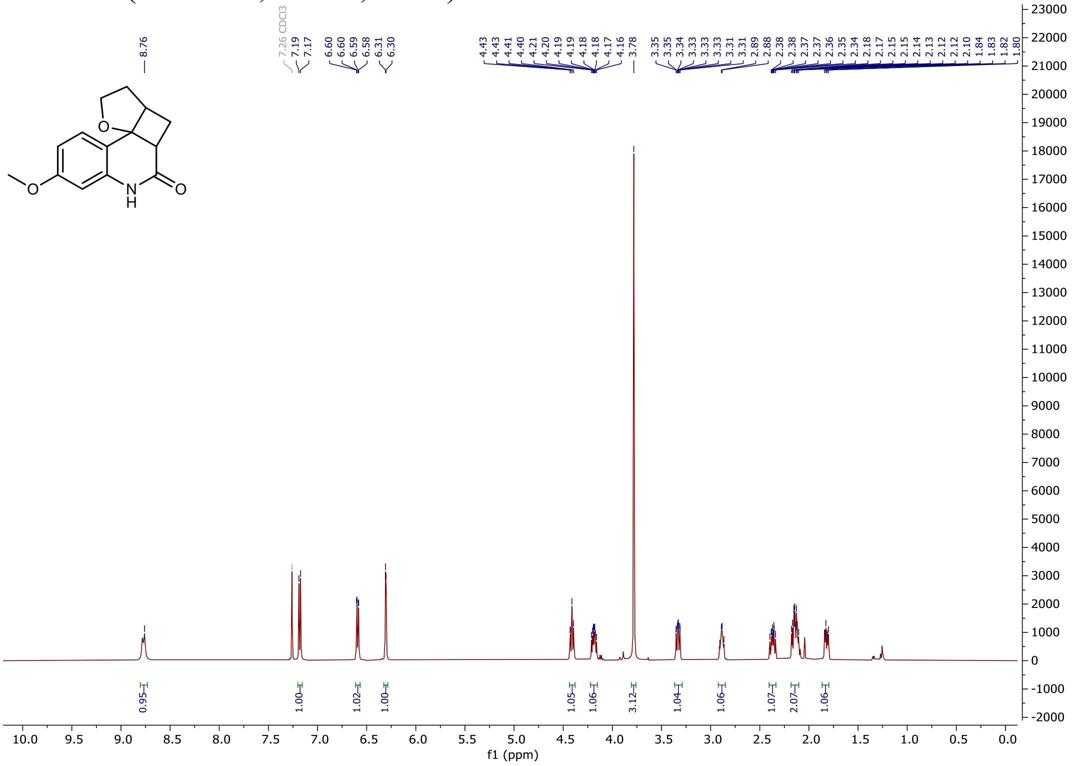

^{13}C NMR (126 MHz, $CDCl_3$, 298K)

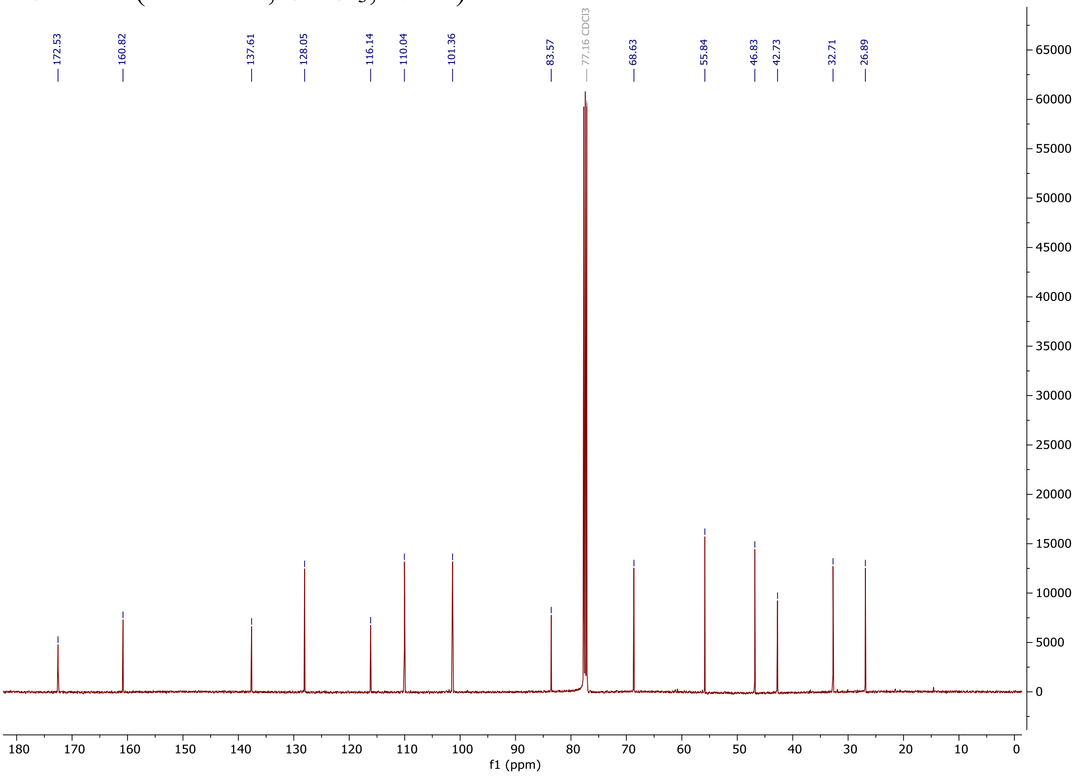

Substrate Scope Chromatograms

Substrate 1

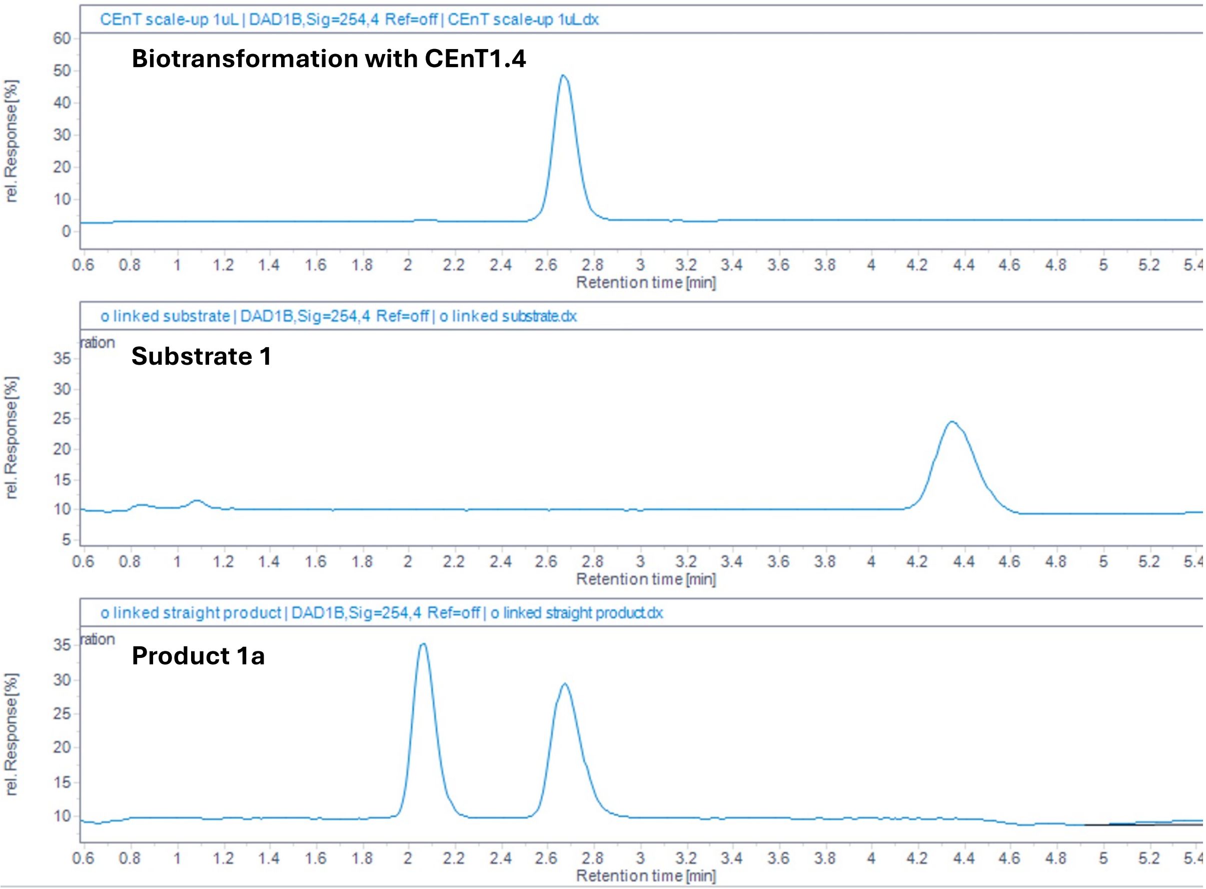

SFC (IG-3 SFC column, 3 mm x 50 mm, CO2/methanol=76:24). Substrate **1** elutes at 4.4 min while product enantiomer **(-)-1a** elutes at 2.0 min and product enantiomer **(+)-1a** elutes at 2.7 min.

Substrate 2

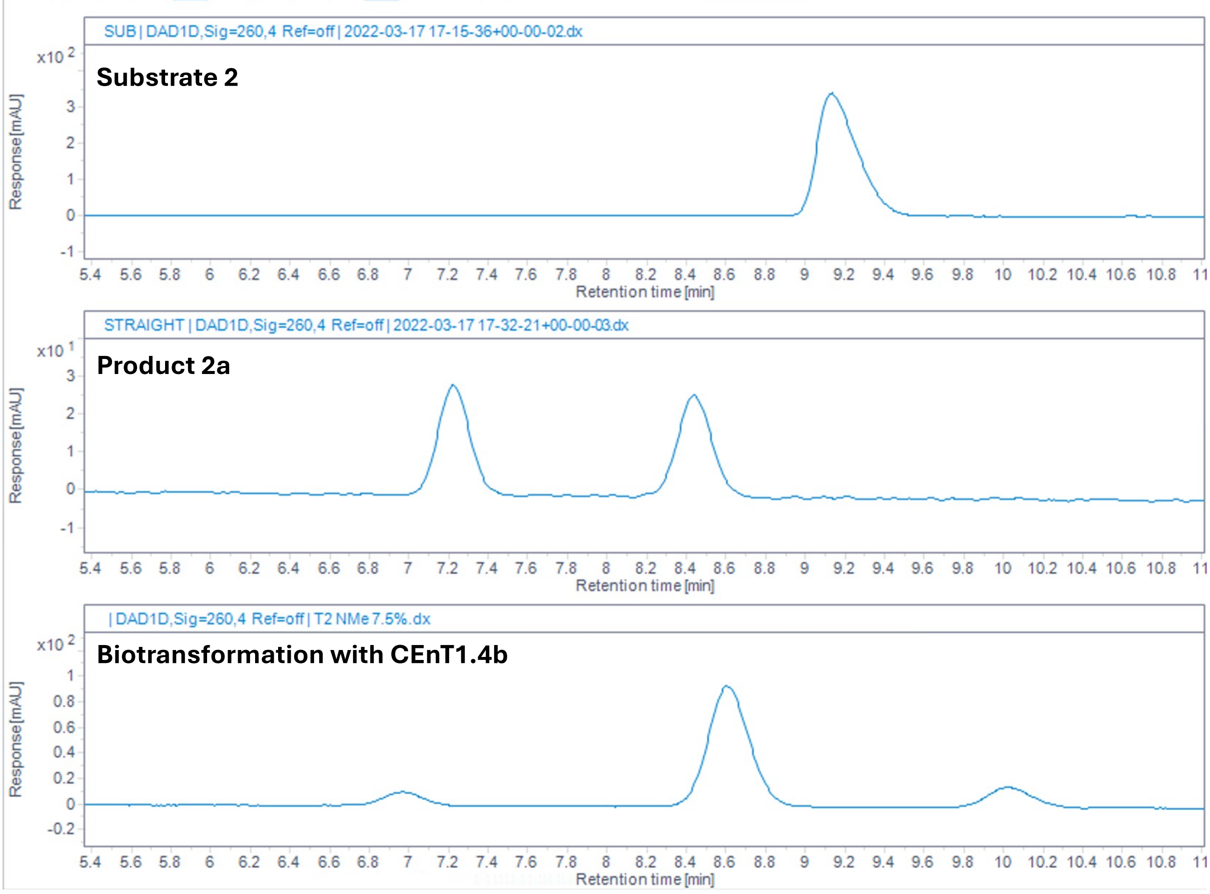

SFC (IG-3 SFC column, 3 mm x 50 mm, CO2/methanol=97:3 to 85:15 gradient over 15 minutes). Substrate **2** elutes at 10 min while the two enantiomers of product **2a** elute at 7.0 min and 8.6 min.

Substrate 3

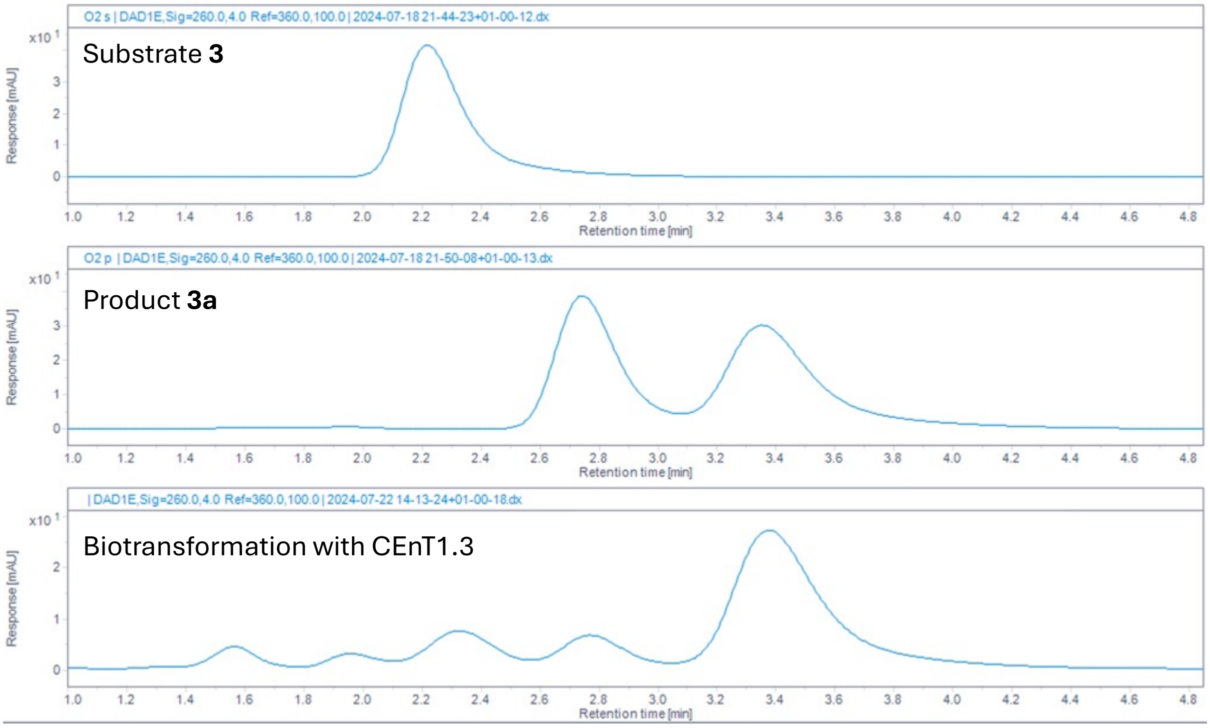

UPLC (IG-3 SFC column, 3 mm x 50 mm, Hexane/IPA=94:6). Substrate **3** elutes at 2.2 min while the two enantiomers of product **3a** elute at 2.8 min and 3.4 min. (Note: the peaks at 1.6 and 2.0 are enantiomers of product **3b**).

Substrate 4

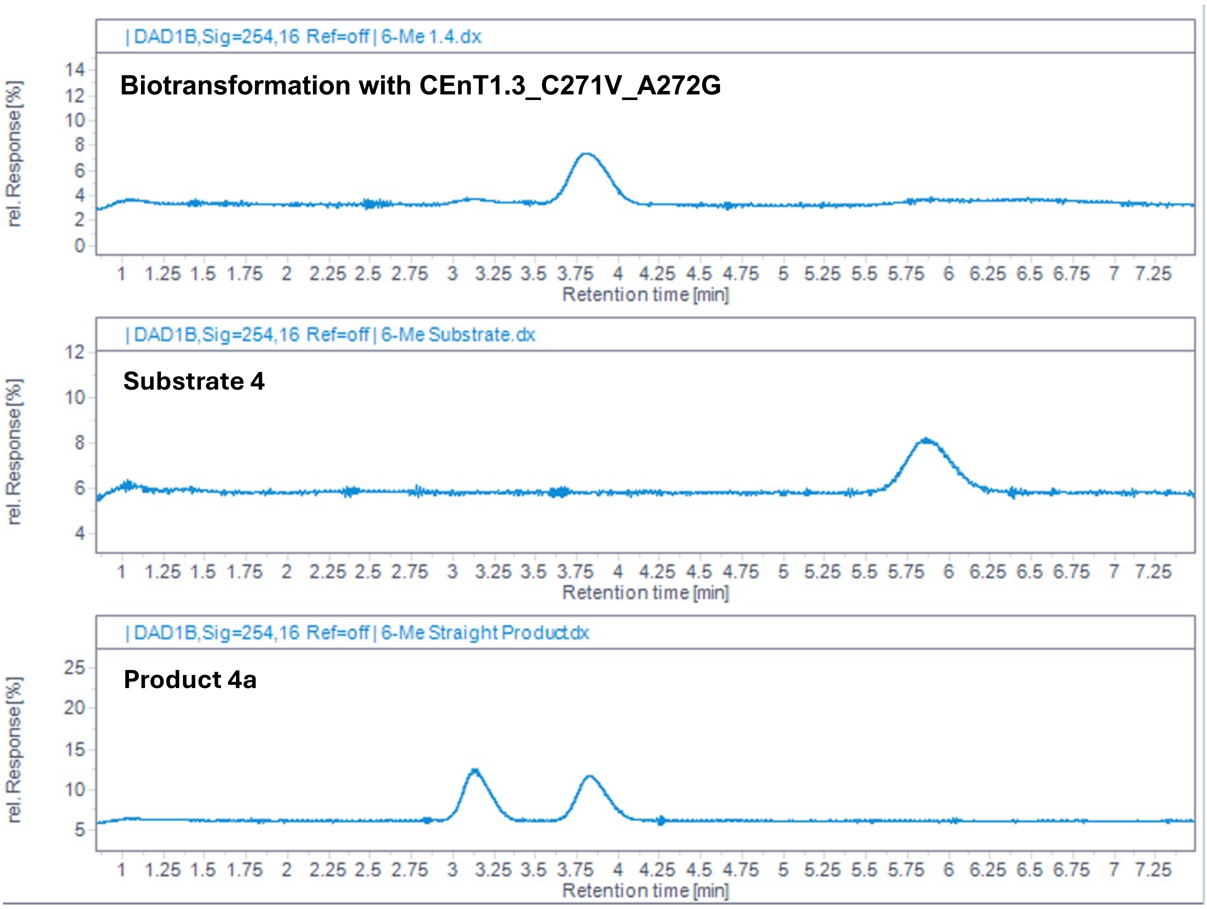

SFC (IG-3 SFC column, 3 mm x 50 mm, CO2/methanol=85:15). Substrate **4** elutes at 5.8 min while the two enantiomers of product **4a** elute at 3.1 min and 3.8 min.

Substrate 5

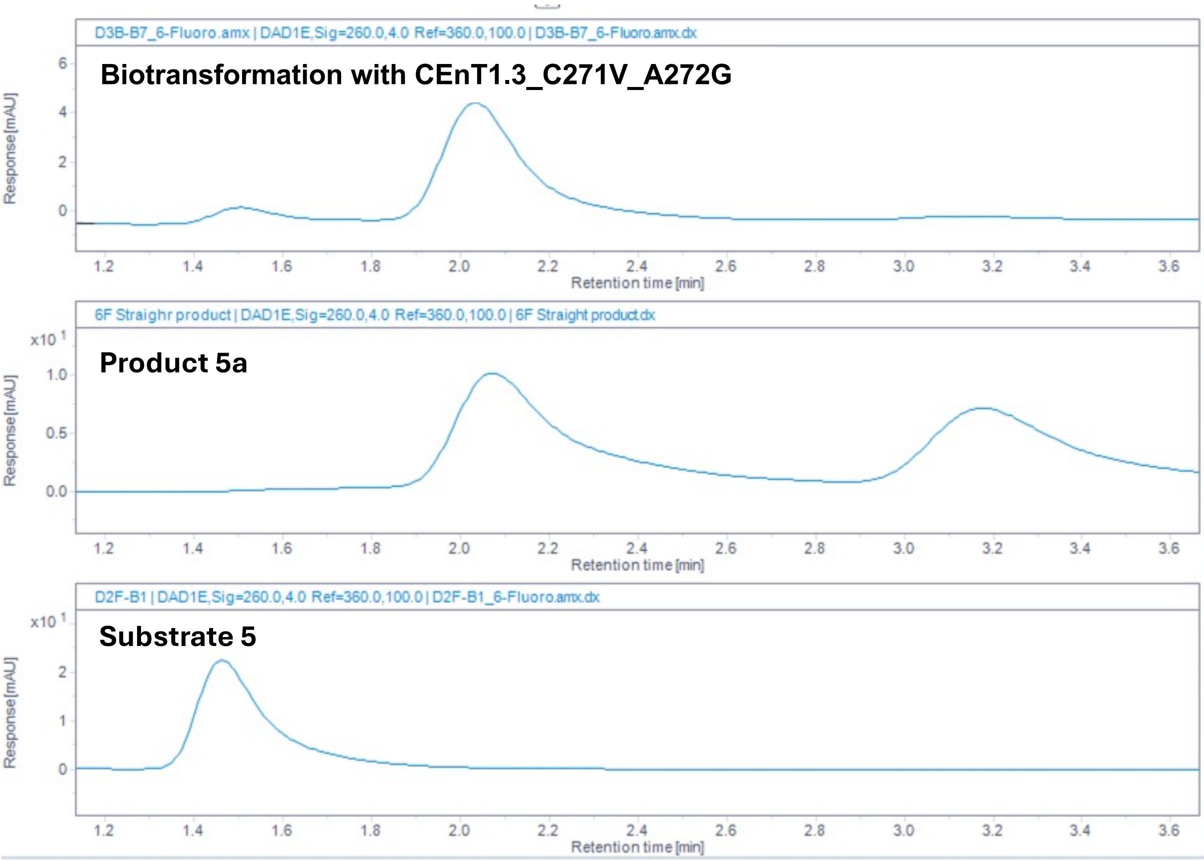

UPLC (IG-3 SFC column, 3 mm x 50 mm, Hexane/IPA=90:10). Substrate **5** elutes at 1.5 min while the two enantiomers of product **5a** elute at 2.1 and 3.2 min.

Substrate 6

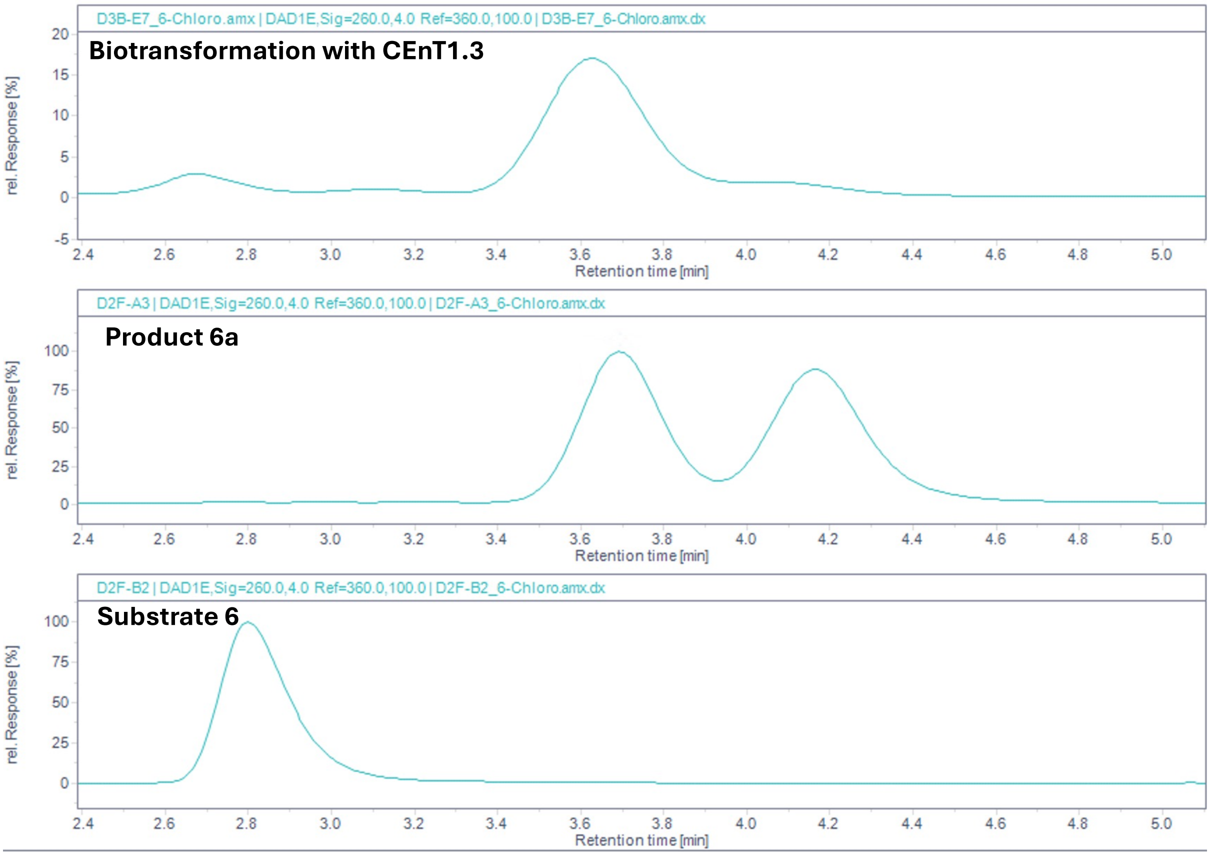

UPLC (IB N-3 SFC column, 3 mm x 50 mm, Hexane/IPA=88:12). Substrate **6** elutes at 2.8 min while the two enantiomers of product **6a** elute at 3.6 min and 4.2 min.

Substrate 7

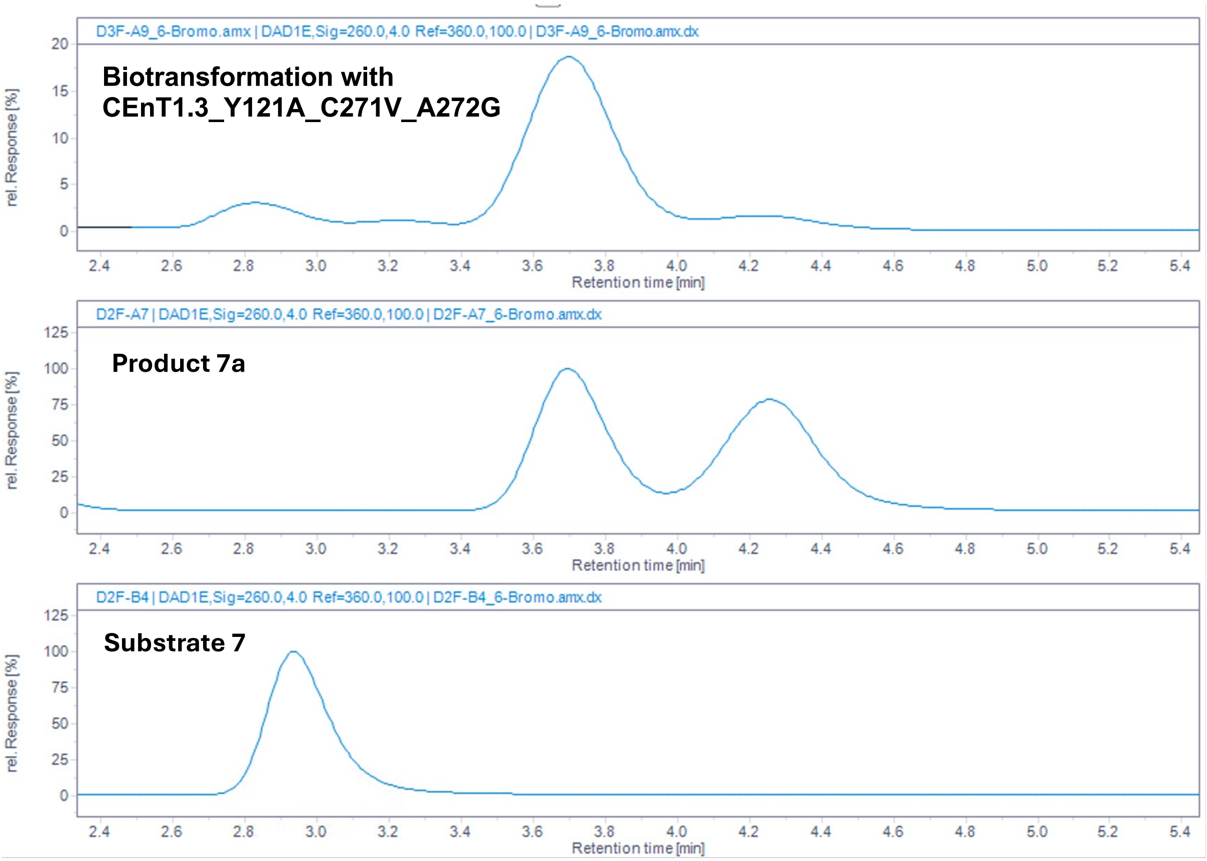

UPLC (IB N-3 SFC column, 3 mm x 50 mm, Hexane/IPA=88:12). Substrate **7** elutes at 2.8 min while the two enantiomers of product **7a** elute at 3.7 min and 4.2 min.

Substrate 8

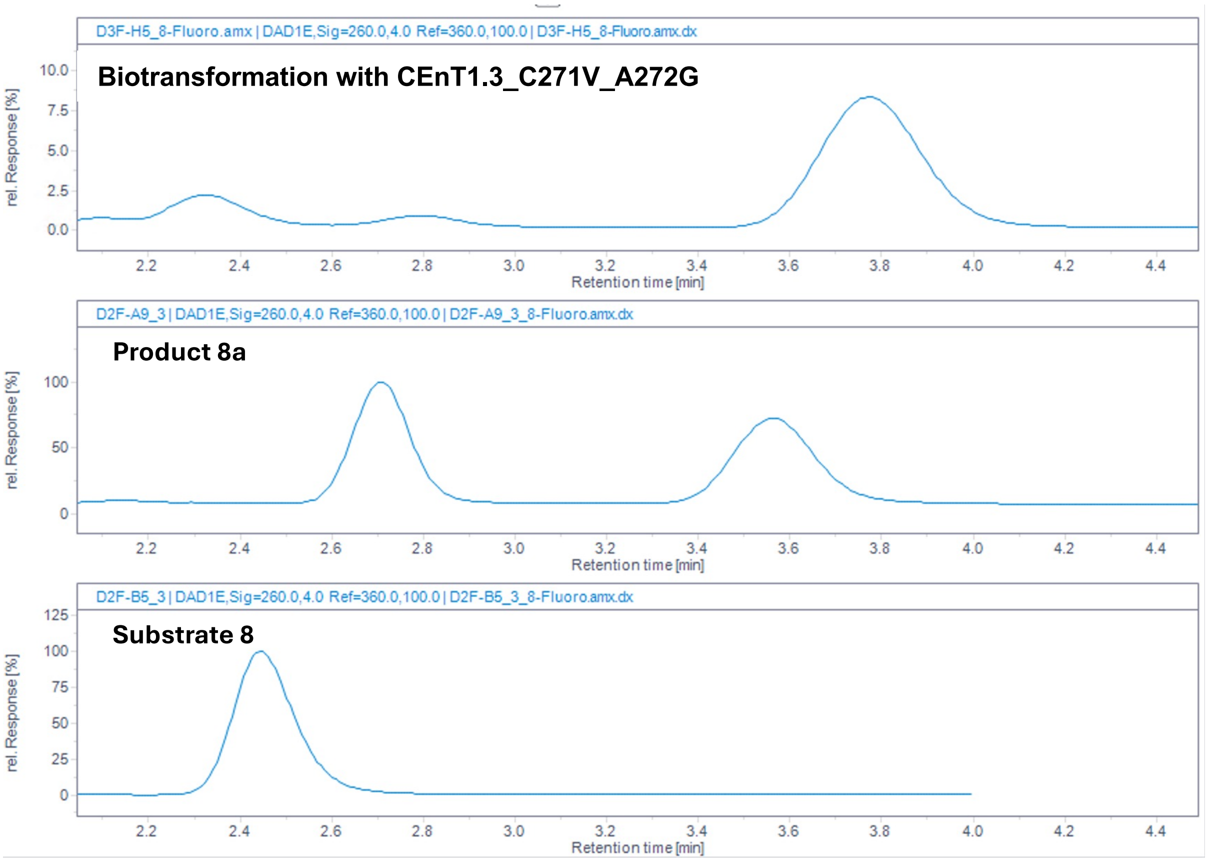

UPLC (IB N-3 SFC column, 3 mm x 50 mm, CO2/methanol=85:15). Substrate **8** elutes at 2.4 min while the two enantiomers of product **8a** elute at 2.8 min and 3.6 min.

Substrate 9

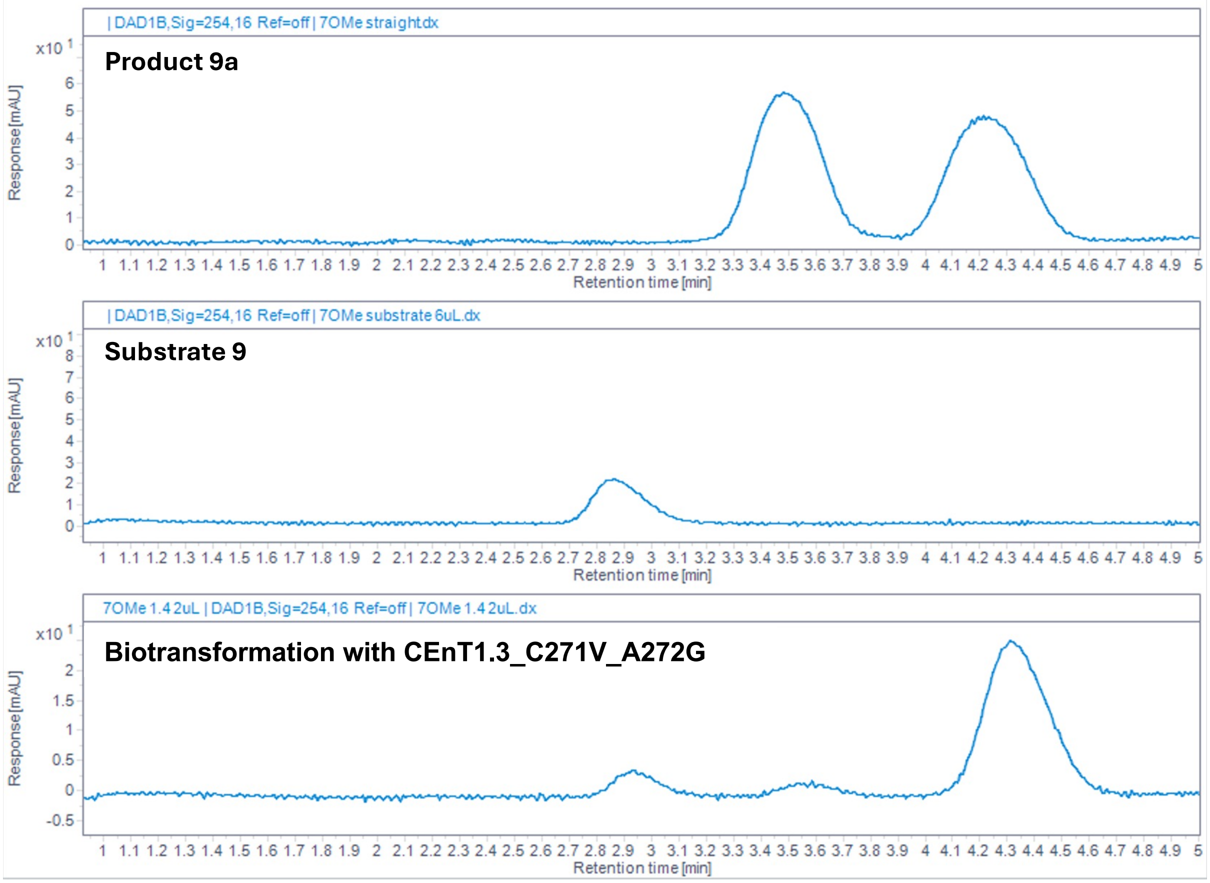

SFC (IC-3 SFC column, 3 mm x 50 mm, CO2/methanol=85:15). Substrate **9** elutes at 2.9 min while the two enantiomers of product **9a** elute at 3.6 min and 4.3 min.

References

[1] J. S. Trimble, R. Crawshaw, F. J. Hardy, C. W. Levy, M. J. B. Brown, D. E. Fuerst, D. J. Heyes, R. Obexer, A. P. Green, *Nature* **2022**, *611*, 709-714.

[2] J. W. Chin, A. B. Martin, D. S. King, L. Wang, P. G. Schultz, *Proc Natl Acad Sci U S A* **2002**, *99*, 11020-11024.

[3] J. B. Siegel, A. Zanghellini, H. M. Lovick, G. Kiss, A. R. Lambert, J. L. St Clair, J. L. Gallaher, D. Hilvert, M. H. Gelb, B. L. Stoddard, K. N. Houk, F. E. Michael, D. Baker, *Science* **2010**, *329*, 309-313.

[4] A. Pyo, S. Kim, M. R. Kumar, A. Byeun, M. S. Eom, M. S. Han, S. Lee, *Tetrahedron Lett.* **2013**, *54*, 5207-5210.

[5] P. D. Adams, P. V. Afonine, G. Bunkoczi, V. B. Chen, I. W. Davis, N. Echols, J. J. Headd, L. W. Hung, G. J. Kapral, R. W. Grosse-Kunstleve, A. J. McCoy, N. W. Moriarty, R. Oeffner, R. J. Read, D. C. Richardson, J. S. Richardson, T. C. Terwilliger, P. H. Zwart, *Acta Crystallogr D Biol Crystallogr* **2010**, *66*, 213-221.

[6] R. P. Joosten, K. Joosten, S. X. Cohen, G. Vriend, A. Perrakis, *Bioinformatics* **2011**, *27*, 3392-3398.

[7] M. J. Frisch, G. W. Trucks, H. B. Schlegel, G. E. Scuseria, M. A. Robb, J. R. Cheeseman, G. Scalmani, V. Barone, G. A. Petersson, H. Nakatsuji, X. Li, M. Caricato, A. V. Marenich, J. Bloino, B. G. Janesko, R. Gomperts, B. Mennucci, H. P. Hratchian, J. V. Ortiz, A. F. Izmaylov, J. L. Sonnenberg, D. Williams-Young, F. Ding, F. Lipparini, F. Egidi, J. Goings, B. Peng, A. Petrone, T. Henderson, D. Ranasinghe, V. G. Zakrzewski, J. Gao, N. Rega, G. Zheng, W. Liang, M. Hada, M. Ehara, K. Toyota, R. Fukuda, J. Hasegawa, M. Ishida, T. Nakajima, Y. Honda, O. Kitao, H. Nakai, T. Vreven, K. Throssell, J. A. Montgomery, Jr., J. E. Peralta, F. Ogliaro, M. J. Bearpark, J. J. Heyd, E. N. Brothers, K. N. Kudin, V. N. Staroverov, T. A. Keith, R. Kobayashi, J. Normand, K. Raghavachari, A. P. Rendell, J. C. Burant, S. S. Iyengar, J. Tomasi, M. Cossi, J. M. Millam, M. Klene, C. Adamo, R. Cammi, J. W. Ochterski, R. L. Martin, K. Morokuma, O. Farkas, J. B. Foresman, and D. J. Fox, *Gaussian 09* , Revision B.01; Gaussian, Inc.: Wallingford, CT, 2013.

[8] J. Eberhardt, D. Santos-Martins, A. F. Tillack, S. Forli, *J Chem Inf Model* **2021**, *61*, 3891-389.

[9] a) S. Pall, A. Zhmurov, P. Bauer, M. Abraham, M. Lundborg, A. Gray, B. Hess, E. Lindahl, *J Chem Phys* **2020**, *153*, 134110; b) M. J. Abraham, T. Murtola, R. Schulz, S. Páll, J. C. Smith, B. Hess, E. Lindahl, *SoftwareX* **2015**, *1-2*, 19–25.

[10] D. Case, K. Belfon, I. Ben-Shalom, S. Brozell, D. Cerutti, T. Cheatham, V. Cruzeiro, T. Darden, R. Duke, G. Giambasu, AMBER2020, university of California, San Fransisco. *J. Amer. Chem. Soc* **2020**, *142*, 3823-3835.

[11] a) Tian, C.; Kasavajhala, K.; Belfon, K. A. A.; Raguette, L.; Huang, H.; Migues, A. N.; Bickel, J.; Wang, Y.; Pincay, J.; Wu, Q.; Simmerling, C. Ff19SB: Amino-Acid-Specific Protein Backbone Parameters Trained against Quantum Mechanics Energy Surfaces in Solution. *Journal of Chemical Theory and Computation* **2019**, *16* (1), 528–552. b) A. A. Voityuk, *J. Phys. Chem. C* **2010**, *114*, 20236-20239.
